# SRF Co-factors Control the Balance between Cell Proliferation and Contractility

**DOI:** 10.1016/j.molcel.2016.10.016

**Published:** 2016-12-15

**Authors:** Francesco Gualdrini, Cyril Esnault, Stuart Horswell, Aengus Stewart, Nik Matthews, Richard Treisman

**Affiliations:** 1 Signalling and Transcription Group, Francis Crick Institute, 1 Midland Rd, London NW1 1AT, UK; 2 Bioinformatics and Biostatistics STP, Francis Crick Institute, 1 Midland Rd, London NW1 1AT, UK; 3 Advanced Sequencing STP, Francis Crick Institute, 1 Midland Rd, London NW1 1AT, UK

**Keywords:** transcription, transcription factors, Ras/MAPK signaling, cell proliferation, cell contraction, SRF, Ternary Complex Factor, Elk-1, immediate-early genes, Hi-C

## Abstract

The ERK-regulated ternary complex factors (TCFs) act with the transcription factor serum response factor (SRF) to activate mitogen-induced transcription. However, the extent of their involvement in the immediate-early transcriptional response, and their wider functional significance, has remained unclear. We show that, in MEFs, TCF inactivation significantly inhibits over 60% of TPA-inducible gene transcription and impairs cell proliferation. Using integrated SRF ChIP-seq and Hi-C data, we identified over 700 TCF-dependent SRF direct target genes involved in signaling, transcription, and proliferation. These also include a significant number of cytoskeletal gene targets for the Rho-regulated myocardin-related transcription factor (MRTF) SRF cofactor family. The TCFs act as general antagonists of MRTF-dependent SRF target gene expression, competing directly with the MRTFs for access to SRF. As a result, TCF-deficient MEFs exhibit hypercontractile and pro-invasive behavior. Thus, competition between TCFs and MRTFs for SRF determines the balance between antagonistic proliferative and contractile programs of gene expression.

## Highlights

• Integrated ChIP-seq Hi-C analysis identifies over 700 TCF-dependent SRF target genes
• Over 60% of TPA-inducible gene transcription is TCF-dependent
• TCF-dependent transcription potentiates cell proliferation
• TCF/MRTF competition for SRF determines contractility and pro-invasive behavior

## Graphical Abstract

**Figure:**
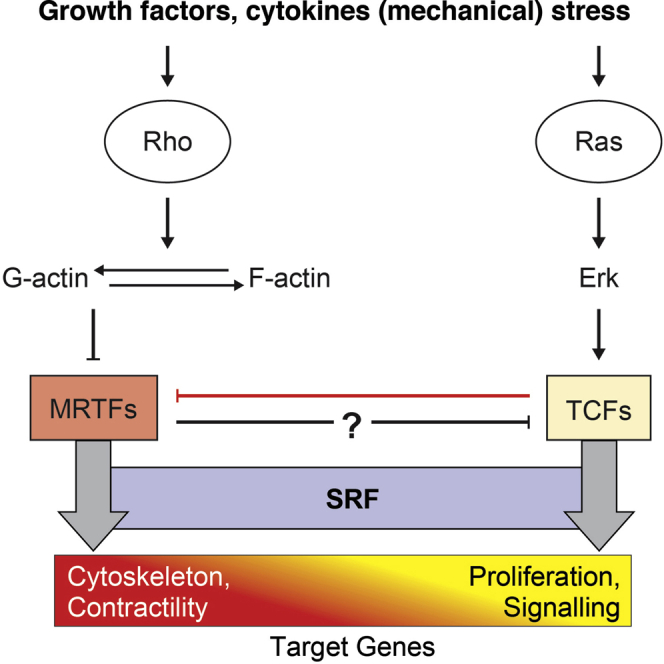

## Teaser

Two cofactor families, the TCFs and the MRTFs, compete for binding to SRF, a key regulator of the mitogen-responsive transcription. TCFs not only control proliferative gene expression programs but also regulate access of the MRTFs to SRF, thereby regulating cell contractility and pro-invasive behavior.

## Introduction

Ras-ERK signaling is critical for control of proliferation, invasion, and metastasis. Ras activation stimulates cell-cycle re-entry from quiescence, and Ras genes are mutated in around 20% of human cancers (Chambard et al., 2007, Pylayeva-Gupta et al., 2011). Ras-ERK signaling plays an important role in this immediate-early (IE) transcriptional response to mitogen stimulation, which activates many genes encoding transcription factors, including Myc and members of the AP1 and Egr families (Cochran et al., 1984, Greenberg and Ziff, 1984, Kelly et al., 1983). Many IE genes are controlled by the transcription factor serum response factor (SRF, *Srf*), which is required for Ras-induced cell-cycle re-entry but not for proliferation per se (Gauthier-Rouvière et al., 1990, Schratt et al., 2001).

SRF acts in partnership with two families of signal-regulated cofactors. The three ternary complex factors (TCFs) Elk-1, Net, and SAP-1 (*Elk1*, *Elk3*, and *Elk4*) are Ets domain proteins, regulated by Ras-ERK signaling (Buchwalter et al., 2004, Shaw et al., 1989), whereas the myocardin-related transcription factors (MRTFs) *Mkl1* and *Mkl2* respond to the Rho-actin pathway (Miralles et al., 2003, Olson and Nordheim, 2010). The TCFs and MRTFs interact competitively with the SRF DNA-binding domain (Miralles et al., 2003, Zaromytidou et al., 2006). Whether cofactor competition is a general feature of SRF regulation in vivo has been unclear. In fibroblasts, some IE genes appear to be specifically coupled to one pathway or the other, but in smooth muscle cells, platelet-derived growth factor (PDGF) can induce cofactor exchange (Wang et al., 2004).

Genetically, the TCFs are at least partly functionally redundant (Costello et al., 2010, Weinl et al., 2014) and may also function independently of SRF (Boros et al., 2009a, Boros et al., 2009b, Buchwalter et al., 2005). Although they have been implicated in proliferation and cancer (Vickers et al., 2004, Wozniak et al., 2012, Yang et al., 2012), the extent to which the immediate-early transcriptional response is TCF-dependent, and the target genes involved, has not been systematically investigated. In contrast, the MRTFs mediate morphogenetic, adhesive, and motile processes (Miralles et al., 2003, Olson and Nordheim, 2010, Schratt et al., 2002).

We showed previously that much of the serum-induced immediate transcriptional response is MRTF/SRF-dependent (Esnault et al., 2014); however, the lack of specific TCF inhibitors and the relatively poor quality of TCF chromatin immunoprecipitation (ChIP) data precluded rigorous analysis of the role of TCF-SRF signaling. Here we used wild-type and triply TCF-deficient mouse embryonic fibroblasts (MEFs) to directly address the role of the TCFs in the transcriptional response to 12-O-tetradecanoyl phorbol-13-acetate (TPA)-induced ERK activation. We show that the majority of the immediate transcriptional response is TCF-dependent, either directly or indirectly. TCF-deficient MEFs proliferate slowly, and TCF-dependent SRF target genes, which include *Myc,* are predominantly associated with cell signaling, metabolism, and proliferation. Strikingly, the TCFs also act as general negative regulators of cell adhesion, contractility, and motility by inhibiting access of MRTFs to SRF at its target genes.

## Results

### TPA Treatment Both Activates and Downregulates Gene Transcription

**Figure 1:**
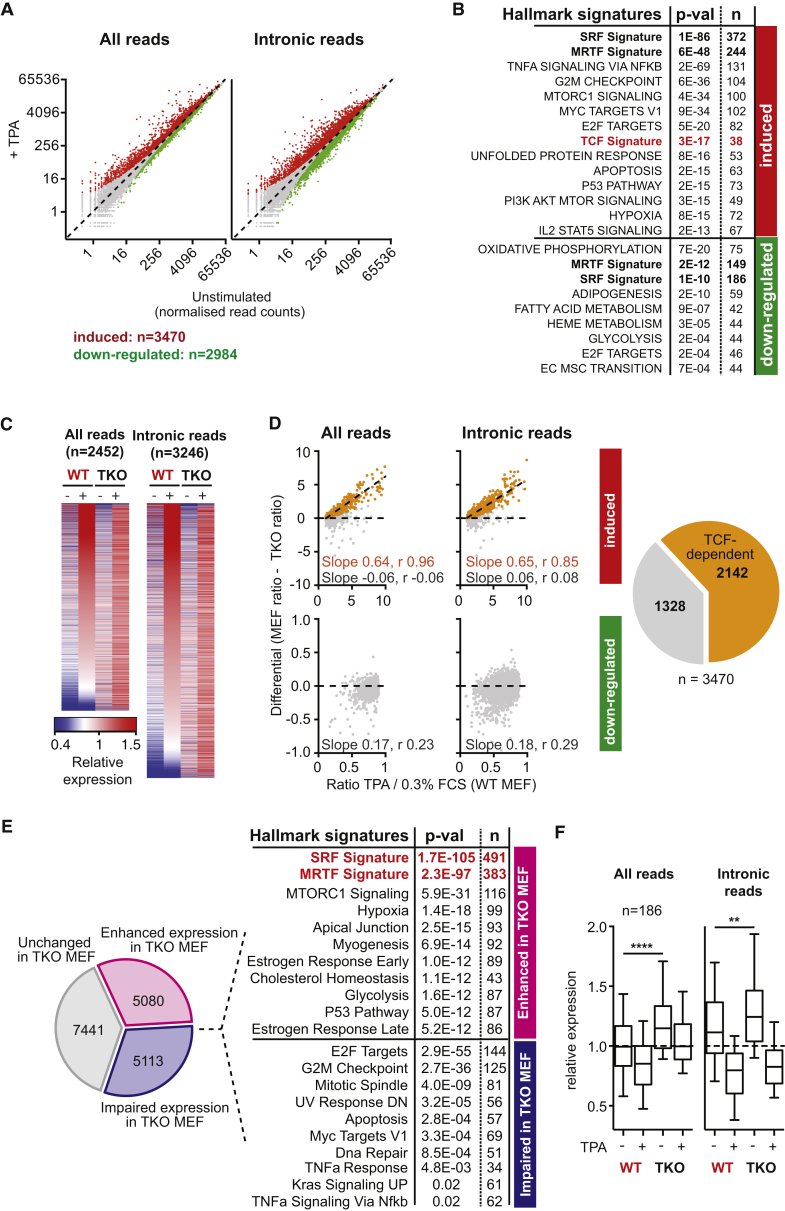
Much of the TPA-Induced Transcriptional Response Is TCF-Dependent (A) Scatterplot displaying total and intronic RNA-seq read counts before and after 30 min of TPA stimulation. Induced genes are highlighted in red and downregulated genes in green (FDR = 0.01, fold change ≥ 10%). (B) The relation between TPA-induced and -downregulated genes and MsigDB hallmark gene set signatures was assessed by hypergeometrical test. p-val, Bonferroni-adjusted p value. (C) Heatmap representation of the relative expression of TPA-induced genes in wild-type and TKO MEFs ranked by absolute change in transcription induced by TPA in wild-type MEFs. (D) Identification of TCF-dependent genes by comparison with the TPA induction ratio in wild-type and TKO MEFs using both total and intronic RNA-seq reads. TCF-dependent genes (orange) exhibit a systematic relationship between their degree of induction in the two contexts whereas others (gray) do not; slope and Spearman r are indicated. Right: data summary. (E) Changes to basal gene expression in TKO MEFs. The relation between genes whose basal expression is increased (top) or decreased (bottom) in TKO MEFs and MsigDB hallmark gene set signatures was assessed as in (B). (F) TPA-downregulated SRF targets are TCF-independent but exhibit increased baseline expression in TKO MEFs. Boxplots present the average fold change of total and intronic RNA. Center line, median; top and bottom edges, 75th and 25th percentiles. ^∗∗∗∗^p < 0.0001, ^∗∗^p < 0.01 (Wilcoxon matched pair signed-rank test). See also Figure S1 and Tables S1 and S2.

To activate TCF-SRF signaling in MEFs, we used the phorbol ester TPA, which activates ERK via protein kinase C (PKC) and RasGRP1 (Griner and Kazanietz, 2007). TPA also downregulates Rho-actin signaling (Panayiotou et al., 2016) and thus allows the identification of genes that are particularly sensitive to TCF activation. In immortalized MEFs, TPA activated classical TCF-SRF targets such as *Egr1* but not MRTF-SRF target genes such as *Vcl*, although all were activated by serum stimulation (Figure S1A). We used RNA sequencing (RNA-seq) to analyze the global response to TPA stimulation, analyzing both total and intronic RNA-seq reads to maximize sensitivity (Figure 1A; Table S1). The TPA-induced gene set is enriched in gene hallmarks (i.e., gene cohorts that change similarly in different contexts; Subramanian et al., 2005) associated with the cell cycle, signaling networks, and particular transcription factors (Figure 1B; Table S2A) and gene ontology (GO) terms for genes involved in signaling, metabolism, the cell cycle, and the cytoskeleton (Table S2B). It was also significantly enriched for genes from both the TCF- and MRTF-SRF gene signatures (Esnault et al., 2014; Figure 1B), suggesting that many MRTF-SRF target genes are also directly or indirectly controlled by the TCFs (see below). In contrast, TPA-downregulated genes were associated with hallmarks involving specific cellular functions, particularly metabolism; MRTF-SRF target genes were also enriched in this group, consistent with TPA downregulating Rho (Figure 1B; Tables S2A and S2B; see below).

### TCFs Promote Cell Proliferation and Control Most TPA-Induced Transcription

To investigate the role of TCFs in gene expression and cell behavior, we studied MEFs from embryos lacking all three TCFs (triple knockout [TKO] MEFs; Costello et al., 2010). Strikingly, TKO MEFs were significantly less proliferative than wild-type MEFs (Figure S1B), with increased numbers of cells in G2/M compared with wild-type cells (Figure S1C). They also exhibited somewhat enhanced ERK activation in response to TPA (Figure S1D), probably reflecting aberrant expression of ERK phosphatases (Buffet et al., 2015). Nevertheless, TPA induction of classical TCF-linked immediate-early genes was effectively abolished in TKO MEFs (Figure S1E), although serum induction of SRF target genes was less impaired (Figures S1A, S1E, and S1F).

RNA-seq analysis revealed that the global transcriptional response to TPA was substantially altered in TKO MEFs (Figure 1C). Using an iterative pipeline to compare gene inducibility in the two different backgrounds, we partitioned the genes induced by TPA in wild-type MEFs into TCF-dependent and -independent groups (Supplemental Experimental Procedures). Over 60% of TPA-induced gene expression was TCF-dependent (estimated at 2,142 of 3,470), whereas TPA-downregulated gene expression showed no obvious TCF-dependence (Figure 1D). Thus, consistent with their known biochemical role in gene activation, the TCFs act positively in the transcriptional response to TPA stimulation.

TCF inactivation also changed the basal expression of approximately 60% of genes detectably transcribed in wild-type MEFs, regardless of their acute response to TPA stimulation (Figure 1E). Consistent with the proliferative defect observed in TKO cells, genes with reduced basal expression were enriched in gene hallmarks and GO terms associated with cell-cycle and proliferative responses (Figure 1E; Tables S2A and S2B). In contrast, genes exhibiting increased basal transcription in TKO cells included a substantial number of genes previously identified as MRTF-SRF targets (383 of 683; Esnault et al., 2014) predominantly associated with cytoskeletal hallmarks and GO terms (Figure 1E; Tables S2A and S2B). Many of these genes were downregulated in response to TPA in wild-type MEFs (Figure 1F). Thus, the TCFs not only mediate the acute response to ERK activation but also negatively regulate MRTF-SRF signaling (see below).

### Integration of SRF ChIP-Seq and Hi-C Datasets Identifies Candidate SRF Target Genes

**Figure 2:**
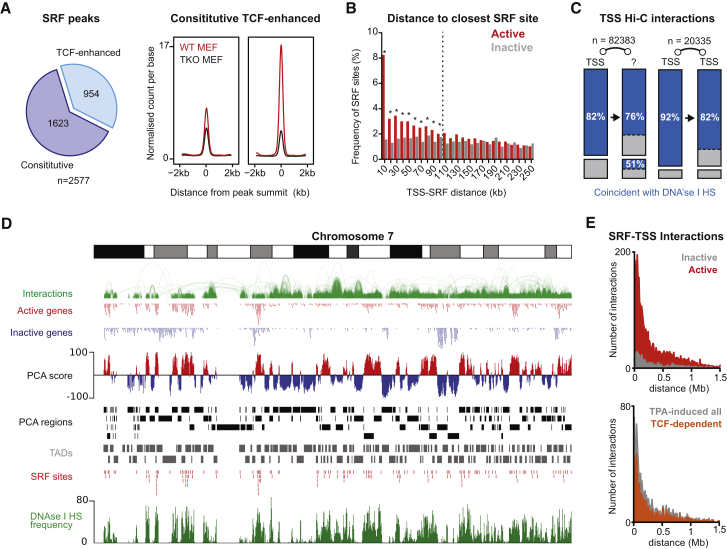
Integration of SRF ChIP-Seq in MEFs with Hi-C Interaction Data (A) The TCFs enhance SRF binding at many sites. Metaprofiles of the SRF ChIP-seq signal at sites where SRF binding is unaffected by TCFs (constitutive) or where they enhance it (TCF-enhanced). (B) Comparison of the frequency of SRF sites at increasing distances from active and inactive gene TSSs (10-kb bins). ^∗^, significant at p < 0.05 (multiple t test with Holmes-Sidak correction). (C) Most TSS Hi-C interactions in MEFs coincide with DNase I HS at 10-kb resolution. Left: TSS interactions with non-TSS regions. Right: TSS-TSS interactions. Blue, interactions involving regions containing at least one DNase I HS. (D) Hi-C analysis. Chromosome 7 is shown with tracks as follows: significant Hi-C interactions displayed using the WashU EpiGenome browser; active (red) and inactive (blue) RefSeq-annotated genes identified by RNA-seq; PCA analysis of Hi-C data with A regions (positive PCA score) shown in red and B regions (negative PCA score) in blue; comparison of individual PCA regions (black) with the TADs (gray) previously annotated in MEFs (Battulin et al., 2015); SRF binding sites; and DNase I HS frequency per base. (E) The relation between TSS-SRF interaction distance in Hi-C and gene activity defined by RNA-seq. Top: interactions involving active (red) or inactive (gray) TSSs. Bottom: interactions with TPA-induced TSSs; those whose induction is TCF-dependent are highlighted in orange. See also Figure S2.

To identify TCF-dependent SRF target genes, we set out to correlate the RNA-seq data with SRF ChIP sequencing (ChIP-seq). We first identified 2,577 high-confidence SRF binding sites (Figure 2A; Figure S2A; Table S3). In TKO MEFs, ∼40% of these showed reduced SRF binding and increased H3 occupancy, suggesting that cooperative TCF-SRF binding facilitates nucleosome exclusion (Esnault et al., 2014; Figure S2B). SRF sites were statistically overrepresented within 100 kb of active genes in MEFs (Figure 2B), coinciding with DNase I-hypersensitive sites (DNase I HS) (Figures S2C–S2E; see GEO: GSM1003831 and GSM1014199). They were associated with the same transcription factor (TF) binding motifs as in NIH 3T3 fibroblasts (Esnault et al., 2014), although, at TCF-enhanced sites, Ets motifs were more prevalent, suggesting that TCF-SRF interaction facilitates SRF binding (Figure S2F).

Integration of Hi-C chromosome interaction maps with ChIP-seq data can reliably identify signal-induced genes controlled by remote regulatory sites (Jin et al., 2013). We therefore integrated the SRF ChIP-seq data with recently determined Hi-C maps of intrachromosomal interactions in MEFs (Battulin et al., 2015, Minajigi et al., 2015). We combined the MEF Hi-C datasets using iterative mapping (Imakaev et al., 2012; Experimental Procedures), identifying 1.3 million Hi-C interactions at 10-kb resolution, of which around 100,000 linked RefSeq-annotated transcription start sites (TSSs) to remote, mostly non-TSS, regions containing DNase I HS (Figure 2C).

Using principal component analysis (PCA) (Lieberman-Aiden et al., 2009; Experimental Procedures), we partitioned the genome into regions of preferential Hi-C interaction (Figure 2D; Figures S2G and S2H). These regions, denoted A and B according to PCA score, encompass the topologically associating domains (TADs) defined previously in MEFs (Battulin et al., 2015) and are analogous to the “A and B compartments” defined previously at 1-Mb resolution (Lieberman-Aiden et al., 2009). The A regions correlated strongly with the presence of genes and with overall gene expression (Figure S2I). They also showed significant correlation with DNase I HS frequency, an independent indicator of gene activity (Figure S2J). Most importantly, the A regions contained over 80% of the SRF binding sites (2,137 of 2,577; Figure 2E; Figure S2K). Most TSSs linked to remote SRF binding sites by Hi-C were transcriptionally active, and most TPA-inducible TSSs linked to remote sites were also TCF-dependent (Figure 2E; Table S1), suggesting that the integrated SRF ChIP-seq/Hi-C method identifies functionally relevant TSS-SRF interactions.

### Definition of TCF-Dependent TPA-Induced SRF Target Genes

**Figure 3:**
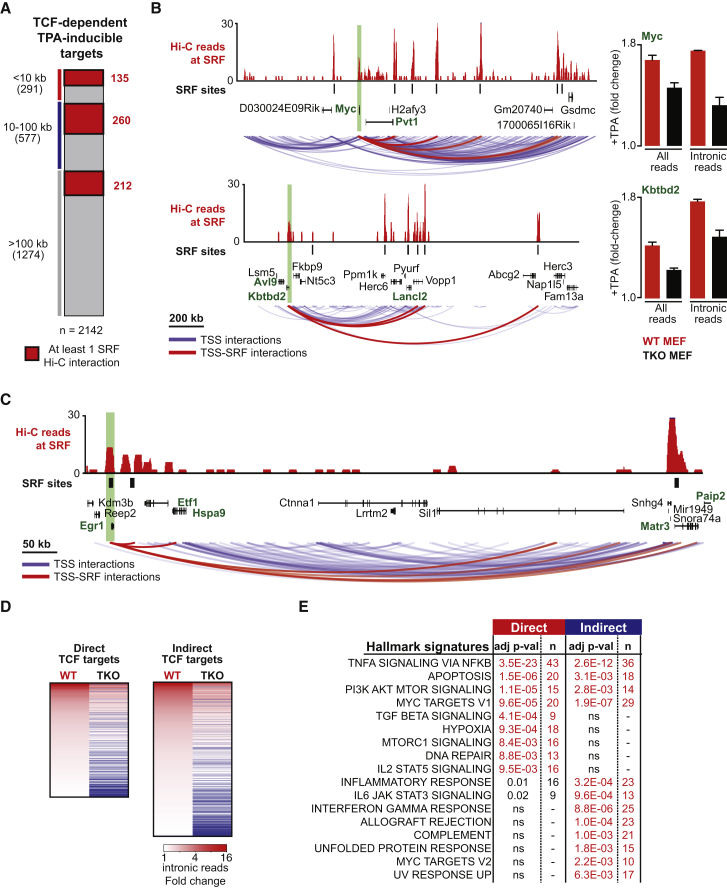
Genome-wide Identification of Direct SRF/TCF Target Genes (A) Definition of the TCF-dependent TPA-inducible gene set. The integrated SRF ChIP-seq Hi-C data are summarized according to the distance between inducible TSSs to the closest SRF site, with those TSSs and SRF sites displaying Hi-C interaction shaded in red (not all interactions within 10 kb of TSSs were detectable by Hi-C analysis). The 763 Direct TCF-SRF target genes are defined as those whose TSSs are within 10 kb of an SRF site or that interact with one at any distance, as judged by Hi-C. 1,062 Indirect TCF-dependent target genes are defined as those whose TSSs are >100 kb from an SRF site and exhibit no Hi-C interaction with one. (B) Remote-controlled SRF targets. The *Myc* and *Kbtbd2* loci are shown with Hi-C paired-end reads (10 kb bins) with ends mapping to SRF binding sites shown in red. SRF ChIP-seq binding sites and RefSeq-annotated genes are shown below, with TPA-induced genes shown in green. Significant Hi-C interactions by the *Myc* and *Kbtbd2* TSS are shown in blue, with those involving SRF sites shown in red. The effect of TCF inactivation on RNA-seq reads is shown at the right. (C) Interactions between the TCF-SRF direct target gene *Egr1* and remote SRF sites identified by Hi-C, displayed as in (B). (D) TPA induction is similar at both Direct and Indirect TCF target genes, as assessed by the fold change in intronic RNA-seq reads. (E) The relation between Direct and Indirect TCF target genes and MsigDB hallmark gene set signatures was assessed by hypergeometrical test (the number of genes and the Bonferroni-adjusted p value are shown). See also Figure S3 and Tables S3 and S4.

Of the 3,470 TPA-inducible genes, 1,231 had TSSs within 10 kb of an SRF site and/or interacted with a remote SRF site in Hi-C (Figure S3A). Activation of 763 was TCF-dependent, so we defined them as high-confidence Direct TCF-SRF targets (Figure 3A; Table S1). The majority of these (472 of 763) had closest interacting SRF sites >10 kb distant, in many cases >100 kb away, and many exhibited multiple long-distance interactions with SRF, including *Kbtb2* and *Myc*, which interacts with five remote SRF sites, the closest at ∼200 kb (Figures 3B and 3C; Table S1; Discussion). TPA-induced chromatin modification at the Myc TSS region is also TCF-dependent (data not shown; C.E., F.G., S.H., A.S., N.M., R.T., G. Kelly, and P. East, unpublished data). The *Egr1* TSS region, which contains multiple SRF sites, also interacted with a putative remote SRF-linked enhancer, the TPA-inducible TCF-dependent *Etf1* gene ∼70 kb downstream, and other more distant genes (Figure 3C). In all, 174 “remote-controlled” TSSs were linked to SRF sites that were themselves close to TSSs, blurring the distinction between “enhancer” and “promoter” elements (Figures 3B and 3C; Table S1).

The integrated Hi-C/ChIP-seq data allowed definition of a high-confidence set of 1,062 indirectly TCF-dependent TPA-inducible genes that have TSSs that are neither near SRF sites nor physically interact with them in Hi-C (Figure 3D; see below). Gene set enrichment analysis using the MsigDB database showed that the TCF-dependent Direct and Indirect gene sets were related but distinct (Figure 3E; Tables S4A and S4B). Similar to the TPA-induced population as a whole, Direct genes were enriched in gene hallmarks involved in the cell cycle, signaling networks, and particular transcription factors (Table S4A) and GO categories related to signaling, metabolism, the cell cycle, and the cytoskeleton (Table S4B).

Interestingly, the TCF-dependent Direct gene set contained 100 genes shown previously to be MRTF-SRF targets in serum-stimulated NIH 3T3 fibroblasts (Esnault et al., 2014; Figure S3A; Table S1). This “shared MRTF-TCF” gene set was enriched in gene hallmarks and GO terms involving the cytoskeleton, whereas the remaining “TCF-only” gene set was enriched in proliferative and signaling gene hallmarks and GO terms (Tables S4A and S4B). Finally, approximately 30% of genes whose basal expression was decreased upon TCF inactivation were also physically linked or close to SRF binding sites (1,501 of 5,113; Figure S3B), suggesting that they respond directly to basal levels of TCF activity. The role of the TCFs in the regulation of genes exhibiting enhanced basal activity in TKO MEFs will be considered below.

### Elk-1 Re-expression Partially Restores Regulation to TCF-Dependent Target Genes

**Figure 4:**
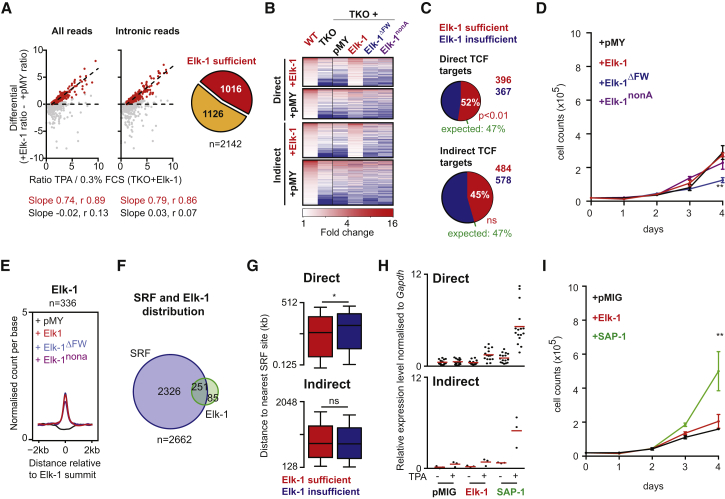
Elk-1 Acts with SRF to Restore TCF-Dependent Gene Expression in TKO MEFs (A) Identification of genes whose induction in TKO MEFs is restored by expression of wild-type human Elk-1. The plots compare the TPA induction ratio in TKO MEFs with and without Elk-1 expression for both total and intronic RNA-seq reads. Genes whose induction is restored by Elk-1 (red) exhibit a systematic relationship between their inducibility in the two contexts whereas others (gray) do not; slope and Spearman r are indicated. Right: summary. (B) Heatmap representation of TPA-induced gene expression (fold change over baseline) in wild-type and TKO MEFs and TKO MEFs expressing the indicated Elk-1 derivatives. Genes whose regulation is restored by wild-type Elk-1 are shown, divided into Direct and Indirect categories. The plot is ranked by the magnitude of the TPA-induced changes in wild-type MEFs. (C) Proportions of Direct and Indirect TCF target genes whose regulation by TPA is restored by Elk-1 expression. Restoration of Direct, but not Indirect, targets is significantly above the 47% expected from the overall restoration of TCF targets (two-tailed binomial test, ^∗^p < 0.05). (D) Expression of human does not restore the proliferation defect observed in TKO MEFs. Cell counts for TKO MEFs expressing the indicated proteins were recorded over 4 days. (E) Metaprofiles showing the average normalized count per base across the 336 Elk-1 binding sites identified by ChIP-seq in TKO MEFs reconstituted with Elk-1 derivatives as indicated. (F) Coincidence between SRF and Elk-1 ChIP-seq peaks. (G) Direct TCF-SRF targets whose regulation is restored by Elk-1 expression are closer to SRF sites than those whose regulation is not. Mann-Whitney test, ^∗^p < 0.05. (H) Elk-1 transactivation is weaker than that of SAP-1. TPA induction of 16 direct TCF targets (*Egr1*, *Egr2*, *Egr3*, *Ier2*, *Per1*, *Junb*, *Fos*, *Zfp36*, *Dnajb1*, *Arl5*, *Pitpna*, *Mob3a*, *Pramef8*, *Tada3*, *Argap1*, and *Arpc4)* and three indirect targets (*Ier3*, *Klf10*, and *Cxcl1*) was analyzed by qRT-PCR. (I) SAP-1 expression restores proliferation in TKO MEFs. Cells were counted over 4 days. Two-way ANOVA, ^∗∗^p < 0.01. See also Figure S4 and Tables S5 and S6.

To assess to what extent TPA-induced gene expression reflects the action of the ERK-regulated TCF activation domain, we reconstituted TKO MEFs with derivatives of the human Elk-1 TCF (Figure S4A). Re-expression of wild-type Elk-1 but not the transcriptionally inactive mutants Elk-1^nonA^ and Elk-1^ΔFW^ (Balamotis et al., 2009, Buchwalter et al., 2004, Buchwalter et al., 2005) restored significant TPA-inducibility to many Direct and Indirect TCF target genes (Figures 4A and 4B; Table S5). Approximately half of the TPA-inducible TCF-dependent genes (1,016 of 2,142) appeared to be Elk-1 responsive (Figure 4B; Figure S4B). Elk-1 expression preferentially restored the activity of Direct TCF targets, and left TCF-independent genes unaffected (Figure 4C; Figure S4B). Elk-1-responsive genes were enriched for gene hallmarks and GO terms associated with proliferation, cell-cycle control, chromosomal replication, and segregation (Tables S4A and S4B), although Elk-1 expression failed to restore normal proliferation to reconstituted TKO MEFs (Figure 4D). Thus Elk-1, and presumably the other TCFs, is directly involved in control of proliferative gene expression.

### The Elk-1 TCF Functions with SRF in the TPA Response

To gain insight into the extent to which Elk-1’s activity is SRF-dependent as opposed to autonomous (Boros et al., 2009a, Boros et al., 2009b), we carried out ChIP-seq in reconstituted TKO MEFs (Experimental Procedures). We identified 336 high-confidence Elk-1 binding sites associated with CArG and ETS motifs, bound equally well by the Elk-1^nonA^ and Elk-1^ΔFW^ mutants, and unaffected by TPA stimulation (Figure 4E; Figure S4D, Table S3). Of these Elk-1 sites, 251 coincided with SRF peaks, whereas 85 were apparently SRF-independent “solo” sites (Figure 4F). In general, the SRF-associated Elk-1 peaks coincided with DNase I HS and were located proximal to TSSs, whereas the solo Elk-1 sites were not (Figures S4F and S4G; Table S3).

Approximately 25% of the detectable SRF/Elk-1 binding sites (89 of 356) were associated with the Direct TCF-SRF target genes, and a further 30 were associated with TPA-inducible SRF target genes that were not scored as TCF-dependent (Figure S3B; Table S3). In contrast, only 2.5% of Elk-1 solo binding sites (9 of 356) were associated with TPA-inducible genes (Table S4A). Elk-1 re-expression in TKO MEFs restored TPA inducibility to genes associated with SRF/Elk-1 sites (94 of 151) or solo Elk-1 sites (3 of 9; *Abce1*, *Trpc7*, and *Hat1*) (Table S5). Thus, Elk-1 acts predominantly in partnership with SRF to control TPA-inducible gene expression.

Next we sought to understand why Elk-1 expression only partially restored TCF-dependent TPA induction. Although it cannot be ruled out that this reflects gene-specific targeting of Elk-1, we found that the “rescued” genes had TSSs that were in general significantly closer to SRF sites than genes that were refractory to Elk-1 expression (Figure 4G). Previous studies have suggested that strong transcriptional activators work more effectively over distance than weak ones (Carey et al., 1990). We therefore compared the strength of transcription activation by Elk-1 with that of its relative SAP-1, with which it is functionally redundant (Costello et al., 2010). SAP-1 was significantly more effective than Elk-1 in restoring TPA-inducibility to 19 selected TCF-SRF target genes in TKO MEFs (Figure 4H; Figure S4A) and also substantially enhanced TKO MEF proliferation (Figure 4I). Taken together, these results suggest that the partial rescue of TCF-SRF target genes by Elk-1 expression in TKO MEFs at least partly reflects its relatively weak transcriptional activation capacity.

### Indirect and Direct TCF-Dependent Target Genes Are Linked through Transcriptional Regulation

**Figure 5:**
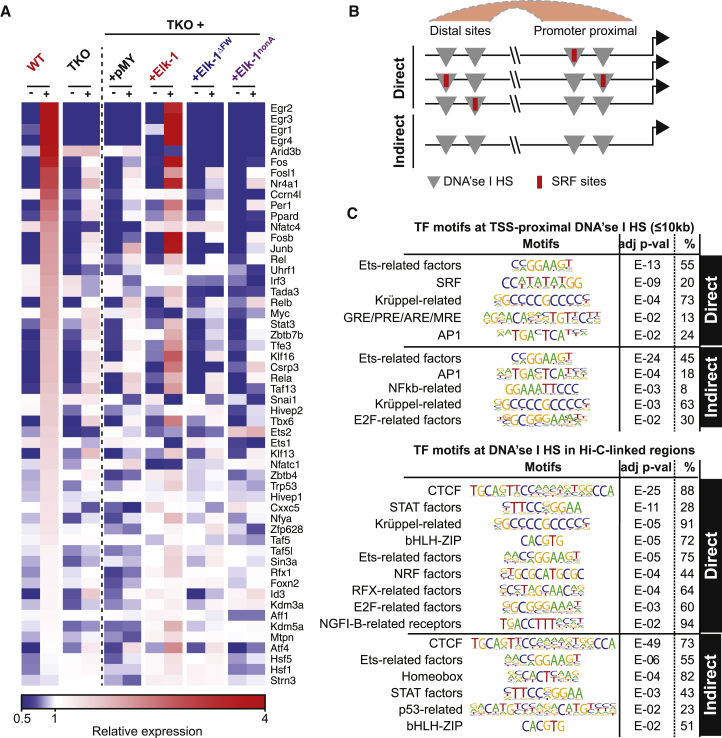
Indirect and Direct TCF-Dependent Target Genes Are Linked by Transcriptional Regulation (A) Heatmap representation of TPA-induced transcription of 54 transcription factor genes from the Direct TCF target gene set, ranked by magnitude of the TPA-induced changes in wild-type MEFs. (B) Potential relationships between DNase I HS (gray) and SRF binding sites (red) at TSS-proximal or remote regulatory sites of Direct and Indirect TCF target genes, with Hi-C linkages indicated by the crescent. (C) TF motifs occurring in putative regulatory regions (DNase I HS summit ± 100 bp) of Direct and Indirect TCF target genes. Motifs scoring as significantly enriched relative to their representation across all DNase I HS-associated sequences are shown together with their frequency. CTCF was recovered at all distal motifs, presumably reflecting its association with the insulator elements that define compartment boundaries. See also Table S6.

The expression of Indirect TCF-dependent target genes must either rely on basal levels of proteins encoded by direct TCF-SRF target genes, or reflect a secondary response to transcriptional regulators produced by immediate-early genes. The Direct TCF-SRF target gene set includes 54 different transcription factors, many of which are themselves sensitive to ERK activation (for references, see Table S6). These include members of the Egr, AP-1, Ets, NF-κB, and STAT families in addition to Myc, p53, and others (Figure 5A), and the Direct gene set is indeed enriched in gene hallmarks associated with these transcription factors (Tables S4A and S4B).

To investigate the relation between Indirect and Direct TCF target gene regulation, we examined their potential regulatory sequences. Many of these are likely to coincide with DNase I HS, so we performed HOMER motif analysis on all DNase I HS sequences located within 10 kb of or linked to Indirect TSSs by Hi-C, scoring only motifs over-represented with respect to the entire DNase I HS sequence population (Figures 5B and 5C). At TCF targets, TSS-proximal sites were enriched in SRF, Ets, AP-1, NF-κB, Kruppel-related (i.e., Egr-like), and E2F motifs, whereas distal sites were associated with Ets, Homeobox, STAT, p53, and E-BOX motifs; as expected, the SRF consensus was not enriched at Indirect targets (Figure 5C). These data support the view that products of Direct TCF-SRF target genes are required for the response of Indirect TCF targets to TPA stimulation.

### TCF Binding Antagonizes MRTF-SRF Signaling

**Figure 6:**
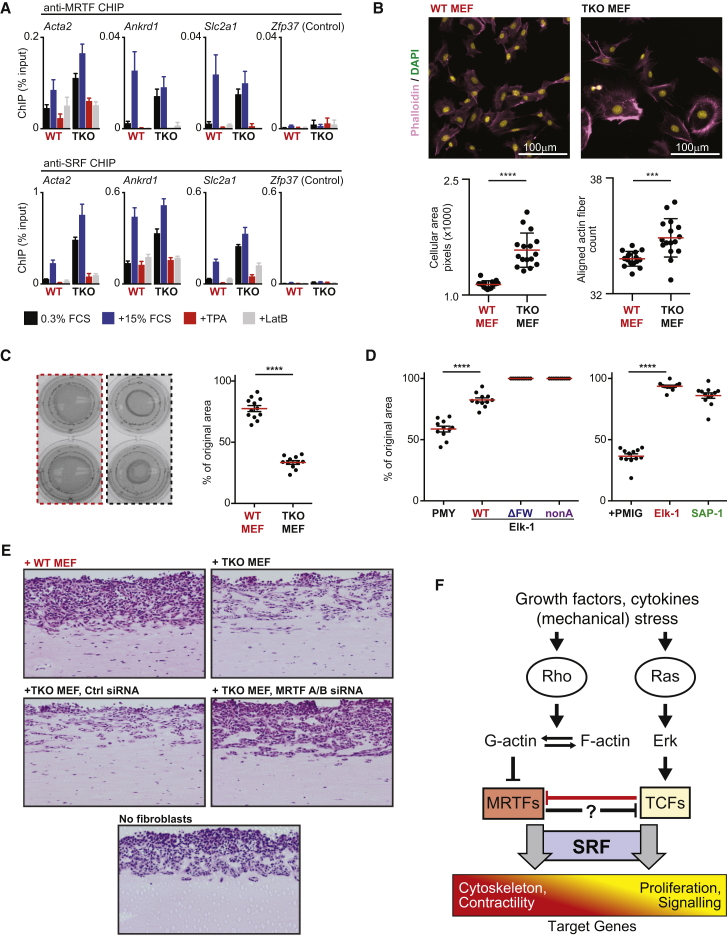
TCF Binding Directly Antagonizes MRTF-Dependent Gene Expression (A) Quantitative ChIP analysis of MRTF-A and SRF binding at regulatory elements of *Acta*2, *Ankrd1*, *Slc2a1*, and *Zfp37* negative control in wild-type and TKO MEFs either resting (0.3% FCS, black) or treated with serum (30 min, blue), TPA (30 min, black), or Latrunculin-B (5 min, gray). Error bars show SEM (n = 4). (B) TKO MEFs are large and contain more aligned actin fibers. Cells were stained for F-actin (Phalloidin, magenta) and DNA (DAPI, blue), and the cellular area and aligned actin fibers were quantified (n = 16, >3,000 cells/condition). Read line, mean per set of replicates. (^∗∗∗∗^p < 0.0001, ^∗∗∗^p < 0.001, Mann-Whitney test). (C) TKO MEFs are highly contractile. Left: representative replicate gel contraction wells containing wild-type (red) or TKO (black) MEFs. Right: quantitation (n = 12; red bar, mean; ^∗∗∗∗^p < 0.0001; Mann-Whitney test). (D) TKO MEF hypercontractility is suppressed by expression of either wild-type Elk-1, its two transcriptionally inactive derivatives Elk-1^FW^ and Elk-1^nonA^, or SAP-1. Quantitation is as in (C). (E) Representative images showing the invasion of 4T1 breast carcinoma cells into Matrigel containing no added fibroblasts, wild-type MEFs, or TKO MEFs with or without MRTF-A/B or control siRNAs. (F) Direct competition model for antagonism between MRTF-SRF- and TCF-SRF-dependent gene expression programs. Competition between the pathways for shared targets is indicated by diagonal shading. See also Figure S5.

We saw above that many genes exhibit increased basal transcription in TKO MEFs (Figure 1D; Table S1), including hundreds previously identified as MRTF-SRF target genes in NIH 3T3 cells (Esnault et al., 2014). Indeed, in TKO MEFs, the enhanced transcription of MRTF-SRF targets such as *Acta2*, *Ctgf*, and *Dstn* was inhibited by Latrunculin B (LatB), which inhibits MRTF (Miralles et al., 2003; Figure S5A). Moreover, quantitative ChIP showed substantially increased basal levels of MRTF recruitment to SRF targets such as *Acta2*, *Ankrd1*, and *Slc2a1,* which was inhibited by LatB or TPA, which inhibit Rho-actin signaling (Miralles et al., 2003), but further increased by serum stimulation (Figure 6A; Figure S5A). TKO MEFs also exhibited increased MRTF access to TCF-SRF direct target IE promoters in wild-type cells (Figure S5B). Thus, TCF inactivation potentiates MRTF-SRF signaling.

Finally we investigated the functional consequences of the increased MRTF-SRF signaling in TKO MEFs. TKO MEFs were larger than wild-type cells, contained increased numbers of parallel F-actin fibers (Figure 6B), and were significantly more contractile than wild-type MEFs, as assessed by the ability to contract collagen gel (Figure 6C). Re-expression of wild-type or transcriptionally inactive Elk-1 derivatives, or wild-type SAP-1 effectively suppressed the hypercontractile phenotype (Figure 6D). Pro-invasive behavior is associated with hypercontractility in cancer-associated fibroblasts (Calvo et al., 2013, Gaggioli et al., 2007). Accordingly, TKO MEFs, but not wild-type MEFs, strongly promoted invasion of 4T1 breast carcinoma cells in organotypic culture, and this was inhibited by small interfering RNA (siRNA)-mediated MRTF depletion (Figure 6E; Figure S5C). In sum, these data show that the TCFs generally inhibit MRTF-SRF signaling and that this reflects direct competition with the MRTFs for access to SRF, rather than the TCFs' ability to induce gene expression (Discussion). As a result, TCF-MRTF antagonism can contribute to control of “activated” fibroblast phenotypes, as seen in carcinoma-associated fibroblasts (Figure 6F; Discussion).

## Discussion

### Most TPA-Induced Gene Activation Is TCF-Dependent

The ERK-regulated TCF family of SRF partner proteins were first identified as regulators of the *Fos* gene (Buchwalter et al., 2004, Shaw et al., 1989), but the extent of their role in immediate-early gene expression and its significance for cell proliferation have been unclear. We found that TPA-induced genes in MEFs are enriched for gene hallmarks and GO terms associated with signal transduction, metabolism, transcription, and proliferation. Over 60% of TPA-induced gene expression in MEFs was TCF-dependent, and the proliferation of TCF-deficient MEFs was impaired. Previous studies have implicated the TCFs in control proliferation in response to adhesive and oncogenic stimuli (Vickers et al., 2004, Wozniak et al., 2012, Yang et al., 2012), and our results show that TCF-SRF signaling controls a gene expression program governing proliferation. We also found that TPA significantly downregulated many MRTF-SRF-controlled genes, consistent with the finding that TPA also downregulates Rho (Panayiotou et al., 2016).

### The Role of the TCFs in TPA-Induced Transcriptional Activation

We used an integrated SRF ChIP-seq/Hi-C approach to define a set of 763 TPA-induced TCF-SRF Direct target genes, whose TSSs are in close proximity to and/or physically linked to SRF binding sites. The Direct TCF-dependent gene signature provides a picture of the acute transcriptional response to ERK activation and, like the TPA-induced signature as a whole, it is significantly enriched in genes involved in signaling, transcription, and proliferation. Regulated expression of many Direct TCF-SRF target genes in TCF-deficient MEFs in TKO cells could be restored by re-expression of human Elk-1. TCF activity also promoted basal transcription of many genes in MEFs, whether TPA-inducible or not. This might reflect stochastic pulses of ERK activation that occur in many cell types (Aoki et al., 2013).

MRTF-SRF signaling regulates target genes involved in cytoskeletal dynamics (Esnault et al., 2014, Medjkane et al., 2009, Olson and Nordheim, 2010, Schratt et al., 2002), and, interestingly, numerous members of the Direct TCF-SRF-dependent gene set were defined previously as MRTF-SRF targets in serum-stimulated NIH 3T3 cells (Esnault et al., 2014). Thus, at a significant number of genes, both cofactor families can variably access SRF according to cellular context (Figure 6F). Our results imply that the relative levels of TCF and MRTF proteins in different biological contexts can affect the balance between proliferative and cytoskeletal gene expression programs (Figure 6F); this is discussed further below. The Yap-Taz proteins are also implicated in the control of both proliferative and cytoskeletal gene expression, acting at least in part through long-range TEAD/AP-1 elements (Dupont et al., 2011, Zanconato et al., 2015). Several AP-1 components are encoded by SRF target genes, providing a potential mechanism by which the two pathways might converge.

We found that TCF-SRF signaling contributes to TPA-induced *Myc* transcription and identified five distant potential regulatory SRF sites. *Myc* was first identified as a mitogen-inducible gene over 30 years ago (Greenberg and Ziff, 1984, Kelly et al., 1983), and its regulation remains poorly understood, but our findings identify a regulatory input for ERK signaling (Kerkhoff et al., 1998). The E box Myc binding consensus is enriched at putative regulatory sequences of genes that are indirectly TCF-responsive, consistent with *Myc* playing a role in the secondary response to growth factor stimulation. In spite of this, *Myc* cooperates with *Ras* in transformation, perhaps reflecting downregulation of ERK by chronic Ras activation.

Previous work has shown that the TCFs can regulate transcription independently of SRF (Boros et al., 2009a, Boros et al., 2009b, Buchwalter et al., 2005), but Elk-1 predominantly acted through SRF in our system. The ability of Elk-1 to restore TPA-inducible transcription in TKO MEFs required its transcriptional activation domain, indicating that Elk-1 acts by providing a primary signal input for activation. Nevertheless, Elk-1 failed to restore the TPA-induced transcriptional response at all TCF-dependent direct SRF targets; it also restored chromatin modifications at only a subset of TCF-dependent SRF target TSSs (C.E., F.G., S.H., A.S., N.M., R.T., G. Kelly, and P. East, unpublished data). Although it is likely that this reflects TCF-specific gene targeting, our results suggest that the strength of transcription activation by the different TCFs may also play a role. In a direct comparison, SAP-1 both activated transcription and restored proliferation more effectively than Elk-1, suggesting that it may restore a greater fraction of TCF-dependent gene expression. However, expression of mouse Elk-1 was also more effective than human Elk-1 in restoring proliferation (Mylona et al., 2016). Resolution of these issues will require direct comparative analysis of TCF recruitment with genomic targets.

### TCF Target Gene Identification by Integrated ChIP-Seq/Hi-C

We identified over 700 TCF-dependent direct SRF target genes by integrating ChIP-seq data from unstimulated MEFs with published MEF Hi-C datasets (Battulin et al., 2015, Minajigi et al., 2015), an approach used previously to identify NF-κB target TSSs (Jin et al., 2013). Although this approach uses the stringent criterion of direct TF-TSS physical interaction to identify candidate TF targets, because of limitations of the Hi-C data it is likely to under- rather than over-estimate the number of true targets. We note, however, that many SRF-interacting TSSs remained unaffected by TPA stimulation and must be somehow refractory to SRF-linked signals, as observed previously (Esnault et al., 2014). Thus, even with an additional functional constraint, establishing SRF-TSS linkage by Hi-C is not sufficient to predict whether a TSS is signal-regulated.

The integrated SRF ChiP-Seq/Hi-C approach also allowed us to define more than 1,000 TPA-inducible genes that, although TCF-dependent, are not direct TCF-SRF targets. Induction of these genes must either be dependent on the basal expression of Direct TCF-SRF target genes or arise as a secondary response to their acute activation. The putative regulatory sequences associated with Indirect TCF-SRF targets are significantly enriched for the binding motifs of transcription factors encoded by Direct TCF-SRF targets, supporting the notion that the IE response is a transcription cascade.

### The TCFs Antagonize MRTF-SRF Signaling

Our data show that TCF-MRTF antagonism is a general feature of SRF regulation, reflecting their competition for binding to SRF, to which their binding is mutually exclusive (Miralles et al., 2003, Wang et al., 2004, Zaromytidou et al., 2006; Figure 6F). In fibroblasts, TCF-MRTF competition at many SRF sites potentially influences the “activation” state by controlling contractility and pro-invasive behavior. Nevertheless, many SRF target genes appear to be coupled predominantly to one pathway or the other (Gineitis and Treisman, 2001, Sotiropoulos et al., 1999). This preference is likely determined by the quality of the SRF binding site. TCF binds preferentially to sites with well-defined Ets motifs (Treisman et al., 1992), whereas MRTF-SRF interaction is favored at sites that can be easily bent (Zaromytidou et al., 2006). Indirect MRTF-TCF antagonism can also occur, however, as in the control of ERK signaling by the MRTF target gene Mig6 (Descot et al., 2009).

Our data suggest a model in which the pro-proliferative effects of TCF-SRF signaling and increased contractility brought about by MRTF-SRF signaling are mutually antagonistic. In smooth muscle cells, PDGF stimulation induces exchange of the Elk-1 TCF for myocardin (Wang et al., 2004), indicating that this antagonism may itself be influenced by signaling. Although TCF DNA binding activity is not influenced by signal strength, nuclear MRTF levels in resting cells are controlled by cellular G-actin concentration (Miralles et al., 2003, Vartiainen et al., 2007). Thus, TCF-MRTF competition will also be affected by the basal level of Rho signaling, and factors affecting the degree to which a particular stimulus activates Rho- and ERK-dependent pathways will therefore also influence SRF transcriptional outputs (Figure 6F). It is tempting to speculate that it is TCF-MRTF competition that has provided the selective pressure to maintain the single SRF gene during metazoan evolution.

## Experimental Procedures

### Cells

Wild-type or Elk1^−/−^ Elk3^δ/δ^ Elk4^−/−^ mouse embryo fibroblasts (Costello et al., 2010) were immortalized by expression of SV40 large T. For stimulation with 50 ng/ml TPA, MEFs were maintained in 0.3% FCS. MEFs were reconstituted with wild-type human Elk-1 and SAP1 and Elk-1^nonA^ and Elk-1^ΔFW^ (Cruzalegui et al., 1999, Marais et al., 1993, Price et al., 1995) by retroviral transduction using standard procedures. Pools of MEFs expressing TCFs at similar levels were derived by fluorescence-activated cell sorting (FACS) for GFP marker expression. Fibroblast organotypic cultures and force-mediated matrix remodeling assays were as described previously (Calvo et al., 2013). High-throughput imaging was used to quantify actin stress fiber length using the Cellomics ArrayScan VTI and compartmental analysis (BioApplications; 3,000 cells/point).

### ChIP and RNA Analysis

ChIP and ChIP-seq have been described previously (Esnault et al., 2014, Miralles et al., 2003) with sequencing on the Hi-seq2500 (150-base read lengths). Antibodies used were as follows: SRF (sc-335, lot no. D3013, Santa Cruz Biotechnology) and MRTF-A (sc-21558, lot no. I1412, Santa Cruz); anti-mouse Elk-1, amino acids (aa) 309–429, was made in-house (Costello et al., 2010) and affinity-purified against recombinant human Elk-1^nonA^ (aa 309–429). qPCR gene expression analysis was by standard methods. For primers, see Supplemental Information.

### RNA-Seq

Samples were prepared using the GenElute mammalian total RNA miniprep kit (RTN350-1KT). DNA was removed by DNAase I and rRNA by the Ribo-zero rRNA removal kit (Epicenter). RNA-seq libraries were prepared from 1 μg RNA using the directional mRNA-seq Library Prep v1.0 protocol (Illumina). Libraries were subjected to 72-base single-end sequencing on the Hiseq analyzer, and trimmed to 50 bp. Raw and processed data are in the GEO: GSE75667.

RNA-seq data were aligned to the mm9 mouse genome using Burrows-Wheeler Aligner (BWA) (default settings). Reads within annotated RefSeq genes (“all reads”) and reads containing intronic sequences (“intronic reads”) were annotated using the bam file obtained by BWA as input. Normalization was against a set of invariant genes across samples, defined as those with differences in read counts within 1σ from the mean difference (μ_diff_), assuming a quasi-normal distribution of gene read counts. Differential gene expression analysis was performed with Deseq (comparing resting and TPA-induced, wild-type, and TKO MEF samples; adjusted p value (adj-p) ≤ 0.01; minimum change of 10%). The effects of knockout and reconstituted background were estimated by comparing the degree of gene induction under the different conditions and identifying genes that are similarly affected (Supplemental Information).

### ChIP-Seq Analysis

ChIP-seq reads were trimmed to 50 bp and aligned using BWA to the mm9 mouse genome (default settings). Raw and processed data are online in the GEO: GSE75667. Candidate SRF and Elk-1 peaks were identified by Model-based Analysis of ChIP-seq (MACS) (Zhang et al., 2008), retaining those called at p < 1E-4 in at least three pseudo-replicate samples. Those whose read distribution was significantly different from Elk-1 ChIP-seq, performed on TKO MEFs transduced with empty vector, were identified using Deseq (minimal increase 1.5-fold, p ≤ 0.05) and retained for further study. This approach has an effectively negligible false discovery rate (FDR). HOMER was used for motif discovery in sequences ± 100 bp from the ChIP-seq peaks or DHS peaks.

### Hi-C Analysis

This analysis used the HOMER Hi-C analysis pipeline (http://homer.salk.edu/homer/interactions). Reads from NCBI SRA: SRX554530 (Battulin et al., 2015), and GEO: GSM1648486 and GSM1696042 (Minajigi et al., 2015) were aligned to the mouse genome (Imakaev et al., 2012). A “universe” of ∼1.3 million significant interactions at 10-kb-resolution interactions was determined, taking into account linear genomic distance and sequencing depth (p ≤ 0.05 and *Z* score ≥ 2). Chromosomal regions exhibiting preferential interactions were identified by using the automated PCA analysis on Hi-C data in HOMER (runHiCpca.pl), retaining those with a PCA score >100. Interactions mapping at all RefSeq-annotated TSSs (∼130,000) were retrieved using the annotateInteractions.pl pipeline.

## Author Contributions

F.G. and C.E. designed, conducted, and interpreted experiments. F.G., S.H., and A.S. analyzed the genomic data and wrote scripts. N.M. performed the sequence analysis. R.T. conceived the project, suggested and interpreted experiments, and wrote the paper with F.G.

## Accession Numbers

The accession numbers for the raw sequencing files and the processed data reported in this paper are GEO: GSE75667.

## Supplemental Information

Document S1. Supplemental Experimental Procedures and Figures S1–S6

Table S1. Genome-wide Description of TPA-Induced Gene Changes and the Role of the TCFs, Related to Figure 1 Columns 1–6. Gene description: Official gene symbol; transcript ID; Genomic coordinates Chromosome, Strand, Start and Stop). Column 7. Gene activity: active (1) or inactive (0). Columns 8–10. Effects of 30 minute TPA stimulation, and dependence on TCF. TPA-induced (0 or 1) assessed by Deseq (see Materials and methods); TCF-dependence (0 or 1) assessed by regression method; TPA down-regulated genes (0 or 1) assessed by Deseq. Column 11. Changes in basal expression for each gene comparing WT MEF with TKO MEF – up- regulated (UP), down- regulated (DOWN) or not changing (ns) assessed by Deseq. Columns 12–16. Relationship between each gene and the 2577 SRF ChIP-seq peaks, including losest peak coordinate; distance to the closest peak; coordinates of remote SRF peaks interacting with the gene TSS in HI-C; total number of remote SRF peaks interacting with the gene TSS in HI-C; genes considered as Linked (by Hi-C) or Near (within 10kb of an SRF peak). Columns 17–23. Membership of the different gene sets described in Figure S3. Columns 23–26. Normalised total RNA readcounts in wildtype MEF and TKO MEF before and after TPA stimulation. Columns 27–30. Normalised intronic RNA read counts in wildtype MEF and TKO MEF before and after TPA stimulation.

Table S2. Overlap between Global Gene Expression Profiles in MEFs and Hallmark Gene Signatures or GO Terms from MsigDB, Related to Figure 1 Column 1. (A) Gene signatures: the 53 gene Signature ID from MSigDB, together with SRF, MRTF and TCF signatures. (B) GO terms from MsigDB. Columns 2–5. (A, B) Hypergeometrical test of each gene set (TPA-induced, TPA-downregulated, Baseline-enhanced in TKO, and Baseline-impaired in TKO) against each gene signature. The Bonferroni adjusted p value is shown, and the number of genes in both sets. Columns 6–9. (A, B) Gene name IDs for genes in both sets.

Table S3. ChIP-Seq Analysis, Related to Figure 3 and Figures S3 and S4 Columns 1–5. Peak coordinates for each of the 2577 SRF peaks and 336 Elk-1 peaks: Peak ID followed by the coordinate of the peak called by MACS and filtered using Deseq (Chromosome, Start, Stop, Peak summit coordinate). Columns 6–11. Peak information (1 or 0): called by SRF ChIP-seq (n=2577); enhanced by presence of the TCFs; called by Elk-1 ChIP-seq in reconstituted TKO MEFs (n=336); w called in both SRF and Elk-1 ChIP-seq conditions; whether involved in HI-C interactions to TSSs; coincidence with DNA’se I HS. Columns 12–20. Description of closest gene to the peak: Gene name; Activity; whether induced by TPA; whether TPA induction is TCF-dependent; whether Elk-1 expression in TKO MEFs is sufficient to restore regulation; TPA-downregulated; effect of TCF inactivation on basal expression; distance from peak to TSS; location of the peak (intergenic, intron, other gene feature...). Columns 21–26. Averaged normalised read counts across the various conditions: SRF ChIP-seq in wildtype and TKO MEFs; Elk-1 ChIP-seq in TKO MEFs reconstituted with Vector (negative control used in Deseq), wildtype Elk-1, Elk-1 ΔFW, Elk-1 nonA. Columns 27–42. Normalised read counts in each condition.

Table S4. Overlap between SRF-Linked Gene Sets and Hallmark Gene Signatures or GO Terms from MsigDB, Related to Figure 3 and Figure S3 Column 1. (A) Gene signatures: the 53 gene Signature ID from MSigDB, together with SRF, MRTF and TCF signatures. (B) GO terms from MSigDB. Columns 2–10. (A,B) Hypergeometrical test against each gene signature for each set of genes whose TSS is in close proximity to or interacting with SRF in Hi-C unless otherwise stated. The Bonferroni adjusted p value is shown, and the number of present in both gene sets. Gene sets analysed are: TPA-induced, linked to SRF; TPA-induced, Direct TCF-dependent targets; TPA-induced, Indirect TCF-dependent targets (these are not SRF-linked); TPA-downregulated; Baseline-elevated in TKO MEFs; Baseline-elevated in TKO MEFs. Columns 11–19. (A,B) Gene name IDs for genes in both sets.

Table S5. Reconstitution of TKO MEFs with Elk-1 Derivatives, Related to Figure 4 and Figure S4 This table is as Table S1 but focuses on TPA-regulated genes whose TSS close to and/or linked by Hi-C to SRF/Elk-1 or Elk-1 solo peaks. Columns 1–6. Gene description: Official gene symbol; transcript ID; Genomic coordinates (Chromosome, Strand, Start and Stop). Column 7. Gene activity: active (1) or inactive (0). Columns 8–10. Effects of 30 minute TPA stimulation, and dependence on TCF. TPA-induced (0 or 1) assessed by Deseq (see Materials and methods); TCF dependent (0 or 1) assessed by regression method; regulation restored by Elk-1 expression (0 or 1) assessed by regression method. Columns 11–15. Relationship between each gene and the 2577 SRF peaks: closest peak coordinate; distance from TSS to the closest peak; remote SRF peak coordinates interacting with the gene TSS in Hi-C; number of remote SRF peaks interacting with the gene TSS in Hi-C; gene considered as SRF-linked (ie interacting by Hi-C or within 10kb of an SRF peak). Columns 16–20. Relationship between each gene and the 251 SRF/Elk-1 peaks: closest peak coordinate; distance from TSS to the closest peak; remote SRF/Elk-1 peaks coordinates interacting with the gene TSS in Hi-C; number of remote SRF/Elk-1 peaks interacting with the gene TSSin Hi-C; gene considered as SRF/Elk-1-linked (ie interacting by Hi-C or within 10kb of an SRF/Elk-1 peak). Columns 21–25. Relationship between each gene and the 85 Elk-1 solo peaks. Closest peak coordinate; distance from TSS to the closest peak; remote Elk-1 solo peak coordinates interacting with the gene TSS in Hi-C; number of remote Elk-1 solo peaks interacting with the gene TSS in Hi-C; gene considered as SRF-linked (ie interacting by Hi-C or within 10kb of an Elk-1 solo peak). Columns 26–41. Normalised total read counts and normalised read counts matching intronic features.

Table S6. ERK Signalling to Transcription Factors Encoded by Direct TCF-SRF Target Genes, Related to Figure 4 The 54 TFs directly controlled by the TCFs are listed, with references and brief summary of the consequences of ERK signalling.

Document S2. Article plus Supplemental Information
