# Supplementary material for: SRF Co-factors Control the Balance between Cell Proliferation and Contractility
Source: Mol Cell. 2016 Dec 15;64(6):1048–61. doi: 10.1016/j.molcel.2016.10.016 (PMC5179500; doi:10.1016/j.molcel.2016.10.016)
Supplement: Document S1. Supplemental Experimental Procedures and Figures S1–S6 [file mmc1.pdf]

Molecular Cell, Volume *64*

Supplemental Information

SRF Co-factors Control the Balance

between Cell Proliferation and Contractility

Francesco Gualdrini, Cyril Esnault, Stuart Horswell, Aengus Stewart, Nik Matthews, and Richard Treisman

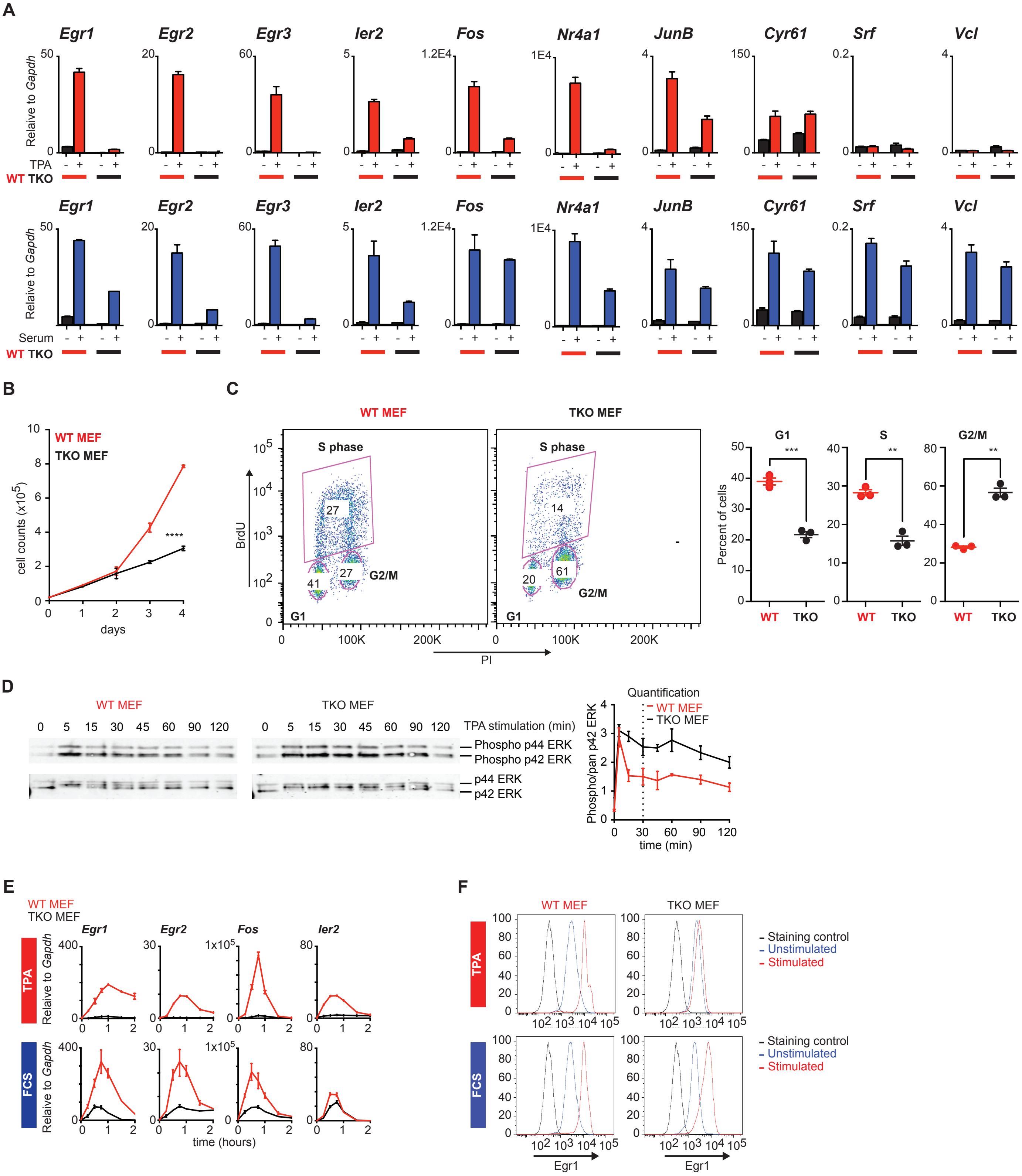
A
Egr1
Egr2
Egr3
Ier2
Fos
Nr4a1
JunB
Cyr61
Srf
Vcl
Relaive to Gapdh
TPA
Serum
WT TKO
B
WT MEF
TKO MEF
cell counts (x10^5)
days
****
C
WT MEF
TKO MEF
S phase
G2/M
G1
BrdU
PI
Percent of cells
G1
S
G2/M
WT
TKO
D
WT MEF
TKO MEF
TPA stimulation (min)
Phospho p44 ERK
Phospho p42 ERK
p44 ERK
p42 ERK
Quantification
Phospho/pan p42 ERK
time (min)
E
WT MEF
TKO MEF
TPA
FCS
time (hours)
F
Staining control
Unstimulated
Stimulated
Egr1

Figure S1

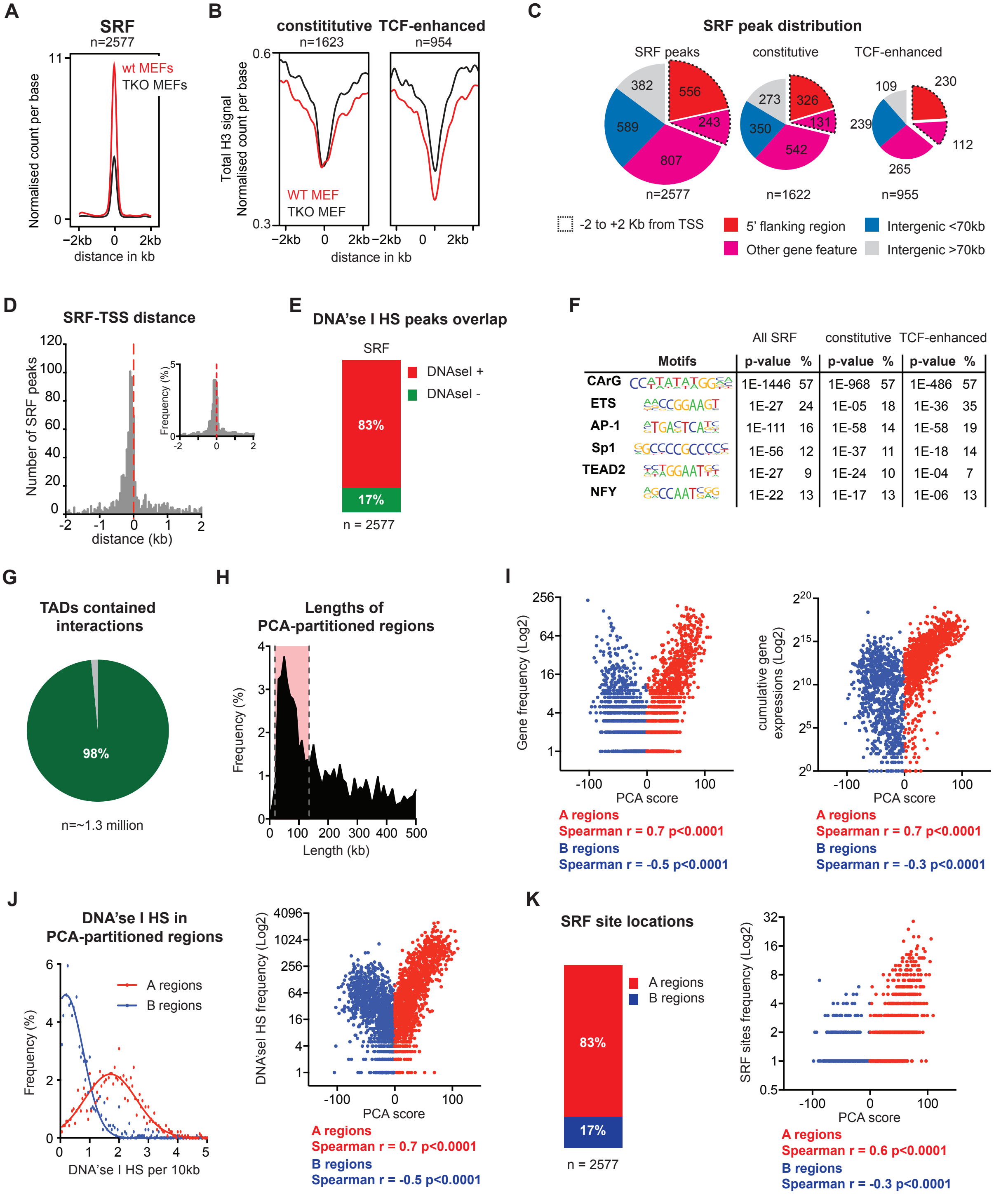

Motifs	All SRF p-value	%	constitutive p-value	%	TCF-enhanced p-value	%
CArG	1E-1446	57	1E-968	57	1E-486	57
ETS	1E-27	24	1E-05	18	1E-36	35
AP-1	1E-111	16	1E-58	14	1E-58	19
Sp1	1E-56	12	1E-37	11	1E-18	14
TEAD2	1E-27	9	1E-24	10	1E-04	7
NFY	1E-22	13	1E-17	13	1E-06	13

Figure S2

A

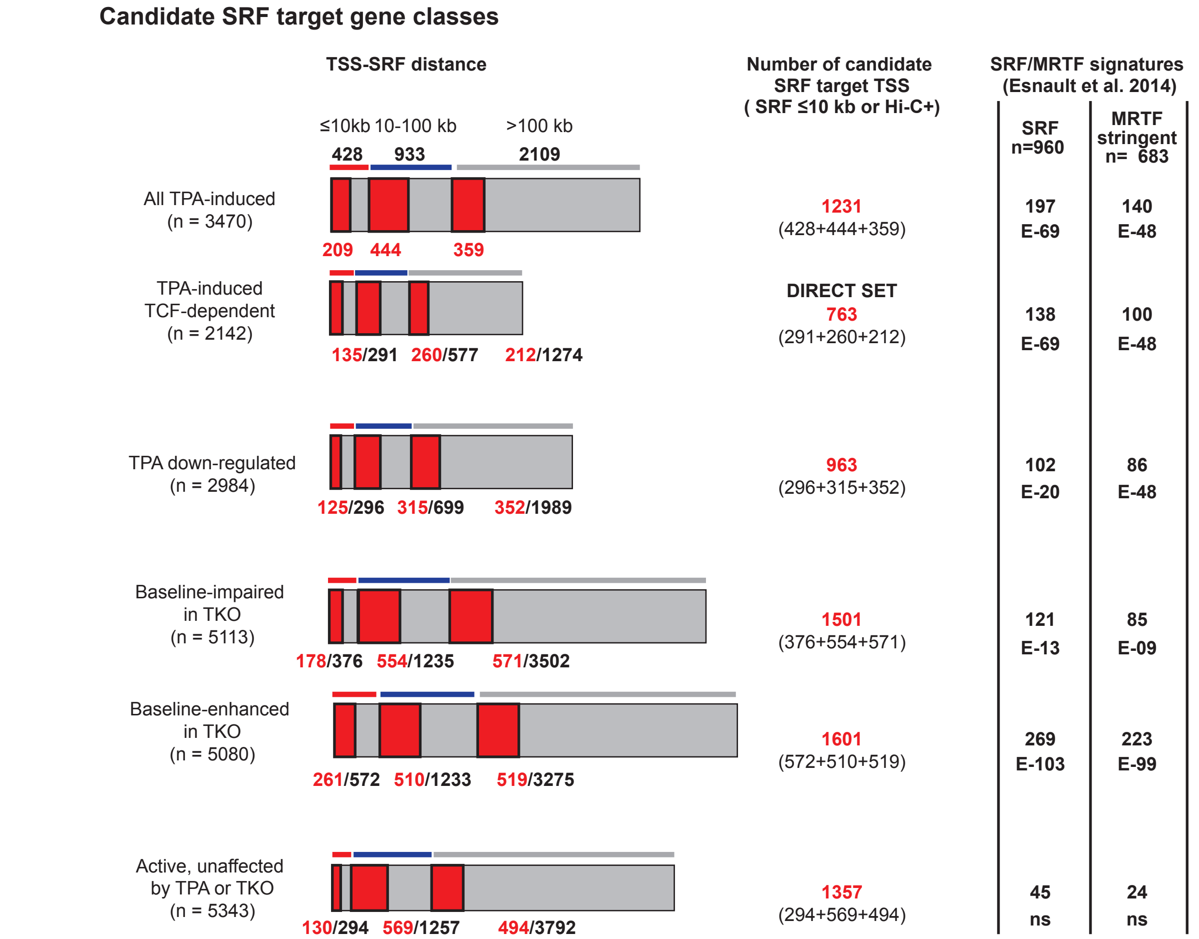

B

Relationship between TSS-associated gene function and SRF binding sites

	Relation of TSS-associated gene function to SRF ChIP-seq peaks in wildtype MEFs		Relation of TSS-associated gene function to SRF/Elk-1 ChIP-seq peaks in reconstituted TKO MEFs	
	Candidate SRF target TSS (≤10 kb from SRF or in Hi-C contact)	Number of SRF peaks associated with candidate target TSS (specific to gene class)	Candidate SRF/Elk-1 target TSS (≤10 kb from SRF/Elk-1 or in Hi-C contact)	Number of SRF/Elk-1 peaks associated with target TSS (specific to gene class)
All TPA-induced (n = 3470)	1231	964	239	119
TPA-induced TCF-dependent (n = 2142)	763	721	151 (94 rescued by Elk-1 o/ex)	89
TPA down-regulated (n = 2984)	963	1113 (28)	146	101 (0)
Baseline-impaired in TKO (n = 5113)	1501	1133 (113)	218	124 (13)
Baseline-enhanced in TKO (n = 5080)	1601	1275 (134)	252	136 (11)
Active, unaffected by TPA or TKO (n = 5343)	1357	953 (130)	222	102 (6)
		Unassigned: 585<br>Total: 2577		Unassigned: 47<br>Total: 251

Figure S3

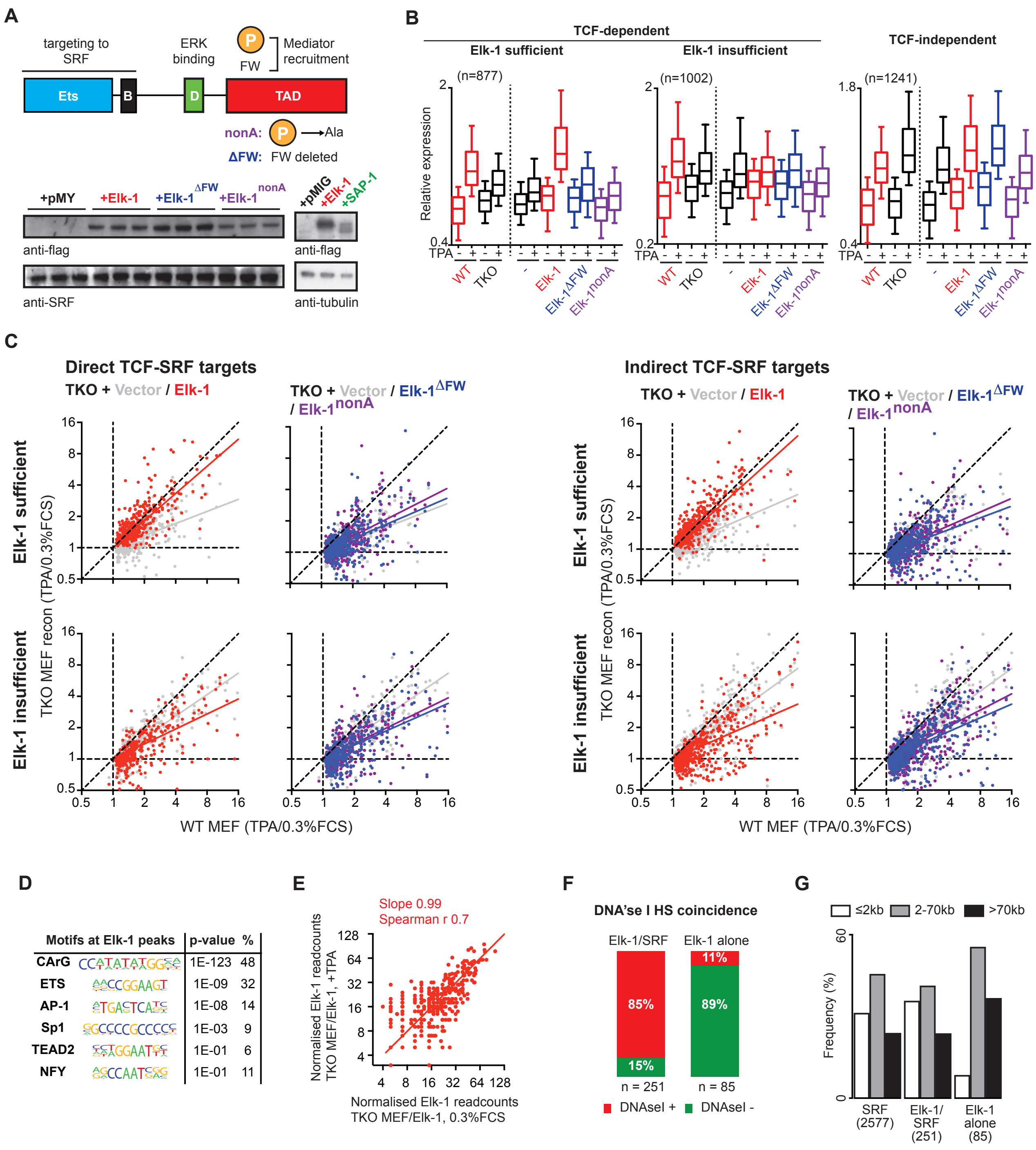
A
targeting to SRF
ERK binding
P
FW
Mediator recruitment
Ets
B
D
TAD
nonA:
P
Ala
ΔFW: FW deleted
+pMY
+Elk-1
+Elk-1ΔFW
+Elk-1nonA
+pMIG
+Elk-1
+SAP-1
anti-flag
anti-SRF
anti-flag
anti-tubulin
B
TCF-dependent
Elk-1 sufficient
Elk-1 insufficient
TCF-independent
(n=877)
(n=1002)
(n=1241)
Relative expression
TPA
WT
TKO
-
Elk-1
Elk-1ΔFW
Elk-1nonA
C
Direct TCF-SRF targets
Indirect TCF-SRF targets
TKO + Vector / Elk-1
TKO + Vector / Elk-1ΔFW / Elk-1nonA
Elk-1 sufficient
Elk-1 insufficient
TKO MEF recon (TPA/0.3%FCS)
WT MEF (TPA/0.3%FCS)
D
Motifs at Elk-1 peaks | p-value | %
CArG | 1E-123 | 48
ETS | 1E-09 | 32
AP-1 | 1E-08 | 14
Sp1 | 1E-03 | 9
TEAD2 | 1E-01 | 6
NFY | 1E-01 | 11
E
Slope 0.99
Spearman r 0.7
Normalised Elk-1 readcounts TKO MEF/Elk-1, +TPA
Normalised Elk-1 readcounts TKO MEF/Elk-1, 0.3%FCS
F
DNA'se I HS coincidence
Elk-1/SRF
Elk-1 alone
85%
15%
11%
89%
n = 251
n = 85
DNAseI +
DNAseI -
G
≤2kb
2-70kb
>70kb
Frequency (%)
SRF (2577)
Elk-1/SRF (251)
Elk-1 alone (85)

Figure S4

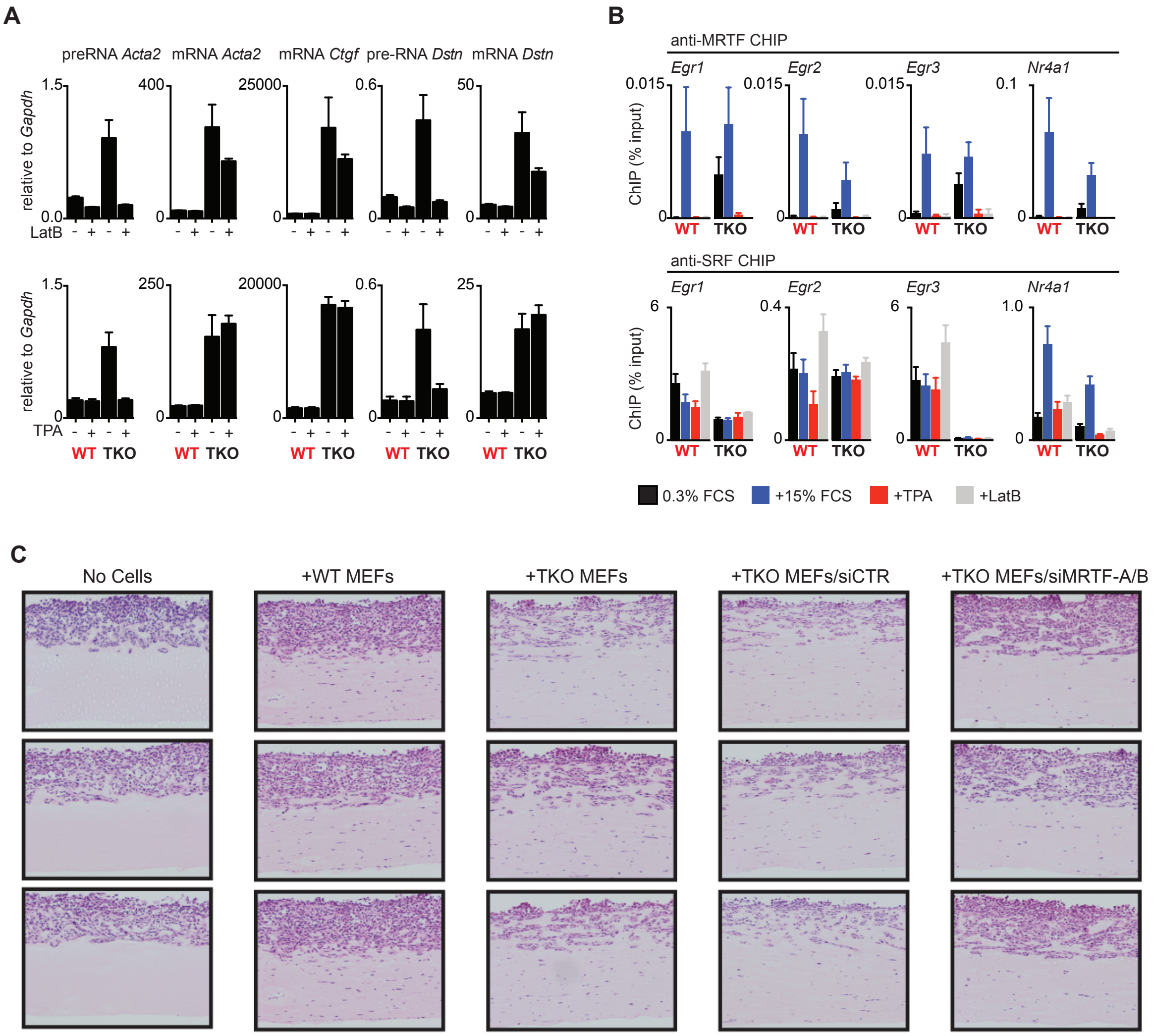
A
preRNA Acta2
mRNA Acta2
mRNA Ctgf
pre-RNA Dstn
mRNA Dstn
relative to Gapdh
LatB
TPA
WT TKO
B
anti-MRTF CHIP
anti-SRF CHIP
Egr1
Egr2
Egr3
Nr4a1
ChIP (% input)
0.3% FCS
+15% FCS
+TPA
+LatB
C
No Cells
+WT MEFs
+TKO MEFs
+TKO MEFs/siCTR
+TKO MEFs/siMRTF-A/B

Figure S5

SUPPLEMENTAL FIGURE LEGENDS

Figure S1| (related to Figure 1) TCF inactivation impairs IE gene induction and proliferation

(A) qRT-PCR analysis of IE gene transcription, normalised to GAPDH, in wildtype and TKO MEFs stimulated with TPA (red) or serum (blue). Data are means ± SEM, n=3.

(B) Proliferation is impaired in TKO MEFs. Cells were counted over a 4 day period. Data are means ± SEM, n=3 (****, $p<0.0001$, two-way ANOVA).

(C) TKO MEFs exhibit a prolonged G2/M phase. DNA content (propidium iodide staining) and BrdU incorporation (FITC staining) are plotted, with quantitation at right. ***, $p<0.001$; **, $p<0.01$; unpaired t test with Welch's correction.

(D) Elevated ERK activation persists following TPA stimulation of TKO MEFs, as assessed by immunoblotting, with quantation at right (mean ± SEM, n = 3).

(E) Normal kinetics but reduced magnitude of serum induction of IE genes in TKO MEFs. qRT-PCR analysis as in (A).

(F) Defective Egr1 gene induction is due to reduced response in all cells rather than lack of response in part of the population. FACS analysis of Egr1 protein accumulation following 1h TPA or serum stimulation of wildtype or TKO MEFs.

Figure S2 | (related to Figure 2) Integration of SRF ChIP-seq analysis of SRF binding in wildtype and TKO MEFs with MEF Hi-C data

(A) Metaprofiles of the 2577 SRF binding sites in wildtype (red) and TKO (black) MEFs.

(B) Metaprofiles of total H3 occupancy at either constitutive or TCF-enhanced SRF binding sites in wildtype and TKO MEFs.

(C) Distribution of SRF and Elk-1 peaks relative to Refseq annotated gene TSS. Dotted border denotes sites within ± 2Kb of the TSS.

(D) Distribution of the SRF sites around the TSS (inset: relative frequency).

(E) Most SRF sites coincide with DNA'se I HS sites. DNA'se I HS data is from GSM1003831, GSM1014199.

(F) SRF-associated transcription factor binding motifs within 100 bp of SRF peak summits

(G) Proportion of identified Hi-C interactions (~1.3 million total interactions identified at resolution of 10 kb resolution) contained within previously described TADs.

(H) Frequency distribution of the lengths of the regions defined by PCA analysis. Dotted grey limits display the theoretical ±1σ from the mean assuming a log-Gaussian distribution.

(I) Correlation of gene frequency and cumulative gene activity and PCA score, evaluated separately for A and B regions.

(J) Left, distribution of DNA’se I HS peak densities per 10kb in the A and B regions defined by PCA analysis. Right, correlation between DHS frequency per re region and PCA score, evaluated separately for A and B regions.

(K) Left, proportion of SRF ChIP-seq peaks contained in the A or B regions defined by PCA analysis; right, correlation of SRF site frequency per region, evaluated separately for A and B regions.

Figure S3 | (related to Figure 3, Tables S3, S4) Definition of potential SRF target genes

(A) Classes of potential SRF target gene are shown at left. Each grey block is scaled to the number of TSS in each class, and divided according to distance to the closest SRF site (<10kb, 10-100kb, >100kb). Red shading indicates that a TSS displays linkage to an SRF site in Hi-C. Not all interactions within 10kb of a TSS are detectable by our Hi-C analysis for technical reasons, and so potential SRF target genes are therefore defined as all those whose TSS are within 10kb of an SRF site, or that interact with one at any distance as judged by Hi-C. The relation between each target gene class and our previously defined SRF and MRTF signatures is shown at the right (hypergeometrical p-value and number of genes). The association of TPA-downregulated genes with SRF suggests a model in which these genes are controlled through MRTF and respond to downregulation of Rho-actin signalling by TPA (Panayiotou et al., 2016).

(B) Relationships between SRF binding sites and their neighbouring or interacting TSS. Note that 75-80% of SRF peaks can be associated with active genes. Left, all SRF sites; right, SRF/Elk-1 binding sites in TKO MEFs reconstituted with Elk-1. Gene classes are displayed as in (A), with the total number of linked SRF sites associated with those genes at the right. For classes of gene other than inducible ones, the number of sites associated only with genes of that class is indicated in brackets. Overall data can be retrieved by combining information contained in Tables S1, S4 and S7. The GRanges tools in R were used to assess both distances between TSSs and peaks and the match between peaks and hi-C anchor points. Of 1129 SRF sites with 10kb of a TSS, 625 were in Hi-C contact with other TSS, of which 299 were TPA-inducible and 174 TCF-dependent. In the Elk-1 reconstituted TKO MEFs, a total of 168 unique SRF/Elk-1 peaks out of the 251 total SRF/Elk-1 peaks were associated with genes whose basal level or induction was TCF-dependent. This number is likely an underestimate since some TCF-independent TSS may be false-negatives. It represents 67% of the total 251 SRF/Elk-1 peaks, and 82% of those peaks associated with active genes. As expected, no SRF/Elk-1 peaks were exclusively associated with TPA-downregulation.

Figure S4 | (related to Figure 4, Tables S3, S5) Restoration of TPA regulation in TKO MEFs by Elk-1 expression

(A) Expression of TCF derivatives in MEFs. Top, TCF functional domains. Mutant Elk-1^{nonA} contains alanine substitutions at nine conserved S/T-P

phosphoacceptor motifs, and mutant Elk-1$^{\Delta FW}$ deletes a conserved FW required for Mediator recruitment. Bottom, expression of TCFs in reconstituted TKO MEFs. Expression of Elk-1 mutants (lysates from three separate samples for each) and the Elk-1 and SAP-1 TCFs was analysed by immunoblot for the Flag epitope.

(B) Box plot summarising the effect of Elk-1 expression on signal-induced changes at TCF-dependent genes, partitioned according to whether Elk-1 expression restores regulation or not, and at TCF-independent genes.

(C) Scatter plots showing the relationship between gene induction in wildtype MEFs, and in TKO MEFs reconstitution with vector alone (grey), wildtype Elk-1 (red), Elk-1^{FW} (blue) or Elk-1^{nonA} (purple). Genes are partitioned according to whether wildtype Elk-1 expression is sufficient to rescue regulation.

(D) Elk-1 associated sequence motifs within 100 bp of Elk-1 peak summits.

(E) TPA stimulation does not affect Elk-1 DNA binding. Correlation of normalised Elk-1 ChIP-seq signals in resting and TPA stimulated cells.

(E) Relation between Elk-1 ChIP-seq signals before and after stimulation.

(F) Proportion of the Elk-1/SRF or Elk-1 coincident with 1 DNA’se I HS peak.

(G) Frequency distribution of the distance between SRF, Elk-1/SRF or Elk-1 solo ChIP-seq peaks and their closest TSS.

Figure S5 | (related to Figure 6) The TCFs antagonise MRTF-SRF dependent gene expression

(A) Elevated levels of MRTF-SRF target gene expression in TKO MEFs are sensitive to MRTF inhibition by Latrunculin B. RNA from cycling cells of cells were treated with LatB for 5 minutes was analysed by qRT-PCR. Error bars, SEM; n=4.

(B) Quantitative ChIP analysis of MRTF-A and SRF binding at regulatory elements the direct TCF target genes *Egr1*, *Egr2*, *Egr3* and *Nr4a1* in wildtype and TKO MEFs. Assays were from cells in resting conditions (0.3% FCS; black), after 30' serum or TPA stimulation (blue and black respectively), or after 5 min treatment with Latrunculin-B (grey). Error bars, SEM; n=4.

(C) TKO MEFs exhibit enhanced pro-invasive activity. Replicate assays showing the invasion of 4T1 breast carcinoma cells were allowed to invade a matrix composed of collagen I and Matrigel, that contained no fibroblasts, wildtype or TKO MEFs, or TKO MEFs treated with control or MRTF-A/B siRNAs as indicated. Images are from each of 3 independent invasion assays.

TABLE LEGENDS

Table S1 | (related to Figure 1) Genome wide description of TPA-induced gene changes and the role of the TCFs.

Columns 1-6. Gene description: Official gene symbol; transcript ID; Genomic coordinates (Chromosome, Strand, Start and Stop).

Column 7. Gene activity: active (1) or inactive (0).

Columns 8-10. Effects of 30 minute TPA stimulation, and dependence on TCF. TPA-induced (0 or 1) assessed by Deseq (see Materials and methods); TCF-dependence (0 or 1) assessed by regression method; TPA down-regulated genes (0 or 1) assessed by Deseq.

Column 11. Changes in basal expression for each gene comparing WT MEF with TKO MEF – up- regulated (UP), down- regulated (DOWN) or not changing (ns) assessed by Deseq.

Column 12-16. Relationship between each gene and the 2577 SRF ChIP-seq peaks, including losest peak coordinate; distance to the closest peak; coordinates of remote SRF peaks interacting with the gene TSS in HI-C; total number of remote SRF peaks interacting with the gene TSS in HI-C; genes considered as Linked (by Hi-C) or Near (within 10kb of an SRF peak).

Columns 17-23. Membership of the different gene sets described in Figure S3.

Column 23-26. Normalised total RNA readcounts in wildtype MEF and TKO MEF before and after TPA stimulation.

Column 27-30. Normalised intronic RNA read counts in wildtype MEF and TKO MEF before and after TPA stimulation.

Table S2 | (related to Figure 1) Overlap between global gene expression profiles in MEFs and (A) Hallmark gene signatures or (B) GO terms from MsigDB.

Column 1. (A) Gene signatures: the 53 gene Signature ID from MSigDB, together with SRF, MRTF and TCF signatures. (B) GO terms from MsigDB.

Columns 2-5. (A,B) Hypergeometrical test of each gene set (TPA-induced, TPA-downregulated, Baseline-enhanced in TKO, and Baseline-impaired in TKO) against each gene signature. The Bonferroni adjusted p-value is shown, and the number of genes in both sets.

Columns 6-9. (A,B) Gene name IDs for genes in both sets.

Table S3 | (related to Figure 3, Figure S3, S4) ChIP-seq analysis.

Columns 1-5. Peak coordinates for each of the 2577 SRF peaks and 336 Elk-1 peaks: Peak ID followed by the coordinate of the peak called by MACS and filtered using Deseq (Chromosome, Start, Stop, Peak summit coordinate).

Column 6-11. Peak information (1 or 0): called by SRF ChIP-seq (n=2577); enhanced by presence of the TCFs; called by Elk-1 ChIP-seq in reconstituted

TKO MEFs (n=336); w called in both SRF and Elk-1 ChIP-seq conditions; whether involved in HI-C interactions to TSSs; coincidence with DNA'se I HS.

Columns 12-20. Description of closest gene to the peak: Gene name; Activity; whether induced by TPA; whether TPA induction is TCF-dependent; whether Elk-1 expression in TKO MEFs is sufficient to restore regulation; TPA-downregulated; effect of TCF inactivation on basal expression; distance from peak to TSS; location of the peak (intergenic, intron, other gene feature...).

Column 21-26. Averaged normalised read counts across the various conditions: SRF ChIP-seq in wildtype and TKO MEFs; Elk-1 ChIP-seq in TKO MEFs reconstituted with Vector (negative control used in Deseq), wildtype Elk-1, Elk-1$^{\Delta FW}$, Elk-1^{nonA}.

Column 27-42. Normalised read counts in each condition.

Table S4 | (related to Figure 3, Figure S3) Overlap between SRF-linked gene sets and (A) Hallmark gene signatures or (B) GO terms from MsigDB.

Column 1. (A) Gene signatures: the 53 gene Signature ID from MSigDB, together with SRF, MRTF and TCF signatures. (B) GO terms from MSigDB.

Columns 2-10. (A,B) Hypergeometrical test against each gene signature for each set of genes whose TSS is in close proximity to or interacting with SRF in Hi-C unless otherwise stated. The Bonferroni adjusted p-value is shown, and the number of present in both gene sets. Gene sets analysed are: TPA-induced, linked to SRF; TPA-induced, Direct TCF-dependent targets; TPA-

induced, Indirect TCF-dependent targets (these are not SRF-linked); TPA-downregulated; Baseline-elevated in TKO MEFs; Baseline-elevated in TKO MEFs.

Columns 11-19. (A,B) Gene name IDs for genes in both sets.

Table S5 | (related to Figure 4, Figure S4) Reconstitution of TKO MEFs with Elk-1 derivatives.

This table is as Table S1 but focusses on TPA-regulated genes whose TSS close to and/or linked by Hi-C to SRF/Elk-1 or Elk-1 solo peaks.

Columns 1-6. Gene description: Official gene symbol; transcript ID; Genomic coordinates (Chromosome, Strand, Start and Stop).

Column 7. Gene activity: active (1) or inactive (0).

Columns 8-10. Effects of 30 minute TPA stimulation, and dependence on TCF. TPA-induced (0 or 1) assessed by Deseq (see Materials and methods);; TCF dependent (0 or 1) assessed by regression method; regulation restored by Elk-1 expression (0 or 1) assessed by regression method.

Columns 11-15. Relationship between each gene and the 2577 SRF peaks: closest peak coordinate; distance from TSS to the closest peak; remote SRF peak coordinates interacting with the gene TSS in Hi-C; number of remote SRF peaks interacting with the gene TSS in Hi-C; gene considered as SRF-linked (ie interacting by Hi-C or within 10kb of an SRF peak).

Column 16-20. Relationship between each gene and the 251 SRF/Elk-1 peaks: closest peak coordinate; distance from TSS to the closest peak; remote SRF/Elk-1 peaks coordinates interacting with the gene TSS in Hi-C; number of remote SRF/Elk-1 peaks interacting with the gene TSSin Hi-C; gene considered as SRF/Elk-1-linked (ie interacting by Hi-C or within 10kb of an SRF/Elk-1 peak).

Column 21-25. Relationship between each gene and the 85 Elk-1 solo peaks. Closest peak coordinate; distance from TSS to the closest peak; remote Elk-1 solo peak coordinates interacting with the gene TSS in Hi-C; number of remote Elk-1 solo peaks interacting with the gene TSS in Hi-C; gene considered as SRF-linked (ie interacting by Hi-C or within 10kb of an Elk-1 solo peak).

Column 26-41. Normalised total read counts and normalised read counts matching intronic features.

Table S6 | (related to Figure 4) ERK signalling to transcription factors encoded by Direct TCF-SRF target genes

The 54 TFs directly controlled by the TCFs are listed, with references and brief summary of the consequences of ERK signalling.

SUPPLEMENTAL METHODS

Cells

Mouse Embryonic fibroblasts (MEFs) were derived from wildtype mice (wildtype) or mice lacking the Elk-1, Net and SAP-1 TCFs (Elk1$^{-/-}$ Elk3$^{\delta/\delta}$ Elk4$^{-/-}$; TKO MEFs) and immortalized by infection with retrovirus expressing SV40 large T protein (Costello et al., 2010). MEFs were cultured in DMEM (Gibco, Invitrogen) supplemented with 10% FCS at 37°C and 10% CO_2. Cells were maintained in 0.3% FCS for 48 h, then stimulated with 12-O-Tetradecanoylphorbol-13-acetate (Sigma, TPA, 50ng/ml) for 30 minutes or as indicated in the Figure legends.

TKO MEF reconstitution using retroviral infection

TKO cells were reconstituted with human Elk-1 variants using retrovirus infection. Elk-1 wild type, Elk-1^{nonA} and Elk-1$^{\Delta FW}$ sequences (Cruzalegui et al., 1999; Marais et al., 1993; Price et al., 1995) were expressed using the retroviral vector pMYs (MSCV-(IRES)-EGFP, Cell Biolabs RTV-021); for comparative analysis, Elk-1 and SAP-1 were introduced using the pMIG (MSCV-IRES-GFP, Addgene 9044). GP2-293 cells were transfected with Elk-1 plasmids and pCMV-VSVG, using Fugene. Viral supernatants recovered 2-3 days later were used to infect Phoenix cells by spin infection. Viral supernatants were used to infect MEFs, which were sorted by FACS into

pools expressing GFP at low, medium and high level. Pools expressing similar amounts of Elk-1 derivatives were selected for further study.

qRT-PCR RNA analysis

For standard Q-PCR based gene expression analysis, cDNA synthesis used the SuperScript III first strand synthesis system with random hexamer primers (Invitrogen). SYBR Green-based real-time PCR (Invitrogen) was performed using dilutions of genomic DNA solution for calibration. The expression of each target was expressed relative to Gapdh.

The primer pairs used were : Gapdh: 5'- TCTTGTGCAGTGCCAGCCT-3', 5'-CAATATGGCCAAATCCGTTCA-3'; ACTA2: precursor 5'-CCAGAAGCAATGCGTCCACT-3', 5'-TGAGGTAGTTGCCTGCTCTC-3'; ACTA2: mRNA 5'-CTGTCAGGAACCCTGAGACGC-3', 5'-GGCTGTGCTGTCTTCCTCTT-3'; Ctgf: mRNA 5'-GGAGGAAAACATTAAGAAGGGCAA-3', 5'-AACTTGACAGGCTTGGCGAT-3'; Dstn: pre-RNA 5'-ATAGCAACTGGCTTGCAGGT-3', 5'-ACAGCTAAGCATGGTCCGTT-3'; Dstn: mRNA 5'-CGAACATGGCCTCAGGAGTT-'3, 5'-GGTGTGGAACATTTCCGAACTT-3'; Egr1: 5'-ATTGATGTCTCCGCTGCAGATC-3', 5'-TCAGCAGCATCATCTCCTCCA-3'; Egr2: 5'-GAGCAAATGATGACCGCCAA-3', 5'- TGTCAGGCAGCTGGTGCATAA-3'; Egr3: 5'-AGCCCAATCCGGAACTCTCTT-3', 5'-GGAAGGAGAGTCGAAAGCGAA-3';, Ier2: 5'-CCTGCGGTTCCTTTGTCCTTA-3', 5'-TCACTTTGGTTTCCGACATGC-3' , Per1: 5'-

AATAAGGCAGAGAGCGTGGTGTC-3’, 5’-TCTTGTCTCCCACATGGACGA-3’ , Junb: 5’-CCCTTCTCCCCTCCCTGTTA-3’, 5’-GTTTACATGGCCCCCTTCCA-3’ , Fos: 5’-TTCCTACTACCATTCCCCAGCC-3’, 5’-GATCTGCGCAAAAGTCCTGTGT-3’, Zfp36: 5’-CCAGGAGGCCAGATCTCTCT-3’, 5’-CACCTGTCTAGTGCCCTTCG-3’, Dnajb1: 5’-CCCGCTATAGTTCCTGTGGC-3’, 5’-TTCGGTTTCCCATGTGGAGG-3’, Arl5b: 5’-TGCACCTTCACGTTGTCTGA-3’, 5’-AGAAACCCGTGCACCTGAAT-3’, Pitpna: 5’-GGCAGCAGAGGAAAAGGGAT-3’, 5’-CCCTGTCATCCAGTCCCCTA-3’, Mob3a: 5’-TCTGCCCCATTGTAAAGCCA-3’, 5’-CTTGTCACTCCCTCGGTAGC-3’, Pramef8: 5’-TGTCTAGCTAGTGTTGCCCAC-3’, 5’-ACCAGCCCAAGCTTAACGAG-3’, Tada3: 5’-TGCCTCAGTCTATTTCCCCA-3’, 5’-GGTAATTCTCGATGCCCCCA-3’, Arhgap1 5’-CACTGTTGAGGGGGTTGACT-3’, 5’-CATAGAACCCAGACGCAGGA-3’, Arpc4: 5’-TACCTAGCTCTGGCACTGGT-3’, 5’-ATCCTGGCATGCACAACAGA-3’, Ier3: 5’-CCTCGAGTGGTGAGTATCGC-3’, 5’-CGTCAGATCGAAGGTCCCTG-3’, Klf10: 5’-AAATCCCTTGCAAGACCCCG-3’, 5’-CCCCTAAATAAGCCACGCCT-3’, Cxcl1: 5’-GCCACACTCAAGAATGGTCG-3’, 5’-GGGGTCATATGCCAGTACTCC-3’.

Total RNA preparation, library assembly and sequencing

Samples were prepared using GenElute Mammalian Total RNA Miniprep Kit (RTN350-1KT) and DNA contaminants were removed by DNA'se I treatment. The Ribo-zero rRNA removal kit (Epicentre) was used to remove ribosomal rRNA. RNA-seq libraries were prepared from at least 1µg RNA, using the directional mRNA-Seq Library Prep v1.0 Protocol (Illumina) with minor adjustments. To reduce volume of the PCR reactions, the Kapa HiFi HotStart ready mix, was substituted with the Illumina kit Phusion enzyme, the Agencourt AMPure XP beads being adjusted accordingly. The Illumina protocol PCR cycle was changed to match the quantity of the total RNA, measured using the Bioanalyser. Libraries were subject to cluster formation and then 72 base single end sequencing using a Hiseq analyser. Raw and processed data are online, associated with the GEO Series ID GSE75667.

RNA-Seq data analysis

The RNA-seq data were all aligned to the mm9 mouse genome using BWA (default settings). The reads were aligned to the gene features annotated in RefSeq. All reads within an annotated RefSeq gene ("All reads") and reads containing intronic sequences ("Intronic reads") were annotated using the bam file obtained by BWA as input. The data was then normalised against a set of invariant genes, identified using a method that assumed a quasi-normal distribution of gene readcounts. The RNAseq readcounts in each sample was expressed relative to one sample used as reference. Since the distribution of invariant gene readcounts should be approximatively Gaussian, we obtained

the μ_{diff} and σ_{diff} parameters of the best-fit Gaussian using the dnorm() function in R and optimised by maximum likelihood with the optim() function. Invariant genes were defined as those with differences in read counts within 1σ from the mean difference (μ_{diff}).

Differential gene expression (DGE) analysis was performed with Deseq (Anders and Huber, 2010) (comparing resting and TPA-induced, wildtype and TKO MEF samples adj-p ≤0.01, minimum change of 10%). We estimated the effect of knockout and reconstituted background by using a method that compares the degree of gene induction under two different conditions and identifies genes that are similarly affected by background. The basic assumption is that activity of genes dependent on a particular transcriptional regulator will be similarly affected by changes in abundance or activity of that regulator. The method does not require paired conditions; however, although it can give an overall estimate of the numbers of genes subject to shared control, it does not allow statistical conclusions to be drawn regarding particular genes.

Written in R, the pipeline compares the TPA-induced fold change in one background (x-axis: fold change in background 1), to the difference between the fold change in that background and a second background (y-axis: [fold-change in background 1]-[fold-change in background 2]). Systematic influence of the second background will generate a distribution asymmetrically disposed about y=0, and linear regression analysis of data points above and below y=0 should yield slopes that are significantly non-zero. For perfect dependence of the induction on the second background, linear regression of the data above y=0 should be 1. In contrast, differences between the signal in the two

backgrounds that arise from random technical variations will be distributed around y=0, and linear regression of points above or below y=0 should give a slope of zero. For each comparison, the linear fit for data-points with y>0 (impaired induction in the second background) and y<0 (enhanced induction in the second background) was compared to y=0. Data-points presenting a significant bias were then filtered through an iterative loop where each data-point was either included or excluded according to its Euclidean distance to the line y=0 or to the regression line going though the data-points under investigation.

To be classified as a TCF-SRF target, a gene has to satisfy several criteria: significant change in expression upon TPA stimulation; significant change in degree of induction upon TCF inactivation; and association with SRF binding sites identified by ChIP-seq, either within 10kb or exhibiting significant interaction with remote SRF sites according to Hi-C analysis. Since for each assay the FDR is below 5%, the overall FDR can be considered to be negligible.

Chromatin immunoprecipitation

ChIP was performed as described (Esnault et al., 2014; Miralles et al., 2003), with the following modifications: fixation was stopped by the addition of 250 mM glycine, sonication was performed with a Bioruptor® Plus and magnetic G-protein beads (Invitrogen) were used for recovery. SYBR Green-based real-time PCR (Invitrogen) was performed using dilutions of genomic DNA solution for calibration and to derive arbitrary abundance units. We assessed

SRF and MRTF DNA binding (see Figure 6 and S6) using primers : Acta2 5’-GAGGCCTGGGTCTCTTCCA-3’, 5’- GCTGAGCTGCCTCCTGTTTC-3’ ; Ankrd1 5’- ACCTACAGTCTCTTCCAAACCATGT-3’, 5’-CCAGTGAGCAGAGCAATTTCC-3’ ; Slc2a1 5’- CCTGCGCCCCTTACATCA -3’, 5’- GTGTTCCTGGTAGGGCTTGAAA -3’ ; Control (Zfp37) 5’-CCAGCAATGTGTGACTTGGATC-3’, 5’-TATTTCGAGCGCTGTGGCA-3’ ; Egr1_p: 5’-CGGAAACGCCATATAAGGAGC-3’, 5’-ATATAAGGCGCTGCCCAAA-3’; Egr2_p: 5’-AATCGTTCCT- GGCGAGCTC-3’, 5’-GCAGCTTTTGCCGTCACAT-3’; Egr3_p: 5’-AGTT CCCTGGCTGGGAGCT-3’, 5’-GCTGCTATCACCACCAACCA-3’, Nr4a1_p: 5’-CGGCTTGGTGAGCCTAGTG-3’, 5’-AAATAGTTTTCCTCTCGCCCG- 3’

Antibodies

Antibodies used were: SRF (sc-335, lot# D3013 S. Cruz); MRTF-A (sc-21558, lot# I1412, S. Cruz) Elk-1 anti-mouse Elk-1 aa309–429 was made in-house (Costello et al., 2010) and affinity-purified against recombinant human Elk-1^{nonA} (aa309-429) before use.

Chip-Seq sample preparation

DNA samples were end repaired, poly-A tailed and Illumina single-end adapters ligated following the standard Illumina protocol with minor adjustments. Agencourt AMPure XP beads at 0.8x ratio were used to remove adapter dimers after ligation. The Illumina kit Phusion enzyme was replaced

by Kapa HiFi HotStart ready mix. Post PCR, AMPure XP beads were used at a 1:1 ratio to maintain size integrity. DNA fragments size-selected and purified from a 2% agarose gel using the QIAquick gel extraction kit, quality controlled on the DNA 1000 BioAnalyser 2100 chip. Sequencing was on the Hi-seq2500 to generate 150 base read lengths.

ChIP-seq analysis

All ChIP-seq samples have been aligned using BWA to the mm9 (default settings). Raw and processed data are online on GEO, Series ID GSE75667. Candidate SRF and Elk-1 sites were first identified using MACS (Zhang et al., 2008) to identify regions enriched over background (beads alone) in each aligned sample. Default settings and MACS threshold values of p<1E-4 were used. Peaks were merged with basic functions in bedtools, obtaining ~1/2 million unique peaks identified in any of the 8 SRF samples (SRF ChIP in wildtype MEF and TKO MEF in 0.3%FCS and TPA, two replicates per condition per background) and in any of the 6 Elk-1 samples (Elk-1 ChIP-seq perfomend in TKO MEF reconstituted with Elk-1^{WT}, Elk-1^{FW} or Elk-1^{nonA} in 0.3% FCS and TPA, one replicate per condition per background).

To identify ChIP-seq peaks, we first filtered peak set according to peak concidence, retaining those peaks called by MACS at p<1E-4 in at least 3 pseudo-replicate samples (3 out of the 8 SRF samples and 3 out of the 6 Elk-1 samples), giving a total of ~20000 unique peaks. The rationale for using occurence of a peak-call in 3 pseudoreplicates to assign a candidate ChIP-

seq peak was based on an assessment of the probability that overlap of MACS-called peaks occurs by chance. To do this, we iteratively simulated in R randomly distributed ChIP-seq peaks across the mm9 genome, considering a series of parameters was considered including: frequency distribution of the peak width observed across the ~1/2 million peaks; the number of peaks called per chromosome, and therefore each chromosome length and the number of peaks called per sample. We generated ~1/2 million random peak coordinates fullfilling the above criteria and counted how often out of 10000 iterations any given coordinate was overlapping with 1, 2, 3 or more peaks. This yielded empirical estimated FDRs for the coincidence of MACS-called peaks as: 0.5E-2 for one, 1E-3 for 2, 2.5E-5 for 3 and 3.4E-6 for 4 coincidences. We therefore chose 3 peak coincidences as our filter criterion.

Tools which allow identification of ChIP-seq peaks are prone to false positives (Krebs et al., 2014) owing to signal-to-noise-ratio and variables such as antibody affinity and specificity. To assess the validity of the identified ~20000 peaks we therefore assessed whether the read distribution in SRF and Elk-1 ChIP-seq samples was substantially different from ChIP-seq of Elk-1 in TKO MEF transduced with empty vector. These ChIP-seq samples are the perfect control for Elk-1 ChIP-seq, and it is also valid to assume that SRF ChIP-seq peaks should present read-counts which distribute differently from those generated by an unrelated antibody in cells lacking the cognate epitope. Comparison of the read counts was performed using Deseq (minimal increase relative to control of 1.5-fold, $p \leq 0.05$) comparing the control Elk-1 ChIP-seq (two pseudo-replicates: performed in TKO MEF rescued with Vehicle in 0.3% FCS and TPA-stimulated conditions) to the 6 Elk-1 ChIP-seq samples or to

the SRF samples. The read counts showed a skewed distribution. Of the ~20000 peaks, 2662 unique peaks passed this test, with 2577 showed significant signal for SRF and 336 showed signal for Elk-1. We used the same approach to identify those SRF peaks which dropped in the TKO MEF if compared to wildtype MEFs, comparing the 4 SRF CHIP-seq pseudo-replicates in wildtype MEF and TKO MEFs. Together this approach will give an effectively negligible FDR.

Identification of TF Binding Motifs

Discovery of known motif was performed using HOMER (Heinz et al., 2010) on sequences ±100 bp from the peak summit of ChIP-seq identified regions or DNAseI hypersensitive sites.

Hi-C analysis

The HOMER Hi-C software analysis pipeline (http://biowhat.ucsd.edu/homer/interactions/) was used to determine significant interactions and perform PCA compartment analysis. Paired-end reads from 3 different datasets were used: SRX554530 (Battulin et al., 2015), GSM1648486 and GSM1696042 (Minajigi et al., 2015) Reads were aligned independently to the mouse reference genome assembly (NCBI37/mm9) using Bowtie2 (Li and Durbin, 2009) by the iterative method (Imakaev et al., 2012), starting with a minimum length of 26bp. Each read was then trimmed to have equal length relative to 5’, and unmapped and unpaired reads were removed. Reads were then filtered to remove duplicate read pairs (“-tbp 1”),

paired-end reads likely representing continuous genomic fragments or religation events (‘‘-removePEbg’’) and self-ligations (‘‘-removeSelfLigation’’). This approach generated a total of ~460 million uniquely mapped PE reads. Of these only read-pairs where both ends mapped near the HindIII (AAGCTT) restriction sites were retained (‘‘-both’’) using a distance threshold as 1.5x the fragment length estimated with Homer tools.

To identify interactions, we first determined a ‘‘universe’’ of interactions if compared to the background model, therefore taking into account linear genomic distance and sequencing depth (p ≤0.05 and z-score ≥2). The identification of interaction was performed looking at overlapping windows (“-res 5000” and “-superRes 10000”), allowing removal of redundant interactions and retention of those that are significant. Each anchor point was re-centred according to the average of the positions contributing to it (“-center”). This analysis identified ~1.3 million significant interactions at 10kb resolution. Interactions mapping at all Refseq annotated TSSs (~130000) were retrieved using the annotateInteractions.pl pipeline in Homer.

Chromosomal regions exhibiting preferential interactions were identified by using the automated PCA analysis on Hi-C data in HOMER (runHiCpca.pl) based on the Lieberman-Aiden strategy to identify minimal components that describe most of the high dimensional dataset (Lieberman-Aiden et al., 2009). These regions, denoted A and B according to PCA score, are analogous to the previously defined A and B compartments (Lieberman-Aiden et al., 2009). They are defined as those with high contacts *in cis* and low contacts i*n trans* as extensively described in the HOMER website (http://homer.salk.edu/homer/interactions/HiCpca.html). The identified

interactions obtained with the runHiCpca.pl script were then refined to reduce small PCA inversions. We assigned a score per region as function of its average absolute PCA score and its length (abs(Homer average PCA score) * region length/10kb). As the resolution used is 10kb, this represents the smallest region length. Regions with a length-normalised PCA score of >100 were retained, and those scoring below this were merged with their neighbours according to their PCA sign.

Cell morphology, ECM-remodelling and invasion assays.

Highthroughput imaging was used to quantify actin stress fibre length. F-actin were stained with phalloidin, and fibre length (contiguous pixels emitting light) analysed using the Cellomics ArrayScan VTI and Compartmental analysis (BioApplications). 3000 cells were counted per determination.

Force-mediated matrix remodelling (Calvo et al., 2013) was performed by embedding $50x10^3$ fibroblasts in 100µl of collagen I/Matrigel and seeded on a 35-mm glass-bottom MatTek dish (P35-1.5-14-C, MatTek). Once the gel was set, the fibroblasts embedded gel was manteined in DMEM+10% FCS with + 1% ITS (insulin–transferrin–selenium; #41400-045; Invitrogen. Gel sizes were obtained using ImageJ software, and gel contraction values represent % of original gel size 10-12h after seeding.

The fibroblast organotypic culture system was set up as described (Calvo et al., 2013) with minor modifications. $5x10^5$ fibroblasts were embedded in a mixture of collagen I/Matrigel matrix and 1ml of this mix was set into 24 well-plates. After setting the gel at 37 °C for 1 h, DMEM+10% FCS+1% ITS was

added on the top. The day after $5×10^5$ 4T1 breast cancer cells were seeded on top of each gel in DMEM +10% FCS+1% ITS. The day after the gel was mounted on a metal bridge and fed from underneath with DMEM +10% FCS + 1% ITS (changed daily). After 5 days, the cultures were fixed in 4% paraformaldehyde plus 0.25% glutaraldehyde in PBS and processed by standard methods for haematoxylin and eosin staining.

SUPPLEMENTAL REFERENCES

Anders, S., and Huber, W. (2010). Differential expression analysis for sequence count data. Genome Biol *11*, R106.

Battulin, N., Fishman, V.S., Mazur, A.M., Pomaznoy, M., Khabarova, A.A., Afonnikov, D.A., Prokhortchouk, E.B., and Serov, O.L. (2015). Comparison of the three-dimensional organization of sperm and fibroblast genomes using the Hi-C approach. Genome Biol *16*, 77.

Calvo, F., Ege, N., Grande-Garcia, A., Hooper, S., Jenkins, R.P., Chaudhry, S.I., Harrington, K., Williamson, P., Moeendarbary, E., Charras, G., *et al.* (2013). Mechanotransduction and YAP-dependent matrix remodelling is required for the generation and maintenance of cancer-associated fibroblasts. Nature cell biology *15*, 637-646.

Costello, P., Nicolas, R., Willoughby, J., Wasylyk, B., Nordheim, A., and Treisman, R. (2010). Ternary complex factors SAP-1 and Elk-1, but not net, are functionally equivalent in thymocyte development. J Immunol *185*, 1082-1092.

Cruzalegui, F.H., Cano, E., and Treisman, R. (1999). ERK activation induces phosphorylation of Elk-1 at multiple S/T-P motifs to high stoichiometry. Oncogene *18*, 7948-7957.

Esnault, C., Stewart, A., Gualdrini, F., East, P., Horswell, S., Matthews, N., and Treisman, R. (2014). Rho-actin signaling to the MRTF coactivators dominates the immediate transcriptional response to serum in fibroblasts. Genes Dev *28*, 943-958.

Heinz, S., Benner, C., Spann, N., Bertolino, E., Lin, Y.C., Laslo, P., Cheng, J.X., Murre, C., Singh, H., and Glass, C.K. (2010). Simple combinations of lineage-determining transcription factors prime cis-regulatory elements required for macrophage and B cell identities. Molecular cell *38*, 576-589.

Imakaev, M., Fudenberg, G., McCord, R.P., Naumova, N., Goloborodko, A., Lajoie, B.R., Dekker, J., and Mirny, L.A. (2012). Iterative correction of Hi-C data reveals hallmarks of chromosome organization. Nat Methods *9*, 999-1003.

Krebs, W., Schmidt, S.V., Goren, A., De Nardo, D., Labzin, L., Bovier, A., Ulas, T., Theis, H., Kraut, M., Latz, E., *et al.* (2014). Optimization of transcription factor binding map accuracy utilizing knockout-mouse models. Nucleic Acids Res *42*, 13051-13060.

Li, H., and Durbin, R. (2009). Fast and accurate short read alignment with Burrows-Wheeler transform. Bioinformatics *25*, 1754-1760.

Lieberman-Aiden, E., van Berkum, N.L., Williams, L., Imakaev, M., Ragoczy, T., Telling, A., Amit, I., Lajoie, B.R., Sabo, P.J., Dorschner, M.O., *et al.* (2009). Comprehensive mapping of long-range interactions reveals folding principles of the human genome. Science *326*, 289-293.

Marais, R., Wynne, J., and Treisman, R. (1993). The SRF accessory protein Elk-1 contains a growth factor-regulated transcriptional activation domain. Cell *73*, 381-393.

Minajigi, A., Froberg, J.E., Wei, C., Sunwoo, H., Kesner, B., Colognori, D., Lessing, D., Payer, B., Boukhali, M., Haas, W., *et al.* (2015). Chromosomes. A comprehensive Xist interactome reveals cohesin repulsion and an RNA-directed chromosome conformation. Science *349*.

Miralles, F., Posern, G., Zaromytidou, A.I., and Treisman, R. (2003). Actin dynamics control SRF activity by regulation of its coactivator MAL. Cell *113*, 329-342.

Panayiotou, R., Miralles, F., Pawlowski, R., Diring, J., Flynn, H., Skehel, M., and Treisman, R. (2016). Phosphorylation acts positively and negatively to regulate MRTF-A subcellular localisation and activity. eLife *5*, e15460.

Price, M.A., Rogers, A.E., and Treisman, R. (1995). Comparative analysis of the ternary complex factors Elk-1, SAP-1a and SAP-2 (ERP/NET). EMBO J *14*, 2589-2601.

Zhang, Y., Liu, T., Meyer, C.A., Eeckhoute, J., Johnson, D.S., Bernstein, B.E., Nusbaum, C., Myers, R.M., Brown, M., Li, W., *et al.* (2008). Model-based analysis of ChIP-Seq (MACS). Genome Biol *9*, R137.
