# Supplementary material for: SRF Co-factors Control the Balance between Cell Proliferation and Contractility
Source: Mol Cell. 2016 Dec 15;64(6):1048–61. doi: 10.1016/j.molcel.2016.10.016 (PMC5179500; doi:10.1016/j.molcel.2016.10.016)
Supplement: Document S2. Article plus Supplemental Information [file mmc8.pdf]

Molecular Cell

Article

SRF Co-factors Control the Balance between Cell Proliferation and Contractility

Graphical Abstract

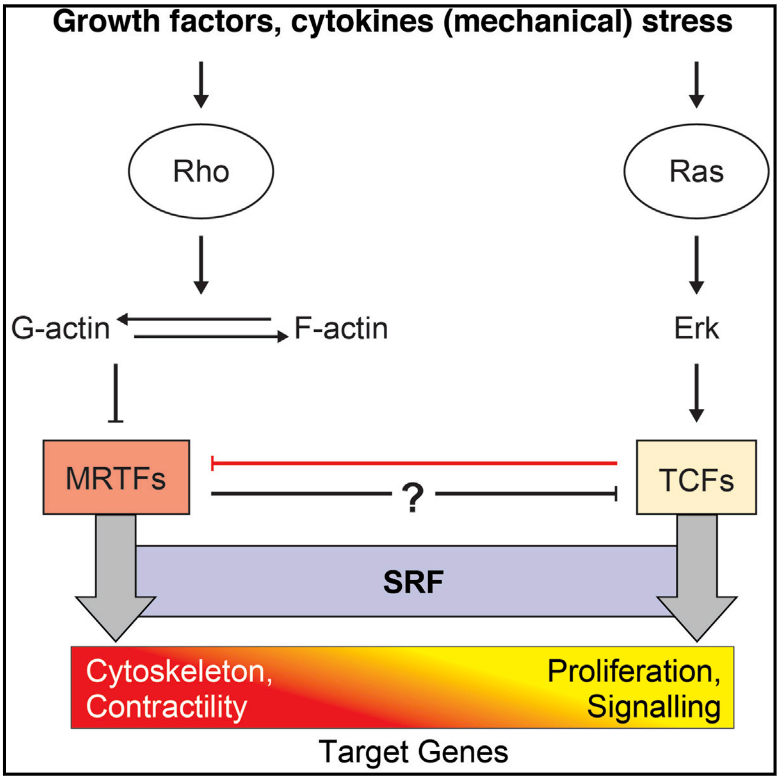

Authors

Francesco Gualdrini, Cyril Esnault, Stuart Horswell, Aengus Stewart, Nik Matthews, Richard Treisman

Correspondence

richard.treisman@crick.ac.uk

In Brief

Two cofactor families, the TCFs and the MRTFs, compete for binding to SRF, a key regulator of the mitogen-responsive transcription. TCFs not only control proliferative gene expression programs but also regulate access of the MRTFs to SRF, thereby regulating cell contractility and pro-invasive behavior.

Highlights

- Integrated ChIP-seq Hi-C analysis identifies over 700 TCF-dependent SRF target genes
- Over 60% of TPA-inducible gene transcription is TCF-dependent
- TCF-dependent transcription potentiates cell proliferation
- TCF/MRTF competition for SRF determines contractility and pro-invasive behavior

Accession Numbers

GSE75667

Gualdrini et al., 2016, Molecular Cell *64*, 1048–1061
December 15, 2016 © 2016 The Author(s). Published by Elsevier Inc.
http://dx.doi.org/10.1016/j.molcel.2016.10.016

SRF Co-factors Control the Balance between Cell Proliferation and Contractility

Francesco Gualdrini,[1,4] Cyril Esnault,[1,4,5] Stuart Horswell,[2] Aengus Stewart,[2] Nik Matthews,[3] and Richard Treisman[1,6,*]
[1]Signalling and Transcription Group
[2]Bioinformatics and Biostatistics STP
[3]Advanced Sequencing STP
Francis Crick Institute, 1 Midland Rd, London NW1 1AT, UK
[4]Co-first author
[5]Present address: Transcription and Epigenomics in Developing T Cells Group, Institut de Génétique Moléculaire de Montpellier CNRS-UMR 5535, 1919 Route de Mende, Cedex 5, 34293 Montpellier, France
[6]Lead Contact
*Correspondence: richard.treisman@crick.ac.uk
http://dx.doi.org/10.1016/j.molcel.2016.10.016

SUMMARY

The ERK-regulated ternary complex factors (TCFs) act with the transcription factor serum response factor (SRF) to activate mitogen-induced transcription. However, the extent of their involvement in the immediate-early transcriptional response, and their wider functional significance, has remained unclear. We show that, in MEFs, TCF inactivation significantly inhibits over 60% of TPA-inducible gene transcription and impairs cell proliferation. Using integrated SRF ChIP-seq and Hi-C data, we identified over 700 TCF-dependent SRF direct target genes involved in signaling, transcription, and proliferation. These also include a significant number of cytoskeletal gene targets for the Rho-regulated myocardin-related transcription factor (MRTF) SRF cofactor family. The TCFs act as general antagonists of MRTF-dependent SRF target gene expression, competing directly with the MRTFs for access to SRF. As a result, TCF-deficient MEFs exhibit hypercontractile and pro-invasive behavior. Thus, competition between TCFs and MRTFs for SRF determines the balance between antagonistic proliferative and contractile programs of gene expression.

INTRODUCTION

Ras-ERK signaling is critical for control of proliferation, invasion, and metastasis. Ras activation stimulates cell-cycle re-entry from quiescence, and Ras genes are mutated in around 20% of human cancers (Chambard et al., 2007; Pylayeva-Gupta et al., 2011). Ras-ERK signaling plays an important role in this immediate-early (IE) transcriptional response to mitogen stimulation, which activates many genes encoding transcription factors, including Myc and members of the AP1 and Egr families (Cochran et al., 1984; Greenberg and Ziff, 1984; Kelly et al., 1983). Many IE genes are controlled by the transcription factor serum response factor (SRF, *Srf*), which is required for Ras-induced cell-cycle re-entry but not for proliferation per se (Gauthier-Rouvière et al., 1990; Schratt et al., 2001).

SRF acts in partnership with two families of signal-regulated cofactors. The three ternary complex factors (TCFs) Elk-1, Net, and SAP-1 (*Elk1*, *Elk3*, and *Elk4*) are Ets domain proteins, regulated by Ras-ERK signaling (Buchwalter et al., 2004; Shaw et al., 1989), whereas the myocardin-related transcription factors (MRTFs) *Mkl1* and *Mkl2* respond to the Rho-actin pathway (Miralles et al., 2003; Olson and Nordheim, 2010). The TCFs and MRTFs interact competitively with the SRF DNA-binding domain (Miralles et al., 2003; Zaromytidou et al., 2006). Whether cofactor competition is a general feature of SRF regulation in vivo has been unclear. In fibroblasts, some IE genes appear to be specifically coupled to one pathway or the other, but in smooth muscle cells, platelet-derived growth factor (PDGF) can induce cofactor exchange (Wang et al., 2004).

Genetically, the TCFs are at least partly functionally redundant (Costello et al., 2010; Weinl et al., 2014) and may also function independently of SRF (Boros et al., 2009a, 2009b; Buchwalter et al., 2005). Although they have been implicated in proliferation and cancer (Vickers et al., 2004; Wozniak et al., 2012; Yang et al., 2012), the extent to which the immediate-early transcriptional response is TCF-dependent, and the target genes involved, has not been systematically investigated. In contrast, the MRTFs mediate morphogenetic, adhesive, and motile processes (Miralles et al., 2003; Olson and Nordheim, 2010; Schratt et al., 2002).

We showed previously that much of the serum-induced immediate transcriptional response is MRTF/SRF-dependent (Esnault et al., 2014); however, the lack of specific TCF inhibitors and the relatively poor quality of TCF chromatin immunoprecipitation (ChIP) data precluded rigorous analysis of the role of TCF-SRF signaling. Here we used wild-type and triply TCF-deficient mouse embryonic fibroblasts (MEFs) to directly address the role of the TCFs in the transcriptional response to 12-O-tetradecanoyl phorbol-13-acetate (TPA)-induced ERK activation. We show that the majority of the immediate transcriptional response is TCF-dependent, either directly or indirectly. TCF-deficient

 © 2016 The Author(s). Published by Elsevier Inc.
This is an open access article under the CC BY license (http://creativecommons.org/licenses/by/4.0/).

A

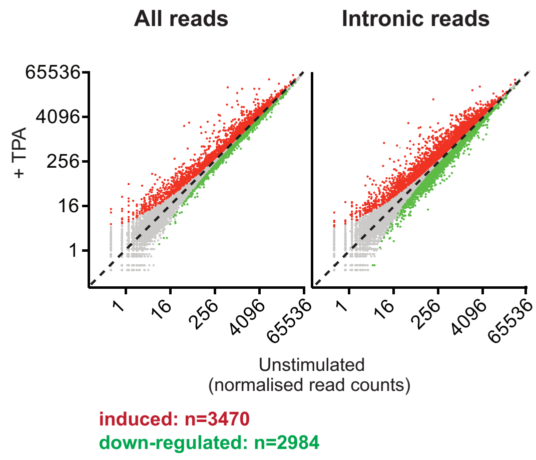

B

Hallmark signatures	p-val	n	
**SRF Signature**	**1E-86**	**372**	induced
**MRTF Signature**	**6E-48**	**244**	induced
TNFA SIGNALING VIA NFKB	2E-69	131	induced
G2M CHECKPOINT	6E-36	104	induced
MTORC1 SIGNALING	4E-34	100	induced
MYC TARGETS V1	9E-34	102	induced
E2F TARGETS	5E-20	82	induced
**TCF Signature**	**3E-17**	**38**	induced
UNFOLDED PROTEIN RESPONSE	8E-16	53	induced
APOPTOSIS	2E-15	63	induced
P53 PATHWAY	2E-15	73	induced
PI3K AKT MTOR SIGNALING	3E-15	49	induced
HYPOXIA	8E-15	72	induced
IL2 STAT5 SIGNALING	2E-13	67	induced
OXIDATIVE PHOSPHORYLATION	7E-20	75	down-regulated
**MRTF Signature**	**2E-12**	**149**	down-regulated
**SRF Signature**	**1E-10**	**186**	down-regulated
ADIPOGENESIS	2E-10	59	down-regulated
FATTY ACID METABOLISM	9E-07	42	down-regulated
HEME METABOLISM	3E-05	44	down-regulated
GLYCOLYSIS	2E-04	44	down-regulated
E2F TARGETS	2E-04	46	down-regulated
EC MSC TRANSITION	7E-04	44	down-regulated

C

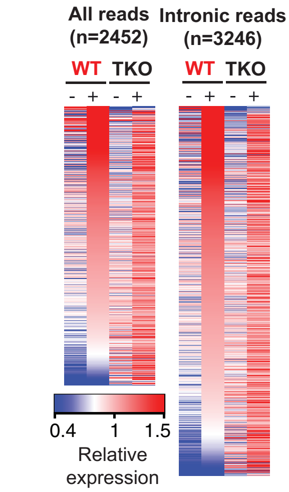

D

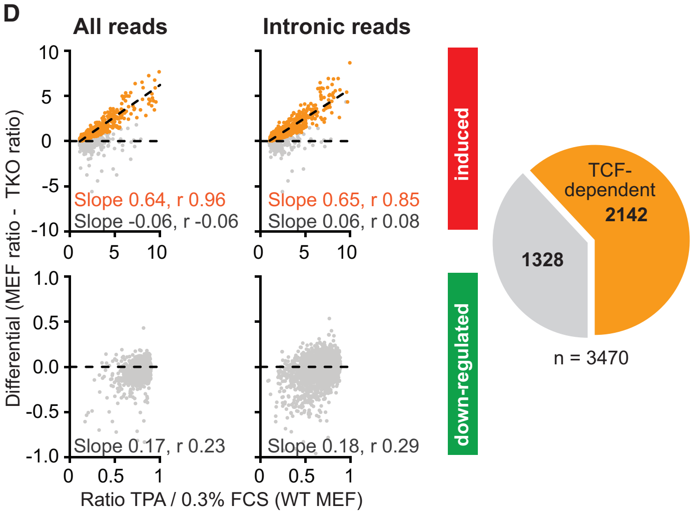

E

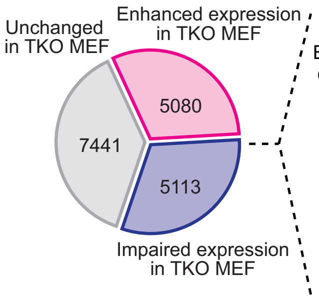

Hallmark signatures	p-val	n	
**SRF Signature**	**1.7E-105**	**491**	Enhanced in TKO MEF
**MRTF Signature**	**2.3E-97**	**383**	Enhanced in TKO MEF
MTORC1 Signaling	5.9E-31	116	Enhanced in TKO MEF
Hypoxia	1.4E-18	99	Enhanced in TKO MEF
Apical Junction	2.5E-15	93	Enhanced in TKO MEF
Myogenesis	6.9E-14	92	Enhanced in TKO MEF
Estrogen Response Early	1.0E-12	89	Enhanced in TKO MEF
Cholesterol Homeostasis	1.1E-12	43	Enhanced in TKO MEF
Glycolysis	1.6E-12	87	Enhanced in TKO MEF
P53 Pathway	5.0E-12	87	Enhanced in TKO MEF
Estrogen Response Late	5.2E-12	86	Enhanced in TKO MEF
E2F Targets	2.9E-55	144	Impaired in TKO MEF
G2M Checkpoint	2.7E-36	125	Impaired in TKO MEF
Mitotic Spindle	4.0E-09	81	Impaired in TKO MEF
UV Response DN	3.2E-05	56	Impaired in TKO MEF
Apoptosis	2.8E-04	57	Impaired in TKO MEF
Myc Targets V1	3.3E-04	69	Impaired in TKO MEF
Dna Repair	8.5E-04	51	Impaired in TKO MEF
TNFa Response	4.8E-03	34	Impaired in TKO MEF
Kras Signaling UP	0.02	61	Impaired in TKO MEF
TNFa Signaling Via Nfkb	0.02	62	Impaired in TKO MEF

F

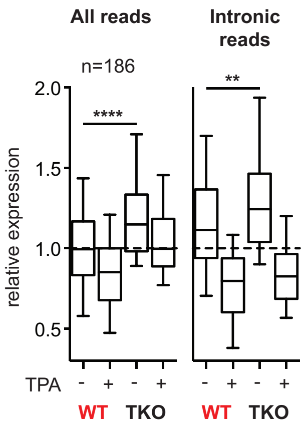

(legend on next page)

MEFs proliferate slowly, and TCF-dependent SRF target genes, which include *Myc,* are predominantly associated with cell signaling, metabolism, and proliferation. Strikingly, the TCFs also act as general negative regulators of cell adhesion, contractility, and motility by inhibiting access of MRTFs to SRF at its target genes.

RESULTS

TPA Treatment Both Activates and Downregulates Gene Transcription

To activate TCF-SRF signaling in MEFs, we used the phorbol ester TPA, which activates ERK via protein kinase C (PKC) and RasGRP1 (Griner and Kazanietz, 2007). TPA also downregulates Rho-actin signaling (Panayiotou et al., 2016) and thus allows the identification of genes that are particularly sensitive to TCF activation. In immortalized MEFs, TPA activated classical TCF-SRF targets such as *Egr1* but not MRTF-SRF target genes such as *Vcl*, although all were activated by serum stimulation (Figure S1A). We used RNA sequencing (RNA-seq) to analyze the global response to TPA stimulation, analyzing both total and intronic RNA-seq reads to maximize sensitivity (Figure 1A; Table S1). The TPA-induced gene set is enriched in gene hallmarks (i.e., gene cohorts that change similarly in different contexts; Subramanian et al., 2005) associated with the cell cycle, signaling networks, and particular transcription factors (Figure 1B; Table S2A) and gene ontology (GO) terms for genes involved in signaling, metabolism, the cell cycle, and the cytoskeleton (Table S2B). It was also significantly enriched for genes from both the TCF- and MRTF-SRF gene signatures (Esnault et al., 2014; Figure 1B), suggesting that many MRTF-SRF target genes are also directly or indirectly controlled by the TCFs (see below). In contrast, TPA-downregulated genes were associated with hallmarks involving specific cellular functions, particularly metabolism; MRTF-SRF target genes were also enriched in this group, consistent with TPA downregulating Rho (Figure 1B; Tables S2A and S2B; see below).

TCFs Promote Cell Proliferation and Control Most TPA-Induced Transcription

To investigate the role of TCFs in gene expression and cell behavior, we studied MEFs from embryos lacking all three TCFs (triple knockout [TKO] MEFs; Costello et al., 2010). Strikingly, TKO MEFs were significantly less proliferative than wild-type MEFs (Figure S1B), with increased numbers of cells in G2/M compared with wild-type cells (Figure S1C). They also exhibited somewhat enhanced ERK activation in response to TPA (Figure S1D), probably reflecting aberrant expression of ERK phosphatases (Buffet et al., 2015). Nevertheless, TPA induction of classical TCF-linked immediate-early genes was effectively abolished in TKO MEFs (Figure S1E), although serum induction of SRF target genes was less impaired (Figures S1A, S1E, and S1F).

RNA-seq analysis revealed that the global transcriptional response to TPA was substantially altered in TKO MEFs (Figure 1C). Using an iterative pipeline to compare gene inducibility in the two different backgrounds, we partitioned the genes induced by TPA in wild-type MEFs into TCF-dependent and -independent groups (Supplemental Experimental Procedures). Over 60% of TPA-induced gene expression was TCF-dependent (estimated at 2,142 of 3,470), whereas TPA-downregulated gene expression showed no obvious TCF-dependence (Figure 1D). Thus, consistent with their known biochemical role in gene activation, the TCFs act positively in the transcriptional response to TPA stimulation.

TCF inactivation also changed the basal expression of approximately 60% of genes detectably transcribed in wild-type MEFs, regardless of their acute response to TPA stimulation (Figure 1E). Consistent with the proliferative defect observed in TKO cells, genes with reduced basal expression were enriched in gene hallmarks and GO terms associated with cell-cycle and proliferative responses (Figure 1E; Tables S2A and S2B). In contrast, genes exhibiting increased basal transcription in TKO cells included a substantial number of genes previously identified as MRTF-SRF targets (383 of 683; Esnault et al., 2014) predominantly associated with cytoskeletal hallmarks and GO terms (Figure 1E; Tables S2A and S2B). Many of these genes were downregulated in response to TPA in wild-type MEFs (Figure 1F). Thus, the TCFs not only mediate the acute response to ERK activation but also negatively regulate MRTF-SRF signaling (see below).

Integration of SRF ChIP-Seq and Hi-C Datasets Identifies Candidate SRF Target Genes

To identify TCF-dependent SRF target genes, we set out to correlate the RNA-seq data with SRF ChIP sequencing

Figure 1. Much of the TPA-Induced Transcriptional Response Is TCF-Dependent

(A) Scatterplot displaying total and intronic RNA-seq read counts before and after 30 min of TPA stimulation. Induced genes are highlighted in red and downregulated genes in green (FDR = 0.01, fold change $\geq$ 10%).

(B) The relation between TPA-induced and -downregulated genes and MsigDB hallmark gene set signatures was assessed by hypergeometrical test. p-val, Bonferroni-adjusted p value.

(C) Heatmap representation of the relative expression of TPA-induced genes in wild-type and TKO MEFs ranked by absolute change in transcription induced by TPA in wild-type MEFs.

(D) Identification of TCF-dependent genes by comparison with the TPA induction ratio in wild-type and TKO MEFs using both total and intronic RNA-seq reads. TCF-dependent genes (orange) exhibit a systematic relationship between their degree of induction in the two contexts whereas others (gray) do not; slope and Spearman r are indicated. Right: data summary.

(E) Changes to basal gene expression in TKO MEFs. The relation between genes whose basal expression is increased (top) or decreased (bottom) in TKO MEFs and MsigDB hallmark gene set signatures was assessed as in (B).

(F) TPA-downregulated SRF targets are TCF-independent but exhibit increased baseline expression in TKO MEFs. Boxplots present the average fold change of total and intronic RNA. Center line, median; top and bottom edges, 75th and 25th percentiles. ****$p < 0.0001$, **$p < 0.01$ (Wilcoxon matched pair signed-rank test).

See also Figure S1 and Tables S1 and S2.

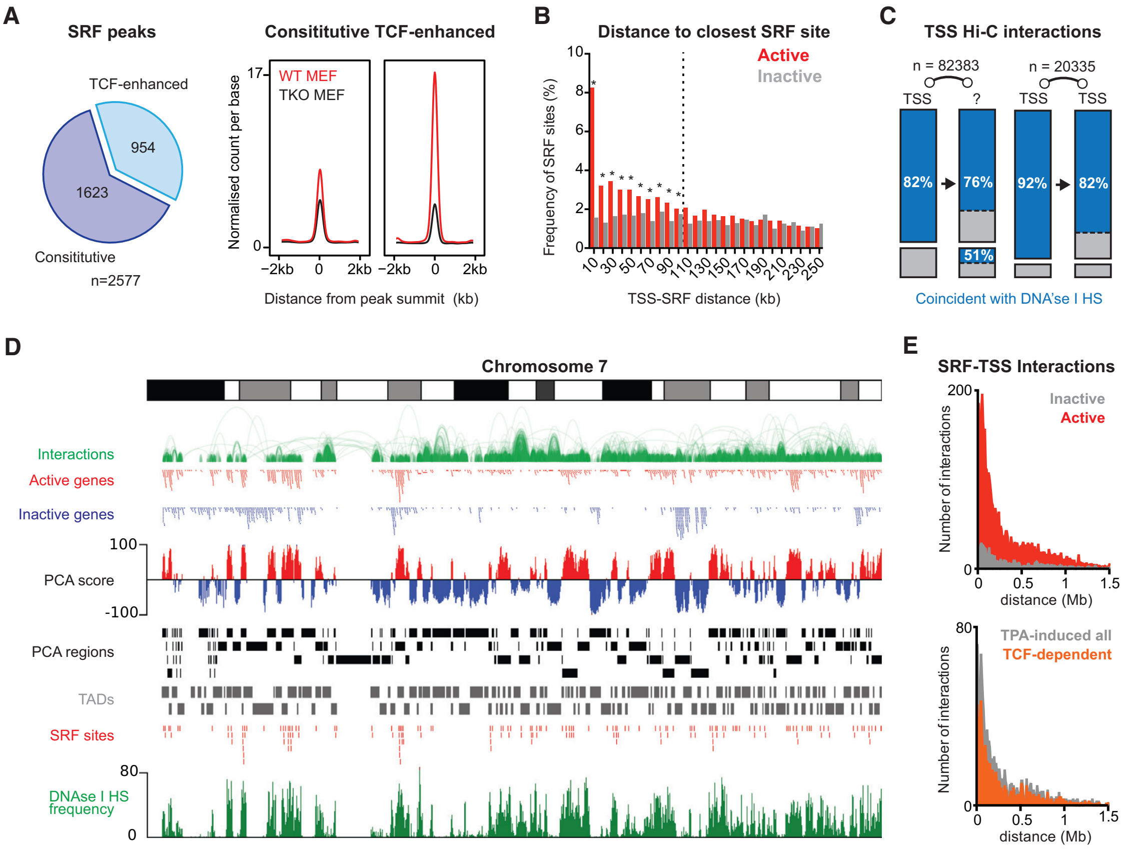

Figure 2. Integration of SRF ChIP-Seq in MEFs with Hi-C Interaction Data

(A) The TCFs enhance SRF binding at many sites. Metaprofiles of the SRF ChIP-seq signal at sites where SRF binding is unaffected by TCFs (constitutive) or where they enhance it (TCF-enhanced).

(B) Comparison of the frequency of SRF sites at increasing distances from active and inactive gene TSSs (10-kb bins). *, significant at $p < 0.05$ (multiple t test with Holmes-Sidak correction).

(C) Most TSS Hi-C interactions in MEFs coincide with DNase I HS at 10-kb resolution. Left: TSS interactions with non-TSS regions. Right: TSS-TSS interactions. Blue, interactions involving regions containing at least one DNase I HS.

(D) Hi-C analysis. Chromosome 7 is shown with tracks as follows: significant Hi-C interactions displayed using the WashU EpiGenome browser; active (red) and inactive (blue) RefSeq-annotated genes identified by RNA-seq; PCA analysis of Hi-C data with A regions (positive PCA score) shown in red and B regions (negative PCA score) in blue; comparison of individual PCA regions (black) with the TADs (gray) previously annotated in MEFs (Battulin et al., 2015); SRF binding sites; and DNase I HS frequency per base.

(E) The relation between TSS-SRF interaction distance in Hi-C and gene activity defined by RNA-seq. Top: interactions involving active (red) or inactive (gray) TSSs. Bottom: interactions with TPA-induced TSSs; those whose induction is TCF-dependent are highlighted in orange.

See also Figure S2.

(ChIP-seq). We first identified 2,577 high-confidence SRF binding sites (Figure 2A; Figure S2A; Table S3). In TKO MEFs, ∼40% of these showed reduced SRF binding and increased H3 occupancy, suggesting that cooperative TCF-SRF binding facilitates nucleosome exclusion (Esnault et al., 2014; Figure S2B). SRF sites were statistically overrepresented within 100 kb of active genes in MEFs (Figure 2B), coinciding with DNase I-hypersensitive sites (DNase I HS) (Figures S2C–S2E; see GEO: GSM1003831 and GSM1014199). They were associated with the same transcription factor (TF) binding motifs as in NIH 3T3 fibroblasts (Esnault et al., 2014), although, at TCF-enhanced sites, Ets motifs were more prevalent, suggesting that TCF-SRF interaction facilitates SRF binding (Figure S2F).

Integration of Hi-C chromosome interaction maps with ChIP-seq data can reliably identify signal-induced genes controlled by remote regulatory sites (Jin et al., 2013). We therefore integrated the SRF ChIP-seq data with recently determined Hi-C maps of intrachromosomal interactions in MEFs (Battulin et al., 2015; Minajigi et al., 2015). We combined the MEF Hi-C datasets using iterative mapping (Imakaev et al., 2012; Experimental Procedures), identifying 1.3 million Hi-C interactions at 10-kb resolution, of which around 100,000 linked RefSeq-annotated transcription start sites (TSSs) to remote, mostly non-TSS, regions containing DNase I HS (Figure 2C).

Using principal component analysis (PCA) (Lieberman-Aiden et al., 2009; Experimental Procedures), we partitioned the genome into regions of preferential Hi-C interaction (Figure 2D;

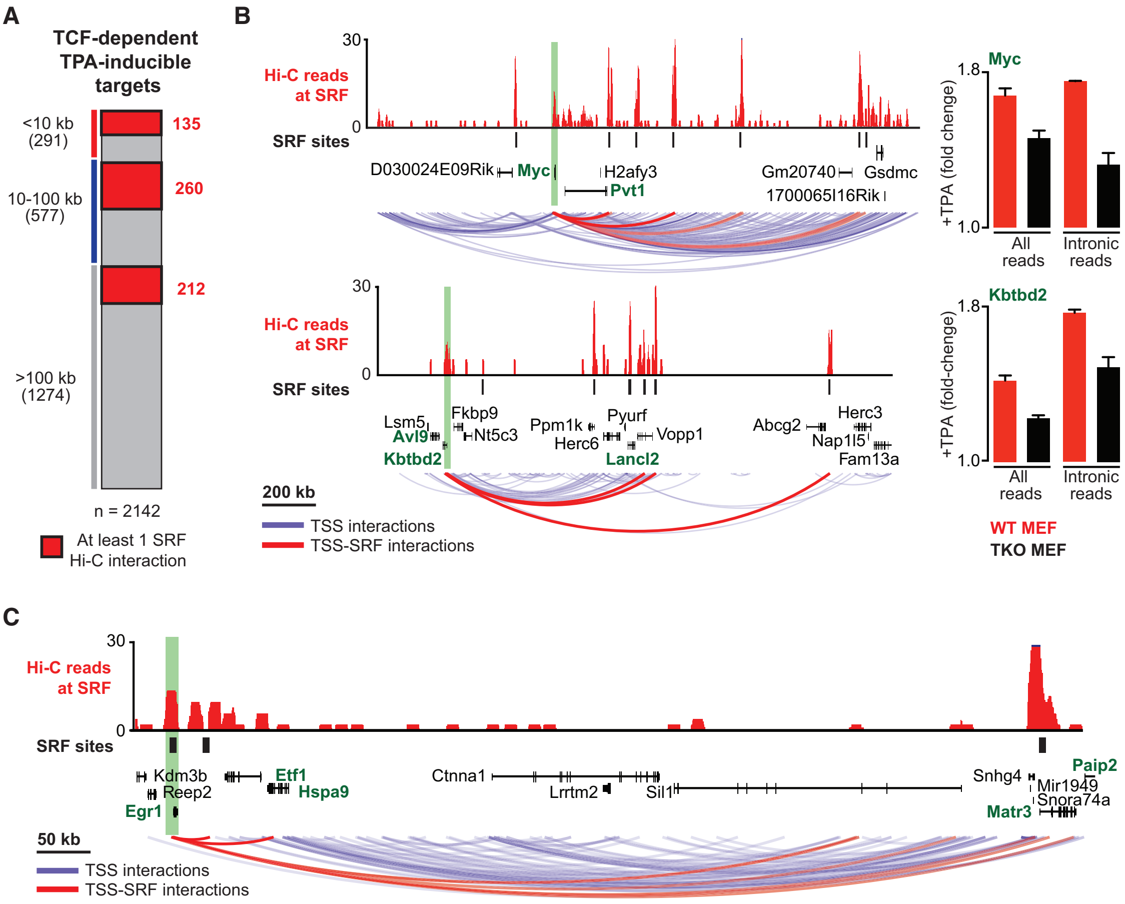

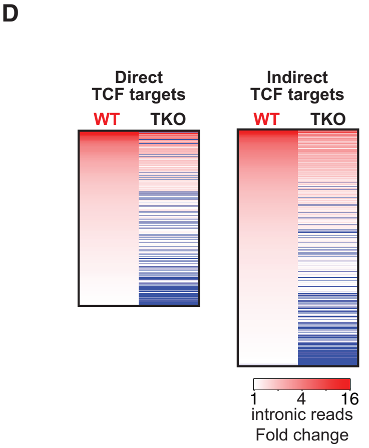

E

Hallmark signatures	Direct adj p-val	Direct n	Indirect adj p-val	Indirect n
TNFA SIGNALING VIA NFKB	3.5E-23	43	2.6E-12	36
APOPTOSIS	1.5E-06	20	3.1E-03	18
PI3K AKT MTOR SIGNALING	1.1E-05	15	2.8E-03	14
MYC TARGETS V1	9.6E-05	20	1.9E-07	29
TGF BETA SIGNALING	4.1E-04	9	ns	-
HYPOXIA	9.3E-04	18	ns	-
MTORC1 SIGNALING	8.4E-03	16	ns	-
DNA REPAIR	8.8E-03	13	ns	-
IL2 STAT5 SIGNALING	9.5E-03	16	ns	-
INFLAMMATORY RESPONSE	0.01	16	3.2E-04	23
IL6 JAK STAT3 SIGNALING	0.02	9	9.6E-04	13
INTERFERON GAMMA RESPONSE	ns	-	8.8E-06	25
ALLOGRAFT REJECTION	ns	-	1.0E-04	23
COMPLEMENT	ns	-	1.0E-03	21
UNFOLDED PROTEIN RESPONSE	ns	-	1.8E-03	15
MYC TARGETS V2	ns	-	2.2E-03	10
UV RESPONSE UP	ns	-	6.3E-03	17

Figure 3. Genome-wide Identification of Direct SRF/TCF Target Genes

(A) Definition of the TCF-dependent TPA-inducible gene set. The integrated SRF ChIP-seq Hi-C data are summarized according to the distance between inducible TSSs to the closest SRF site, with those TSSs and SRF sites displaying Hi-C interaction shaded in red (not all interactions within 10 kb of TSSs were detectable by Hi-C analysis). The 763 Direct TCF-SRF target genes are defined as those whose TSSs are within 10 kb of an SRF site or that interact with one at any distance, as judged by Hi-C. 1,062 Indirect TCF-dependent target genes are defined as those whose TSSs are >100 kb from an SRF site and exhibit no Hi-C interaction with one.

(B) Remote-controlled SRF targets. The *Myc* and *Kbtbd2* loci are shown with Hi-C paired-end reads (10 kb bins) with ends mapping to SRF binding sites shown in red. SRF ChIP-seq binding sites and RefSeq-annotated genes are shown below, with TPA-induced genes shown in green. Significant Hi-C interactions by the *Myc* and *Kbtbd2* TSS are shown in blue, with those involving SRF sites shown in red. The effect of TCF inactivation on RNA-seq reads is shown at the right.

(C) Interactions between the TCF-SRF direct target gene *Egr1* and remote SRF sites identified by Hi-C, displayed as in (B).

(legend continued on next page)

Figures S2G and S2H). These regions, denoted A and B according to PCA score, encompass the topologically associating domains (TADs) defined previously in MEFs (Battulin et al., 2015) and are analogous to the "A and B compartments" defined previously at 1-Mb resolution (Lieberman-Aiden et al., 2009). The A regions correlated strongly with the presence of genes and with overall gene expression (Figure S2I). They also showed significant correlation with DNase I HS frequency, an independent indicator of gene activity (Figure S2J). Most importantly, the A regions contained over 80% of the SRF binding sites (2,137 of 2,577; Figure 2E; Figure S2K). Most TSSs linked to remote SRF binding sites by Hi-C were transcriptionally active, and most TPA-inducible TSSs linked to remote sites were also TCF-dependent (Figure 2E; Table S1), suggesting that the integrated SRF ChIP-seq/Hi-C method identifies functionally relevant TSS-SRF interactions.

Definition of TCF-Dependent TPA-Induced SRF Target Genes

Of the 3,470 TPA-inducible genes, 1,231 had TSSs within 10 kb of an SRF site and/or interacted with a remote SRF site in Hi-C (Figure S3A). Activation of 763 was TCF-dependent, so we defined them as high-confidence Direct TCF-SRF targets (Figure 3A; Table S1). The majority of these (472 of 763) had closest interacting SRF sites >10 kb distant, in many cases >100 kb away, and many exhibited multiple long-distance interactions with SRF, including *Kbtb2* and *Myc*, which interacts with five remote SRF sites, the closest at ~200 kb (Figures 3B and 3C; Table S1; Discussion). TPA-induced chromatin modification at the Myc TSS region is also TCF-dependent (data not shown; C.E., F.G., S.H., A.S., N.M., R.T., G. Kelly, and P. East, unpublished data). The *Egr1* TSS region, which contains multiple SRF sites, also interacted with a putative remote SRF-linked enhancer, the TPA-inducible TCF-dependent *Etf1* gene ~70 kb downstream, and other more distant genes (Figure 3C). In all, 174 "remote-controlled" TSSs were linked to SRF sites that were themselves close to TSSs, blurring the distinction between "enhancer" and "promoter" elements (Figures 3B and 3C; Table S1).

The integrated Hi-C/ChIP-seq data allowed definition of a high-confidence set of 1,062 indirectly TCF-dependent TPA-inducible genes that have TSSs that are neither near SRF sites nor physically interact with them in Hi-C (Figure 3D; see below). Gene set enrichment analysis using the MsigDB database showed that the TCF-dependent Direct and Indirect gene sets were related but distinct (Figure 3E; Tables S4A and S4B). Similar to the TPA-induced population as a whole, Direct genes were enriched in gene hallmarks involved in the cell cycle, signaling networks, and particular transcription factors (Table S4A) and GO categories related to signaling, metabolism, the cell cycle, and the cytoskeleton (Table S4B).

Interestingly, the TCF-dependent Direct gene set contained 100 genes shown previously to be MRTF-SRF targets in serum-stimulated NIH 3T3 fibroblasts (Esnault et al., 2014; Figure S3A; Table S1). This "shared MRTF-TCF" gene set was enriched in gene hallmarks and GO terms involving the cytoskeleton, whereas the remaining "TCF-only" gene set was enriched in proliferative and signaling gene hallmarks and GO terms (Tables S4A and S4B). Finally, approximately 30% of genes whose basal expression was decreased upon TCF inactivation were also physically linked or close to SRF binding sites (1,501 of 5,113; Figure S3B), suggesting that they respond directly to basal levels of TCF activity. The role of the TCFs in the regulation of genes exhibiting enhanced basal activity in TKO MEFs will be considered below.

Elk-1 Re-expression Partially Restores Regulation to TCF-Dependent Target Genes

To assess to what extent TPA-induced gene expression reflects the action of the ERK-regulated TCF activation domain, we reconstituted TKO MEFs with derivatives of the human Elk-1 TCF (Figure S4A). Re-expression of wild-type Elk-1 but not the transcriptionally inactive mutants Elk-1^{nonA} and Elk-$1^{\Delta FW}$ (Balamotis et al., 2009; Buchwalter et al., 2004, 2005) restored significant TPA-inducibility to many Direct and Indirect TCF target genes (Figures 4A and 4B; Table S5). Approximately half of the TPA-inducible TCF-dependent genes (1,016 of 2,142) appeared to be Elk-1 responsive (Figure 4B; Figure S4B). Elk-1 expression preferentially restored the activity of Direct TCF targets, and left TCF-independent genes unaffected (Figure 4C; Figure S4B). Elk-1-responsive genes were enriched for gene hallmarks and GO terms associated with proliferation, cell-cycle control, chromosomal replication, and segregation (Tables S4A and S4B), although Elk-1 expression failed to restore normal proliferation to reconstituted TKO MEFs (Figure 4D). Thus Elk-1, and presumably the other TCFs, is directly involved in control of proliferative gene expression.

The Elk-1 TCF Functions with SRF in the TPA Response

To gain insight into the extent to which Elk-1's activity is SRF-dependent as opposed to autonomous (Boros et al., 2009a, 2009b), we carried out ChIP-seq in reconstituted TKO MEFs (Experimental Procedures). We identified 336 high-confidence Elk-1 binding sites associated with CArG and ETS motifs, bound equally well by the Elk-1^{nonA} and Elk-$1^{\Delta FW}$ mutants, and unaffected by TPA stimulation (Figure 4E; Figure S4D, Table S3). Of these Elk-1 sites, 251 coincided with SRF peaks, whereas 85 were apparently SRF-independent "solo" sites (Figure 4F). In general, the SRF-associated Elk-1 peaks coincided with DNase I HS and were located proximal to TSSs, whereas the solo Elk-1 sites were not (Figures S4F and S4G; Table S3).

Approximately 25% of the detectable SRF/Elk-1 binding sites (89 of 356) were associated with the Direct TCF-SRF target genes, and a further 30 were associated with TPA-inducible SRF target genes that were not scored as TCF-dependent (Figure S3B; Table S3). In contrast, only 2.5% of Elk-1 solo binding

(D) TPA induction is similar at both Direct and Indirect TCF target genes, as assessed by the fold change in intronic RNA-seq reads.
(E) The relation between Direct and Indirect TCF target genes and MsigDB hallmark gene set signatures was assessed by hypergeometrical test (the number of genes and the Bonferroni-adjusted p value are shown).
See also Figure S3 and Tables S3 and S4.

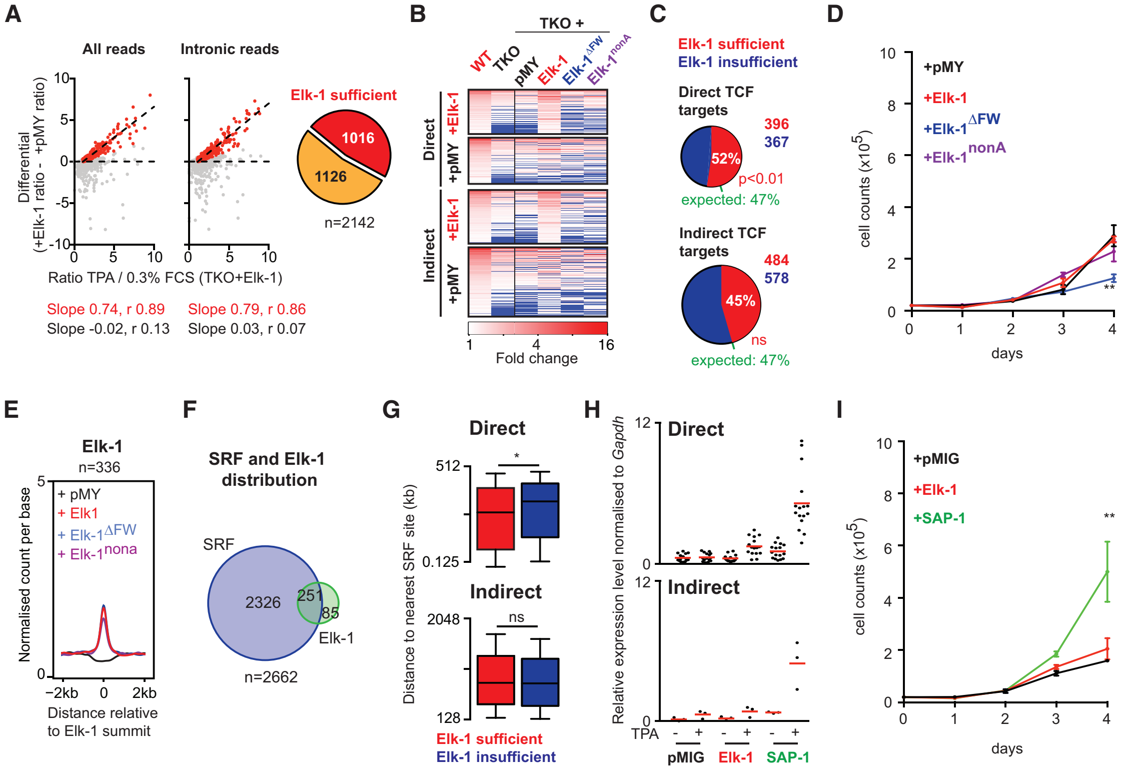

Figure 4. Elk-1 Acts with SRF to Restore TCF-Dependent Gene Expression in TKO MEFs

(A) Identification of genes whose induction in TKO MEFs is restored by expression of wild-type human Elk-1. The plots compare the TPA induction ratio in TKO MEFs with and without Elk-1 expression for both total and intronic RNA-seq reads. Genes whose induction is restored by Elk-1 (red) exhibit a systematic relationship between their inducibility in the two contexts whereas others (gray) do not; slope and Spearman r are indicated. Right: summary.

(B) Heatmap representation of TPA-induced gene expression (fold change over baseline) in wild-type and TKO MEFs and TKO MEFs expressing the indicated Elk-1 derivatives. Genes whose regulation is restored by wild-type Elk-1 are shown, divided into Direct and Indirect categories. The plot is ranked by the magnitude of the TPA-induced changes in wild-type MEFs.

(C) Proportions of Direct and Indirect TCF target genes whose regulation by TPA is restored by Elk-1 expression. Restoration of Direct, but not Indirect, targets is significantly above the 47% expected from the overall restoration of TCF targets (two-tailed binomial test, $^*p < 0.05$).

(D) Expression of human does not restore the proliferation defect observed in TKO MEFs. Cell counts for TKO MEFs expressing the indicated proteins were recorded over 4 days.

(E) Metaprofiles showing the average normalized count per base across the 336 Elk-1 binding sites identified by ChIP-seq in TKO MEFs reconstituted with Elk-1 derivatives as indicated.

(F) Coincidence between SRF and Elk-1 ChIP-seq peaks.

(G) Direct TCF-SRF targets whose regulation is restored by Elk-1 expression are closer to SRF sites than those whose regulation is not. Mann-Whitney test, $^*p < 0.05$.

(H) Elk-1 transactivation is weaker than that of SAP-1. TPA induction of 16 direct TCF targets (*Egr1*, *Egr2*, *Egr3*, *Ier2*, *Per1*, *Junb*, *Fos*, *Zfp36*, *Dnajb1*, *Arl5*, *Pitpna*, *Mob3a*, *Pramef8*, *Tada3*, *Argap1*, and *Arpc4)* and three indirect targets (*Ier3*, *Klf10*, and *Cxcl1*) was analyzed by qRT-PCR.

(I) SAP-1 expression restores proliferation in TKO MEFs. Cells were counted over 4 days. Two-way ANOVA, $^{**}p < 0.01$.

See also Figure S4 and Tables S5 and S6.

sites (9 of 356) were associated with TPA-inducible genes (Table S4A). Elk-1 re-expression in TKO MEFs restored TPA inducibility to genes associated with SRF/Elk-1 sites (94 of 151) or solo Elk-1 sites (3 of 9; *Abce1*, *Trpc7*, and *Hat1*) (Table S5). Thus, Elk-1 acts predominantly in partnership with SRF to control TPA-inducible gene expression.

Next we sought to understand why Elk-1 expression only partially restored TCF-dependent TPA induction. Although it cannot be ruled out that this reflects gene-specific targeting of Elk-1, we found that the "rescued" genes had TSSs that were in general significantly closer to SRF sites than genes that were refractory to Elk-1 expression (Figure 4G). Previous studies have suggested that strong transcriptional activators work more effectively over distance than weak ones (Carey et al., 1990). We therefore compared the strength of transcription activation by Elk-1 with that of its relative SAP-1, with which it is functionally redundant (Costello et al., 2010). SAP-1 was significantly more effective than Elk-1 in restoring TPA-inducibility to 19 selected TCF-SRF target genes in TKO MEFs (Figure 4H; Figure S4A) and also substantially enhanced TKO MEF proliferation (Figure 4I). Taken together, these results suggest that the partial rescue of TCF-SRF target genes by Elk-1 expression in TKO

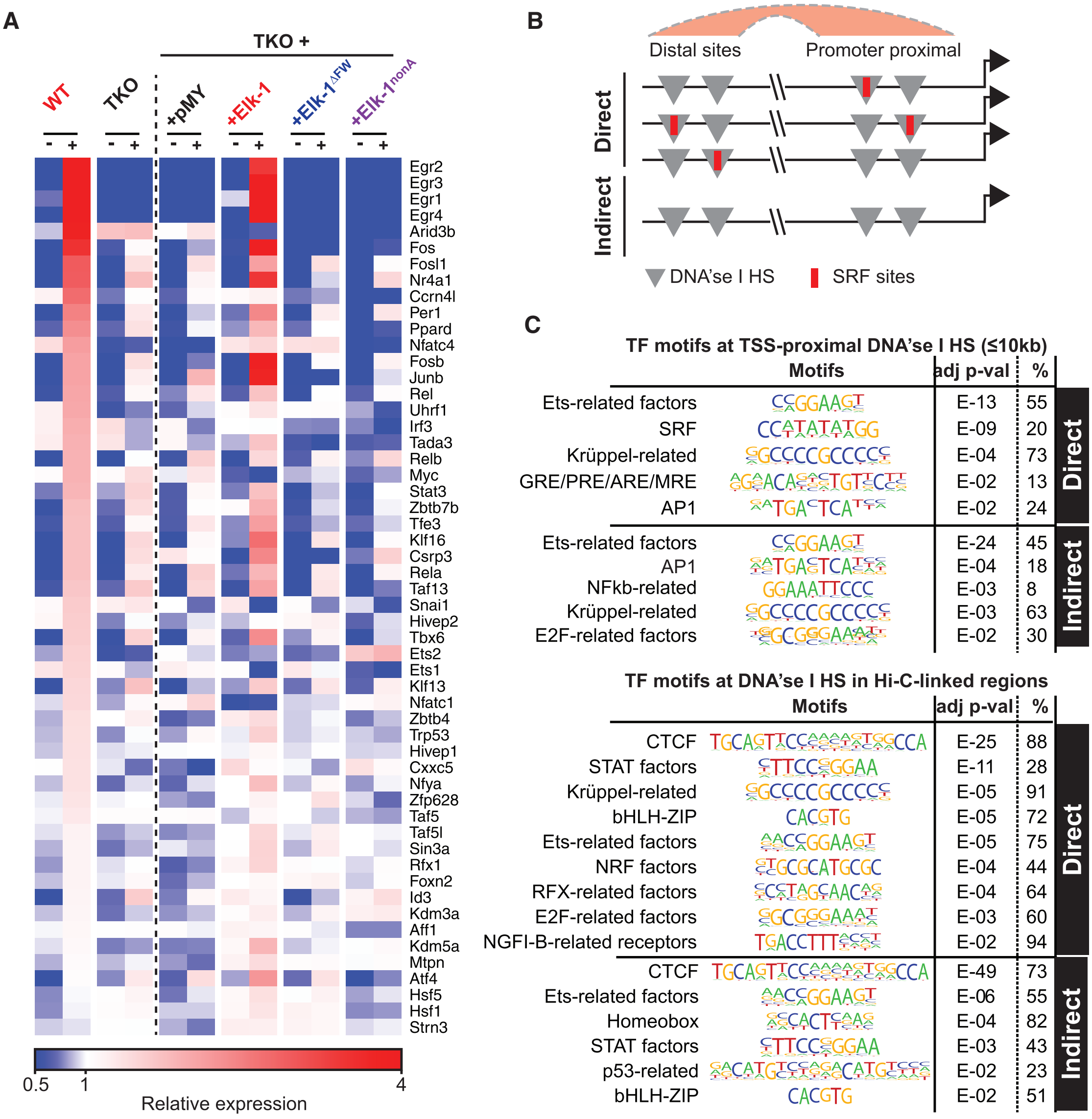

Figure 5. Indirect and Direct TCF-Dependent Target Genes Are Linked by Transcriptional Regulation

(A) Heatmap representation of TPA-induced transcription of 54 transcription factor genes from the Direct TCF target gene set, ranked by magnitude of the TPA-induced changes in wild-type MEFs.

(B) Potential relationships between DNase I HS (gray) and SRF binding sites (red) at TSS-proximal or remote regulatory sites of Direct and Indirect TCF target genes, with Hi-C linkages indicated by the crescent.

(C) TF motifs occurring in putative regulatory regions (DNase I HS summit ± 100 bp) of Direct and Indirect TCF target genes. Motifs scoring as significantly enriched relative to their representation across all DNase I HS-associated sequences are shown together with their frequency. CTCF was recovered at all distal motifs, presumably reflecting its association with the insulator elements that define compartment boundaries.

See also Table S6.

MEFs at least partly reflects its relatively weak transcriptional activation capacity.

Indirect and Direct TCF-Dependent Target Genes Are Linked through Transcriptional Regulation

The expression of Indirect TCF-dependent target genes must either rely on basal levels of proteins encoded by direct TCF-SRF target genes, or reflect a secondary response to transcriptional regulators produced by immediate-early genes. The Direct TCF-SRF target gene set includes 54 different transcription factors, many of which are themselves sensitive to ERK activation (for references, see Table S6). These include members of the Egr, AP-1, Ets, NF-κB, and STAT families in addition to Myc, p53, and others (Figure 5A), and the Direct gene set is indeed enriched in gene hallmarks associated with these transcription factors (Tables S4A and S4B).

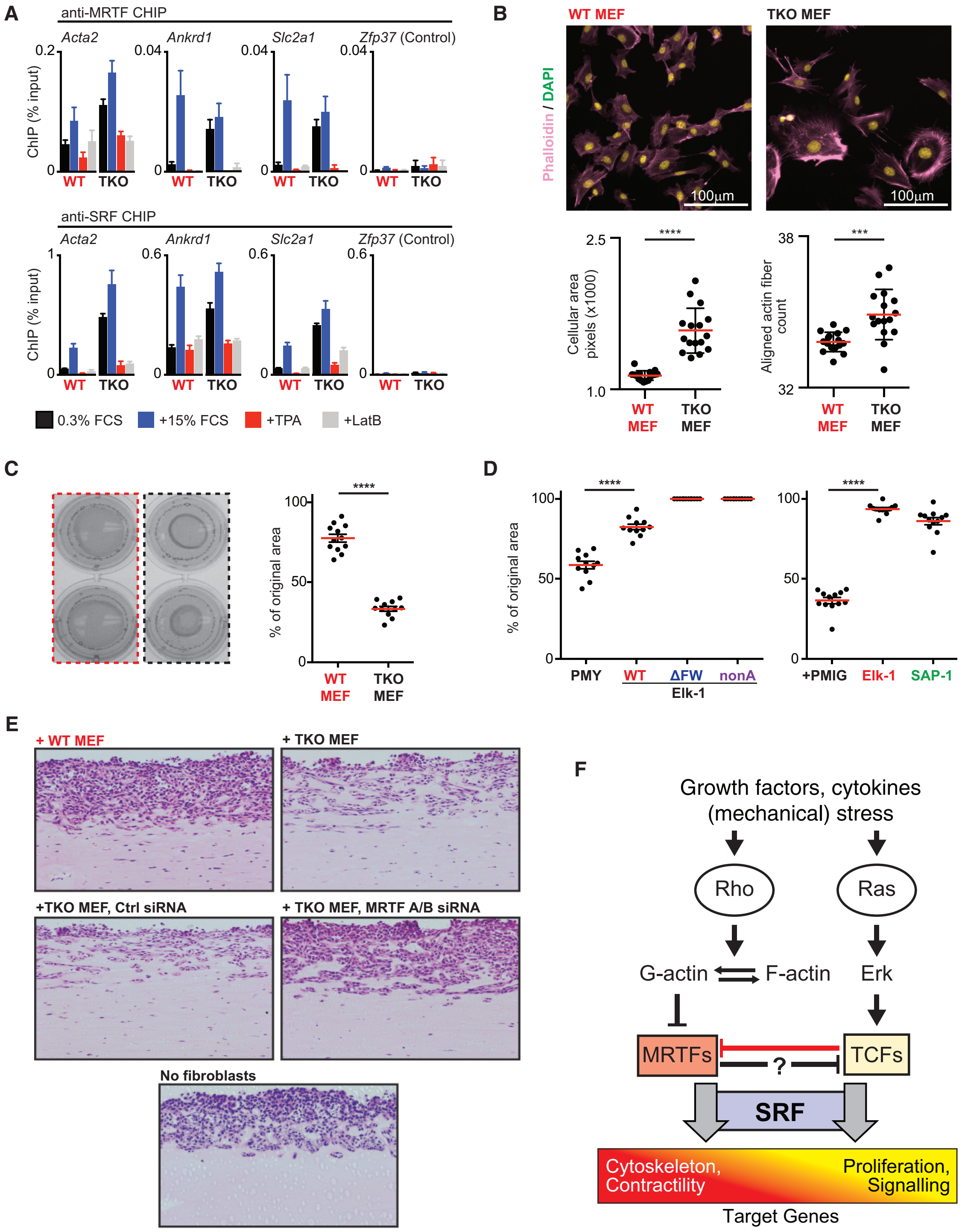

Figure 6. TCF Binding Directly Antagonizes MRTF-Dependent Gene Expression

(A) Quantitative ChIP analysis of MRTF-A and SRF binding at regulatory elements of *Acta2*, *Ankrd1*, *Slc2a1*, and *Zfp37* negative control in wild-type and TKO MEFs either resting (0.3% FCS, black) or treated with serum (30 min, blue), TPA (30 min, black), or Latrunculin-B (5 min, gray). Error bars show SEM (n = 4).

(B) TKO MEFs are large and contain more aligned actin fibers. Cells were stained for F-actin (Phalloidin, magenta) and DNA (DAPI, blue), and the cellular area and aligned actin fibers were quantified (n = 16, >3,000 cells/condition). Read line, mean per set of replicates. (****p < 0.0001, ***p < 0.001, Mann-Whitney test).

(C) TKO MEFs are highly contractile. Left: representative replicate gel contraction wells containing wild-type (red) or TKO (black) MEFs. Right: quantitation (n = 12; red bar, mean; ****p < 0.0001; Mann-Whitney test).

(legend continued on next page)

To investigate the relation between Indirect and Direct TCF target gene regulation, we examined their potential regulatory sequences. Many of these are likely to coincide with DNase I HS, so we performed HOMER motif analysis on all DNase I HS sequences located within 10 kb of or linked to Indirect TSSs by Hi-C, scoring only motifs over-represented with respect to the entire DNase I HS sequence population (Figures 5B and 5C). At TCF targets, TSS-proximal sites were enriched in SRF, Ets, AP-1, NF-κB, Kruppel-related (i.e., Egr-like), and E2F motifs, whereas distal sites were associated with Ets, Homeobox, STAT, p53, and E-BOX motifs; as expected, the SRF consensus was not enriched at Indirect targets (Figure 5C). These data support the view that products of Direct TCF-SRF target genes are required for the response of Indirect TCF targets to TPA stimulation.

TCF Binding Antagonizes MRTF-SRF Signaling

We saw above that many genes exhibit increased basal transcription in TKO MEFs (Figure 1D; Table S1), including hundreds previously identified as MRTF-SRF target genes in NIH 3T3 cells (Esnault et al., 2014). Indeed, in TKO MEFs, the enhanced transcription of MRTF-SRF targets such as *Acta2*, *Ctgf*, and *Dstn* was inhibited by Latrunculin B (LatB), which inhibits MRTF (Miralles et al., 2003; Figure S5A). Moreover, quantitative ChIP showed substantially increased basal levels of MRTF recruitment to SRF targets such as *Acta2*, *Ankrd1*, and *Slc2a1,* which was inhibited by LatB or TPA, which inhibit Rho-actin signaling (Miralles et al., 2003), but further increased by serum stimulation (Figure 6A; Figure S5A). TKO MEFs also exhibited increased MRTF access to TCF-SRF direct target IE promoters in wild-type cells (Figure S5B). Thus, TCF inactivation potentiates MRTF-SRF signaling.

Finally we investigated the functional consequences of the increased MRTF-SRF signaling in TKO MEFs. TKO MEFs were larger than wild-type cells, contained increased numbers of parallel F-actin fibers (Figure 6B), and were significantly more contractile than wild-type MEFs, as assessed by the ability to contract collagen gel (Figure 6C). Re-expression of wild-type or transcriptionally inactive Elk-1 derivatives, or wild-type SAP-1 effectively suppressed the hypercontractile phenotype (Figure 6D). Pro-invasive behavior is associated with hypercontractility in cancer-associated fibroblasts (Calvo et al., 2013; Gaggioli et al., 2007). Accordingly, TKO MEFs, but not wild-type MEFs, strongly promoted invasion of 4T1 breast carcinoma cells in organotypic culture, and this was inhibited by small interfering RNA (siRNA)-mediated MRTF depletion (Figure 6E; Figure S5C). In sum, these data show that the TCFs generally inhibit MRTF-SRF signaling and that this reflects direct competition with the MRTFs for access to SRF, rather than the TCFs' ability to induce gene expression (Discussion). As a result, TCF-MRTF antagonism can contribute to control of "activated" fibroblast phenotypes, as seen in carcinoma-associated fibroblasts (Figure 6F; Discussion).

DISCUSSION

Most TPA-Induced Gene Activation Is TCF-Dependent

The ERK-regulated TCF family of SRF partner proteins were first identified as regulators of the *Fos* gene (Buchwalter et al., 2004; Shaw et al., 1989), but the extent of their role in immediate-early gene expression and its significance for cell proliferation have been unclear. We found that TPA-induced genes in MEFs are enriched for gene hallmarks and GO terms associated with signal transduction, metabolism, transcription, and proliferation. Over 60% of TPA-induced gene expression in MEFs was TCF-dependent, and the proliferation of TCF-deficient MEFs was impaired. Previous studies have implicated the TCFs in control proliferation in response to adhesive and oncogenic stimuli (Vickers et al., 2004; Wozniak et al., 2012; Yang et al., 2012), and our results show that TCF-SRF signaling controls a gene expression program governing proliferation. We also found that TPA significantly downregulated many MRTF-SRF-controlled genes, consistent with the finding that TPA also downregulates Rho (Panayiotou et al., 2016).

The Role of the TCFs in TPA-Induced Transcriptional Activation

We used an integrated SRF ChIP-seq/Hi-C approach to define a set of 763 TPA-induced TCF-SRF Direct target genes, whose TSSs are in close proximity to and/or physically linked to SRF binding sites. The Direct TCF-dependent gene signature provides a picture of the acute transcriptional response to ERK activation and, like the TPA-induced signature as a whole, it is significantly enriched in genes involved in signaling, transcription, and proliferation. Regulated expression of many Direct TCF-SRF target genes in TCF-deficient MEFs in TKO cells could be restored by re-expression of human Elk-1. TCF activity also promoted basal transcription of many genes in MEFs, whether TPA-inducible or not. This might reflect stochastic pulses of ERK activation that occur in many cell types (Aoki et al., 2013).

MRTF-SRF signaling regulates target genes involved in cytoskeletal dynamics (Esnault et al., 2014; Medjkane et al., 2009; Olson and Nordheim, 2010; Schratt et al., 2002), and, interestingly, numerous members of the Direct TCF-SRF-dependent gene set were defined previously as MRTF-SRF targets in serum-stimulated NIH 3T3 cells (Esnault et al., 2014). Thus, at a significant number of genes, both cofactor families can variably access SRF according to cellular context (Figure 6F). Our results imply that the relative levels of TCF and MRTF proteins in different biological contexts can affect the balance between

(D) TKO MEF hypercontractility is suppressed by expression of either wild-type Elk-1, its two transcriptionally inactive derivatives Elk-1^{FW} and Elk-1^{nonA}, or SAP-1. Quantitation is as in (C).

(E) Representative images showing the invasion of 4T1 breast carcinoma cells into Matrigel containing no added fibroblasts, wild-type MEFs, or TKO MEFs with or without MRTF-A/B or control siRNAs.

(F) Direct competition model for antagonism between MRTF-SRF- and TCF-SRF-dependent gene expression programs. Competition between the pathways for shared targets is indicated by diagonal shading.

See also Figure S5.

proliferative and cytoskeletal gene expression programs (Figure 6F); this is discussed further below. The Yap-Taz proteins are also implicated in the control of both proliferative and cytoskeletal gene expression, acting at least in part through long-range TEAD/AP-1 elements (Dupont et al., 2011; Zanconato et al., 2015). Several AP-1 components are encoded by SRF target genes, providing a potential mechanism by which the two pathways might converge.

We found that TCF-SRF signaling contributes to TPA-induced *Myc* transcription and identified five distant potential regulatory SRF sites. *Myc* was first identified as a mitogen-inducible gene over 30 years ago (Greenberg and Ziff, 1984; Kelly et al., 1983), and its regulation remains poorly understood, but our findings identify a regulatory input for ERK signaling (Kerkhoff et al., 1998). The E box Myc binding consensus is enriched at putative regulatory sequences of genes that are indirectly TCF-responsive, consistent with *Myc* playing a role in the secondary response to growth factor stimulation. In spite of this, *Myc* cooperates with *Ras* in transformation, perhaps reflecting downregulation of ERK by chronic Ras activation.

Previous work has shown that the TCFs can regulate transcription independently of SRF (Boros et al., 2009a, 2009b; Buchwalter et al., 2005), but Elk-1 predominantly acted through SRF in our system. The ability of Elk-1 to restore TPA-inducible transcription in TKO MEFs required its transcriptional activation domain, indicating that Elk-1 acts by providing a primary signal input for activation. Nevertheless, Elk-1 failed to restore the TPA-induced transcriptional response at all TCF-dependent direct SRF targets; it also restored chromatin modifications at only a subset of TCF-dependent SRF target TSSs (C.E., F.G., S.H., A.S., N.M., R.T., G. Kelly, and P. East, unpublished data). Although it is likely that this reflects TCF-specific gene targeting, our results suggest that the strength of transcription activation by the different TCFs may also play a role. In a direct comparison, SAP-1 both activated transcription and restored proliferation more effectively than Elk-1, suggesting that it may restore a greater fraction of TCF-dependent gene expression. However, expression of mouse Elk-1 was also more effective than human Elk-1 in restoring proliferation (Mylona et al., 2016). Resolution of these issues will require direct comparative analysis of TCF recruitment with genomic targets.

TCF Target Gene Identification by Integrated ChIP-Seq/Hi-C

We identified over 700 TCF-dependent direct SRF target genes by integrating ChIP-seq data from unstimulated MEFs with published MEF Hi-C datasets (Battulin et al., 2015; Minajigi et al., 2015), an approach used previously to identify NF-κB target TSSs (Jin et al., 2013). Although this approach uses the stringent criterion of direct TF-TSS physical interaction to identify candidate TF targets, because of limitations of the Hi-C data it is likely to under- rather than over-estimate the number of true targets. We note, however, that many SRF-interacting TSSs remained unaffected by TPA stimulation and must be somehow refractory to SRF-linked signals, as observed previously (Esnault et al., 2014). Thus, even with an additional functional constraint, establishing SRF-TSS linkage by Hi-C is not sufficient to predict whether a TSS is signal-regulated.

The integrated SRF ChiP-Seq/Hi-C approach also allowed us to define more than 1,000 TPA-inducible genes that, although TCF-dependent, are not direct TCF-SRF targets. Induction of these genes must either be dependent on the basal expression of Direct TCF-SRF target genes or arise as a secondary response to their acute activation. The putative regulatory sequences associated with Indirect TCF-SRF targets are significantly enriched for the binding motifs of transcription factors encoded by Direct TCF-SRF targets, supporting the notion that the IE response is a transcription cascade.

The TCFs Antagonize MRTF-SRF Signaling

Our data show that TCF-MRTF antagonism is a general feature of SRF regulation, reflecting their competition for binding to SRF, to which their binding is mutually exclusive (Miralles et al., 2003; Wang et al., 2004; Zaromytidou et al., 2006; Figure 6F). In fibroblasts, TCF-MRTF competition at many SRF sites potentially influences the "activation" state by controlling contractility and pro-invasive behavior. Nevertheless, many SRF target genes appear to be coupled predominantly to one pathway or the other (Gineitis and Treisman, 2001; Sotiropoulos et al., 1999). This preference is likely determined by the quality of the SRF binding site. TCF binds preferentially to sites with well-defined Ets motifs (Treisman et al., 1992), whereas MRTF-SRF interaction is favored at sites that can be easily bent (Zaromytidou et al., 2006). Indirect MRTF-TCF antagonism can also occur, however, as in the control of ERK signaling by the MRTF target gene Mig6 (Descot et al., 2009).

Our data suggest a model in which the pro-proliferative effects of TCF-SRF signaling and increased contractility brought about by MRTF-SRF signaling are mutually antagonistic. In smooth muscle cells, PDGF stimulation induces exchange of the Elk-1 TCF for myocardin (Wang et al., 2004), indicating that this antagonism may itself be influenced by signaling. Although TCF DNA binding activity is not influenced by signal strength, nuclear MRTF levels in resting cells are controlled by cellular G-actin concentration (Miralles et al., 2003; Vartiainen et al., 2007). Thus, TCF-MRTF competition will also be affected by the basal level of Rho signaling, and factors affecting the degree to which a particular stimulus activates Rho- and ERK-dependent pathways will therefore also influence SRF transcriptional outputs (Figure 6F). It is tempting to speculate that it is TCF-MRTF competition that has provided the selective pressure to maintain the single SRF gene during metazoan evolution.

EXPERIMENTAL PROCEDURES

Cells

Wild-type or Elk1$^{-/-}$ Elk3$^{\delta/\delta}$ Elk4$^{-/-}$ mouse embryo fibroblasts (Costello et al., 2010) were immortalized by expression of SV40 large T. For stimulation with 50 ng/ml TPA, MEFs were maintained in 0.3% FCS. MEFs were reconstituted with wild-type human Elk-1 and SAP1 and Elk-1^{nonA} and Elk-1$^{\Delta FW}$ (Cruzalegui et al., 1999; Marais et al., 1993; Price et al., 1995) by retroviral transduction using standard procedures. Pools of MEFs expressing TCFs at similar levels were derived by fluorescence-activated cell sorting (FACS) for GFP marker expression. Fibroblast organotypic cultures and force-mediated matrix remodeling assays were as described previously (Calvo et al., 2013). High-throughput imaging was used to quantify actin stress fiber length using the Cellomics ArrayScan VTI and compartmental analysis (BioApplications; 3,000 cells/point).

ChIP and RNA Analysis

ChIP and ChIP-seq have been described previously (Esnault et al., 2014; Miralles et al., 2003) with sequencing on the Hi-seq2500 (150-base read lengths). Antibodies used were as follows: SRF (sc-335, lot no. D3013, Santa Cruz Biotechnology) and MRTF-A (sc-21558, lot no. I1412, Santa Cruz); anti-mouse Elk-1, amino acids (aa) 309–429, was made in-house (Costello et al., 2010) and affinity-purified against recombinant human Elk-1^{nonA} (aa 309–429). qPCR gene expression analysis was by standard methods. For primers, see Supplemental Information.

RNA-Seq

Samples were prepared using the GenElute mammalian total RNA miniprep kit (RTN350-1KT). DNA was removed by DNAase I and rRNA by the Ribo-zero rRNA removal kit (Epicenter). RNA-seq libraries were prepared from 1 μg RNA using the directional mRNA-seq Library Prep v1.0 protocol (Illumina). Libraries were subjected to 72-base single-end sequencing on the Hiseq analyzer, and trimmed to 50 bp. Raw and processed data are in the GEO: GSE75667.

RNA-seq data were aligned to the mm9 mouse genome using Burrows-Wheeler Aligner (BWA) (default settings). Reads within annotated RefSeq genes (“all reads”) and reads containing intronic sequences (“intronic reads”) were annotated using the bam file obtained by BWA as input. Normalization was against a set of invariant genes across samples, defined as those with differences in read counts within 1σ from the mean difference (μ_{diff}), assuming a quasi-normal distribution of gene read counts. Differential gene expression analysis was performed with Deseq (comparing resting and TPA-induced, wild-type, and TKO MEF samples; adjusted p value (adj-p) $\leq$ 0.01; minimum change of 10%). The effects of knockout and reconstituted background were estimated by comparing the degree of gene induction under the different conditions and identifying genes that are similarly affected (Supplemental Information).

ChIP-Seq Analysis

ChIP-seq reads were trimmed to 50 bp and aligned using BWA to the mm9 mouse genome (default settings). Raw and processed data are online in the GEO: GSE75667. Candidate SRF and Elk-1 peaks were identified by Model-based Analysis of ChIP-seq (MACS) (Zhang et al., 2008), retaining those called at p < 1E-4 in at least three pseudo-replicate samples. Those whose read distribution was significantly different from Elk-1 ChIP-seq, performed on TKO MEFs transduced with empty vector, were identified using Deseq (minimal increase 1.5-fold, p $\leq$ 0.05) and retained for further study. This approach has an effectively negligible false discovery rate (FDR). HOMER was used for motif discovery in sequences ± 100 bp from the ChIP-seq peaks or DHS peaks.

Hi-C Analysis

This analysis used the HOMER Hi-C analysis pipeline (http://homer.salk.edu/homer/interactions). Reads from NCBI SRA: SRX554530 (Battulin et al., 2015), and GEO: GSM1648486 and GSM1696042 (Minajigi et al., 2015) were aligned to the mouse genome (Imakaev et al., 2012). A “universe” of ~1.3 million significant interactions at 10-kb-resolution interactions was determined, taking into account linear genomic distance and sequencing depth (p $\leq$ 0.05 and Z score $\geq$ 2). Chromosomal regions exhibiting preferential interactions were identified by using the automated PCA analysis on Hi-C data in HOMER (runHiCpca.pl), retaining those with a PCA score >100. Interactions mapping at all RefSeq-annotated TSSs (~130,000) were retrieved using the annotateInteractions.pl pipeline.

ACCESSION NUMBERS

The accession numbers for the raw sequencing files and the processed data reported in this paper are GEO: GSE75667.

SUPPLEMENTAL INFORMATION

Supplemental Information includes Supplemental Experimental Procedures, six figures, and six tables and can be found with this article online at http://dx.doi.org/10.1016/j.molcel.2016.10.016.

AUTHOR CONTRIBUTIONS

F.G. and C.E. designed, conducted, and interpreted experiments. F.G., S.H., and A.S. analyzed the genomic data and wrote scripts. N.M. performed the sequence analysis. R.T. conceived the project, suggested and interpreted experiments, and wrote the paper with F.G.

ACKNOWLEDGMENTS

We thank Charlie Foster and Erik Sahai for help with the contractility and invasion assays; Mike Howell (Crick high-throughput screening platform) for help with quantitation of fluorescence images; Nick Luscombe, Caroline Hill, Rob Nicolas, and members of the transcription group for reagents; Alfred Nordheim and Boh Wasylyk for Elk1$^{-/-}$ and Elk3$^{\delta/\delta}$ mice; Victoria Lawson for generating MEFs; and the Crick Advanced Sequencing platform for sequencing. Work in the R.T. group was supported by Cancer Research UK until March 31, 2015 and since then by the Francis Crick Institute, which receives its core funding from Cancer Research UK (FC001-190), the UK Medical Research Council (FC001-190), and the Wellcome Trust (FC001-190). This work was also supported by ERC Advanced Grant 268690 ACTINonSRF (to R.T.). C.E. was the recipient of EMBO long-term fellowship ALTF 872-2008.

Received: August 15, 2016
Revised: September 26, 2016
Accepted: October 11, 2016
Published: November 17, 2016

REFERENCES

Aoki, K., Kumagai, Y., Sakurai, A., Komatsu, N., Fujita, Y., Shionyu, C., and Matsuda, M. (2013). Stochastic ERK activation induced by noise and cell-to-cell propagation regulates cell density-dependent proliferation. Mol. Cell *52*, 529–540.

Balamotis, M.A., Pennella, M.A., Stevens, J.L., Wasylyk, B., Belmont, A.S., and Berk, A.J. (2009). Complexity in transcription control at the activation domain-mediator interface. Sci. Signal. *2*, ra20.

Battulin, N., Fishman, V.S., Mazur, A.M., Pomaznoy, M., Khabarova, A.A., Afonnikov, D.A., Prokhortchouk, E.B., and Serov, O.L. (2015). Comparison of the three-dimensional organization of sperm and fibroblast genomes using the Hi-C approach. Genome Biol. *16*, 77.

Boros, J., Donaldson, I.J., O'Donnell, A., Odrowaz, Z.A., Zeef, L., Lupien, M., Meyer, C.A., Liu, X.S., Brown, M., and Sharrocks, A.D. (2009a). Elucidation of the ELK1 target gene network reveals a role in the coordinate regulation of core components of the gene regulation machinery. Genome Res. *19*, 1963–1973.

Boros, J., O'Donnell, A., Donaldson, I.J., Kasza, A., Zeef, L., and Sharrocks, A.D. (2009b). Overlapping promoter targeting by Elk-1 and other divergent ETS-domain transcription factor family members. Nucleic Acids Res. *37*, 7368–7380.

Buchwalter, G., Gross, C., and Wasylyk, B. (2004). Ets ternary complex transcription factors. Gene *324*, 1–14.

Buchwalter, G., Gross, C., and Wasylyk, B. (2005). The ternary complex factor Net regulates cell migration through inhibition of PAI-1 expression. Mol. Cell. Biol. *25*, 10853–10862.

Buffet, C., Catelli, M.G., Hecale-Perlemoine, K., Bricaire, L., Garcia, C., Gallet-Dierick, A., Rodriguez, S., Cormier, F., and Groussin, L. (2015). Dual Specificity Phosphatase 5, a Specific Negative Regulator of ERK Signaling, Is Induced by Serum Response Factor and Elk-1 Transcription Factor. PLoS ONE *10*, e0145484.

Calvo, F., Ege, N., Grande-Garcia, A., Hooper, S., Jenkins, R.P., Chaudhry, S.I., Harrington, K., Williamson, P., Moeendarbary, E., Charras, G., and Sahai, E. (2013). Mechanotransduction and YAP-dependent matrix remodelling is required for the generation and maintenance of cancer-associated fibroblasts. Nat. Cell Biol. *15*, 637–646.

Carey, M., Leatherwood, J., and Ptashne, M. (1990). A potent GAL4 derivative activates transcription at a distance in vitro. Science *247*, 710–712.

Chambard, J.C., Lefloch, R., Pouysségur, J., and Lenormand, P. (2007). ERK implication in cell cycle regulation. Biochim. Biophys. Acta *1773*, 1299–1310.

Cochran, B.H., Zullo, J., Verma, I.M., and Stiles, C.D. (1984). Expression of the c-fos gene and of an fos-related gene is stimulated by platelet-derived growth factor. Science *226*, 1080–1082.

Costello, P., Nicolas, R., Willoughby, J., Wasylyk, B., Nordheim, A., and Treisman, R. (2010). Ternary complex factors SAP-1 and Elk-1, but not net, are functionally equivalent in thymocyte development. J. Immunol. *185*, 1082–1092.

Cruzalegui, F.H., Cano, E., and Treisman, R. (1999). ERK activation induces phosphorylation of Elk-1 at multiple S/T-P motifs to high stoichiometry. Oncogene *18*, 7948–7957.

Descot, A., Hoffmann, R., Shaposhnikov, D., Reschke, M., Ullrich, A., and Posern, G. (2009). Negative regulation of the EGFR-MAPK cascade by actin-MAL-mediated Mig6/Errfi-1 induction. Mol. Cell *35*, 291–304.

Dupont, S., Morsut, L., Aragona, M., Enzo, E., Giulitti, S., Cordenonsi, M., Zanconato, F., Le Digabel, J., Forcato, M., Bicciato, S., et al. (2011). Role of YAP/TAZ in mechanotransduction. Nature *474*, 179–183.

Esnault, C., Stewart, A., Gualdrini, F., East, P., Horswell, S., Matthews, N., and Treisman, R. (2014). Rho-actin signaling to the MRTF coactivators dominates the immediate transcriptional response to serum in fibroblasts. Genes Dev. *28*, 943–958.

Gaggioli, C., Hooper, S., Hidalgo-Carcedo, C., Grosse, R., Marshall, J.F., Harrington, K., and Sahai, E. (2007). Fibroblast-led collective invasion of carcinoma cells with differing roles for RhoGTPases in leading and following cells. Nat. Cell Biol. *9*, 1392–1400.

Gauthier-Rouvière, C., Fernandez, A., and Lamb, N.J. (1990). ras-induced c-fos expression and proliferation in living rat fibroblasts involves C-kinase activation and the serum response element pathway. EMBO J. *9*, 171–180.

Gineitis, D., and Treisman, R. (2001). Differential usage of signal transduction pathways defines two types of serum response factor target gene. J. Biol. Chem. *276*, 24531–24539.

Greenberg, M.E., and Ziff, E.B. (1984). Stimulation of 3T3 cells induces transcription of the c-fos proto-oncogene. Nature *311*, 433–438.

Griner, E.M., and Kazanietz, M.G. (2007). Protein kinase C and other diacylglycerol effectors in cancer. Nat. Rev. Cancer *7*, 281–294.

Imakaev, M., Fudenberg, G., McCord, R.P., Naumova, N., Goloborodko, A., Lajoie, B.R., Dekker, J., and Mirny, L.A. (2012). Iterative correction of Hi-C data reveals hallmarks of chromosome organization. Nat. Methods *9*, 999–1003.

Jin, F., Li, Y., Dixon, J.R., Selvaraj, S., Ye, Z., Lee, A.Y., Yen, C.A., Schmitt, A.D., Espinoza, C.A., and Ren, B. (2013). A high-resolution map of the three-dimensional chromatin interactome in human cells. Nature *503*, 290–294.

Kelly, K., Cochran, B.H., Stiles, C.D., and Leder, P. (1983). Cell-specific regulation of the c-myc gene by lymphocyte mitogens and platelet-derived growth factor. Cell *35*, 603–610.

Kerkhoff, E., Houben, R., Löffler, S., Troppmair, J., Lee, J.E., and Rapp, U.R. (1998). Regulation of c-myc expression by Ras/Raf signalling. Oncogene *16*, 211–216.

Lieberman-Aiden, E., van Berkum, N.L., Williams, L., Imakaev, M., Ragoczy, T., Telling, A., Amit, I., Lajoie, B.R., Sabo, P.J., Dorschner, M.O., et al. (2009). Comprehensive mapping of long-range interactions reveals folding principles of the human genome. Science *326*, 289–293.

Marais, R., Wynne, J., and Treisman, R. (1993). The SRF accessory protein Elk-1 contains a growth factor-regulated transcriptional activation domain. Cell *73*, 381–393.

Medjkane, S., Perez-Sanchez, C., Gaggioli, C., Sahai, E., and Treisman, R. (2009). Myocardin-related transcription factors and SRF are required for cytoskeletal dynamics and experimental metastasis. Nat. Cell Biol. *11*, 257–268.

Minajigi, A., Froberg, J.E., Wei, C., Sunwoo, H., Kesner, B., Colognori, D., Lessing, D., Payer, B., Boukhali, M., Haas, W., and Lee, J.T. (2015). Chromosomes. A comprehensive Xist interactome reveals cohesin repulsion and an RNA-directed chromosome conformation. Science *349*, 349.

Miralles, F., Posern, G., Zaromytidou, A.I., and Treisman, R. (2003). Actin dynamics control SRF activity by regulation of its coactivator MAL. Cell *113*, 329–342.

Mylona, A., Theillet, F.X., Foster, C., Cheng, T.M., Miralles, F., Bates, P.A., Selenko, P., and Treisman, R. (2016). Opposing effects of Elk-1 multisite phosphorylation shape its response to ERK activation. Science *354*, 233–237.

Olson, E.N., and Nordheim, A. (2010). Linking actin dynamics and gene transcription to drive cellular motile functions. Nat. Rev. Mol. Cell Biol. *11*, 353–365.

Panayiotou, R., Miralles, F., Pawlowski, R., Diring, J., Flynn, H.R., Skehel, M., and Treisman, R. (2016). Phosphorylation acts positively and negatively to regulate MRTF-A subcellular localisation and activity. eLife *5*, e15460.

Price, M.A., Rogers, A.E., and Treisman, R. (1995). Comparative analysis of the ternary complex factors Elk-1, SAP-1a and SAP-2 (ERP/NET). EMBO J. *14*, 2589–2601.

Pylayeva-Gupta, Y., Grabocka, E., and Bar-Sagi, D. (2011). RAS oncogenes: weaving a tumorigenic web. Nat. Rev. Cancer *11*, 761–774.

Schratt, G., Weinhold, B., Lundberg, A.S., Schuck, S., Berger, J., Schwarz, H., Weinberg, R.A., Rüther, U., and Nordheim, A. (2001). Serum response factor is required for immediate-early gene activation yet is dispensable for proliferation of embryonic stem cells. Mol. Cell. Biol. *21*, 2933–2943.

Schratt, G., Philippar, U., Berger, J., Schwarz, H., Heidenreich, O., and Nordheim, A. (2002). Serum response factor is crucial for actin cytoskeletal organization and focal adhesion assembly in embryonic stem cells. J. Cell Biol. *156*, 737–750.

Shaw, P.E., Schröter, H., and Nordheim, A. (1989). The ability of a ternary complex to form over the serum response element correlates with serum inducibility of the human c-fos promoter. Cell *56*, 563–572.

Sotiropoulos, A., Gineitis, D., Copeland, J., and Treisman, R. (1999). Signal-regulated activation of serum response factor is mediated by changes in actin dynamics. Cell *98*, 159–169.

Subramanian, A., Tamayo, P., Mootha, V.K., Mukherjee, S., Ebert, B.L., Gillette, M.A., Paulovich, A., Pomeroy, S.L., Golub, T.R., Lander, E.S., and Mesirov, J.P. (2005). Gene set enrichment analysis: a knowledge-based approach for interpreting genome-wide expression profiles. Proc. Natl. Acad. Sci. USA *102*, 15545–15550.

Treisman, R., Marais, R., and Wynne, J. (1992). Spatial flexibility in ternary complexes between SRF and its accessory proteins. EMBO J. *11*, 4631–4640.

Vartiainen, M.K., Guettler, S., Larijani, B., and Treisman, R. (2007). Nuclear actin regulates dynamic subcellular localization and activity of the SRF cofactor MAL. Science *316*, 1749–1752.

Vickers, E.R., Kasza, A., Kurnaz, I.A., Seifert, A., Zeef, L.A., O'donnell, A., Hayes, A., and Sharrocks, A.D. (2004). Ternary complex factor-serum response factor complex-regulated gene activity is required for cellular proliferation and inhibition of apoptotic cell death. Mol. Cell. Biol. *24*, 10340–10351.

Wang, Z., Wang, D.Z., Hockemeyer, D., McAnally, J., Nordheim, A., and Olson, E.N. (2004). Myocardin and ternary complex factors compete for SRF to control smooth muscle gene expression. Nature *428*, 185–189.

Weinl, C., Wasylyk, C., Garcia Garrido, M., Sothilingam, V., Beck, S.C., Riehle, H., Stritt, C., Roux, M.J., Seeliger, M.W., Wasylyk, B., and Nordheim, A. (2014). Elk3 deficiency causes transient impairment in post-natal retinal vascular development and formation of tortuous arteries in adult murine retinae. PLoS ONE *9*, e107048.

Wozniak, M.A., Cheng, C.Q., Shen, C.J., Gao, L., Olarerin-George, A.O., Won, K.J., Hogenesch, J.B., and Chen, C.S. (2012). Adhesion regulates MAP kinase/ternary complex factor exchange to control a proliferative transcriptional switch. Curr. Biol. *22*, 2017–2026.

Yang, X., Zhao, M., Xia, M., Liu, Y., Yan, J., Ji, H., and Wang, G. (2012). Selective requirement for Mediator MED23 in Ras-active lung cancer. Proc. Natl. Acad. Sci. USA *109*, E2813–E2822.

Zanconato, F., Forcato, M., Battilana, G., Azzolin, L., Quaranta, E., Bodega, B., Rosato, A., Bicciato, S., Cordenonsi, M., and Piccolo, S. (2015). Genome-wide association between YAP/TAZ/TEAD and AP-1 at enhancers drives oncogenic growth. Nat. Cell Biol. *17*, 1218–1227.

Zaromytidou, A.I., Miralles, F., and Treisman, R. (2006). MAL and ternary complex factor use different mechanisms to contact a common surface on the serum response factor DNA-binding domain. Mol. Cell. Biol. *26*, 4134–4148.

Zhang, Y., Liu, T., Meyer, C.A., Eeckhoute, J., Johnson, D.S., Bernstein, B.E., Nusbaum, C., Myers, R.M., Brown, M., Li, W., and Liu, X.S. (2008). Model-based analysis of ChIP-Seq (MACS). Genome Biol. *9*, R137.

Molecular Cell, Volume *64*

Supplemental Information

SRF Co-factors Control the Balance between Cell Proliferation and Contractility

Francesco Gualdrini, Cyril Esnault, Stuart Horswell, Aengus Stewart, Nik Matthews, and Richard Treisman

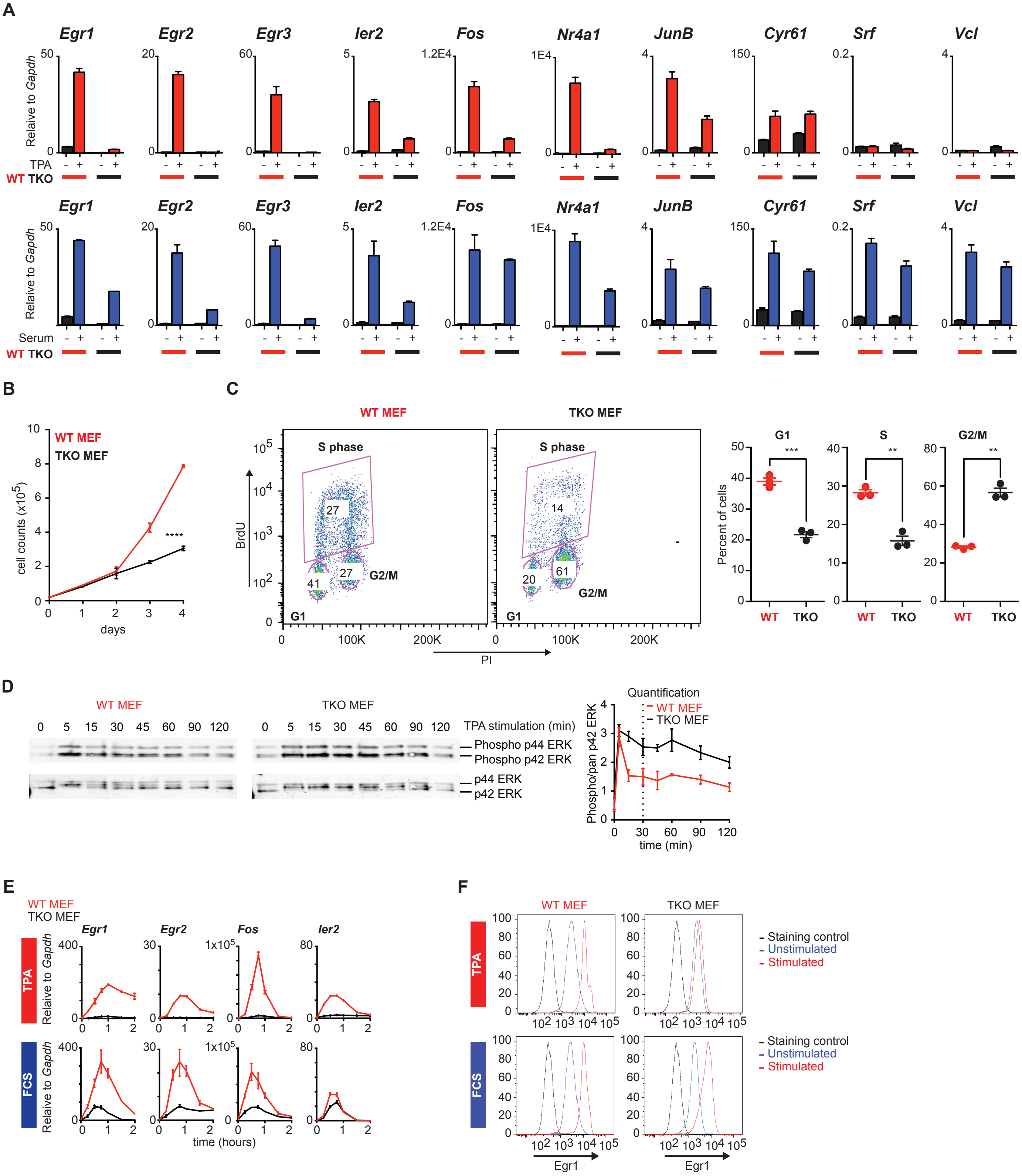
A
Egr1
Egr2
Egr3
Ier2
Fos
Nr4a1
JunB
Cyr61
Srf
Vcl
Relaive to Gapdh
TPA
Serum
WT TKO
B
WT MEF
TKO MEF
cell counts (x10^5)
days
****
C
WT MEF
TKO MEF
S phase
G1
G2/M
BrdU
PI
Percent of cells
G1
S
G2/M
WT
TKO
***
**
D
WT MEF
TKO MEF
TPA stimulation (min)
Phospho p44 ERK
Phospho p42 ERK
p44 ERK
p42 ERK
Quantification
Phospho/pan p42 ERK
time (min)
E
WT MEF
TKO MEF
TPA
FCS
time (hours)
F
Staining control
Unstimulated
Stimulated
Egr1

Figure S1

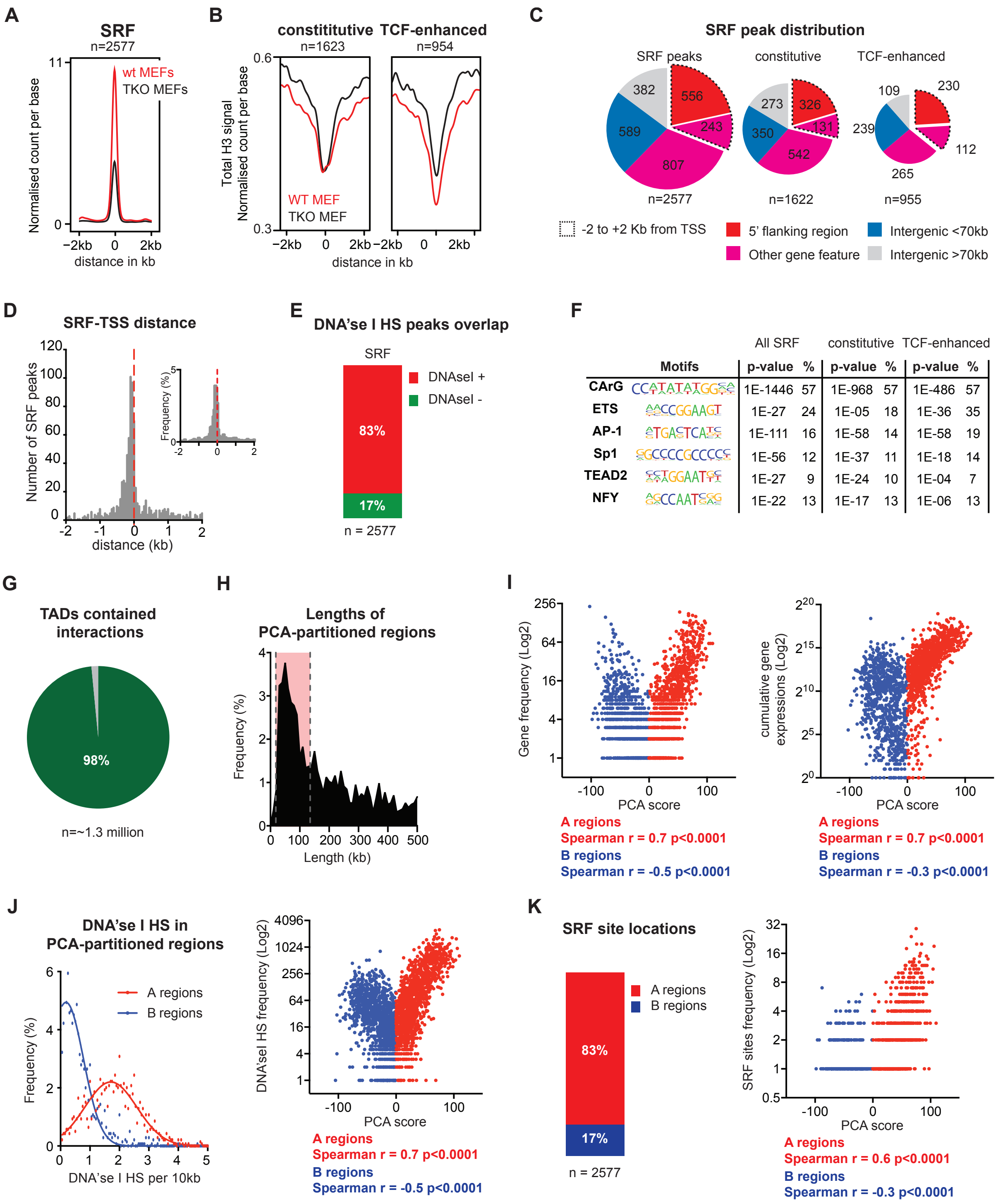
A
SRF
n=2577
wt MEFs
TKO MEFs
Normalised count per base
distance in kb
B
constititutive TCF-enhanced
n=1623
n=954
Total H3 signal
Normalised count per base
WT MEF
TKO MEF
distance in kb
C
SRF peak distribution
SRF peaks
constitutive
TCF-enhanced
n=2577
n=1622
n=955
-2 to +2 Kb from TSS
5' flanking region
Other gene feature
Intergenic <70kb
Intergenic >70kb
D
SRF-TSS distance
Number of SRF peaks
Frequency (%)
distance (kb)
E
DNA'se I HS peaks overlap
SRF
DNAseI +
DNAseI -
83%
17%
n = 2577
F
All SRF
constitutive
TCF-enhanced
Motifs
p-value %
CArG 1E-1446 57 1E-968 57 1E-486 57
ETS 1E-27 24 1E-05 18 1E-36 35
AP-1 1E-111 16 1E-58 14 1E-58 19
Sp1 1E-56 12 1E-37 11 1E-18 14
TEAD2 1E-27 9 1E-24 10 1E-04 7
NFY 1E-22 13 1E-17 13 1E-06 13
G
TADs contained interactions
98%
n=~1.3 million
H
Lengths of PCA-partitioned regions
Frequency (%)
Length (kb)
I
Gene frequency (Log2)
PCA score
A regions
Spearman r = 0.7 p<0.0001
B regions
Spearman r = -0.5 p<0.0001
cumulative gene expressions (Log2)
PCA score
A regions
Spearman r = 0.7 p<0.0001
B regions
Spearman r = -0.3 p<0.0001
J
DNA'se I HS in PCA-partitioned regions
A regions
B regions
Frequency (%)
DNA'se I HS per 10kb
DNA'seI HS frequency (Log2)
PCA score
A regions
Spearman r = 0.7 p<0.0001
B regions
Spearman r = -0.5 p<0.0001
K
SRF site locations
A regions
B regions
83%
17%
n = 2577
SRF sites frequency (Log2)
PCA score
A regions
Spearman r = 0.6 p<0.0001
B regions
Spearman r = -0.3 p<0.0001

Figure S2

A

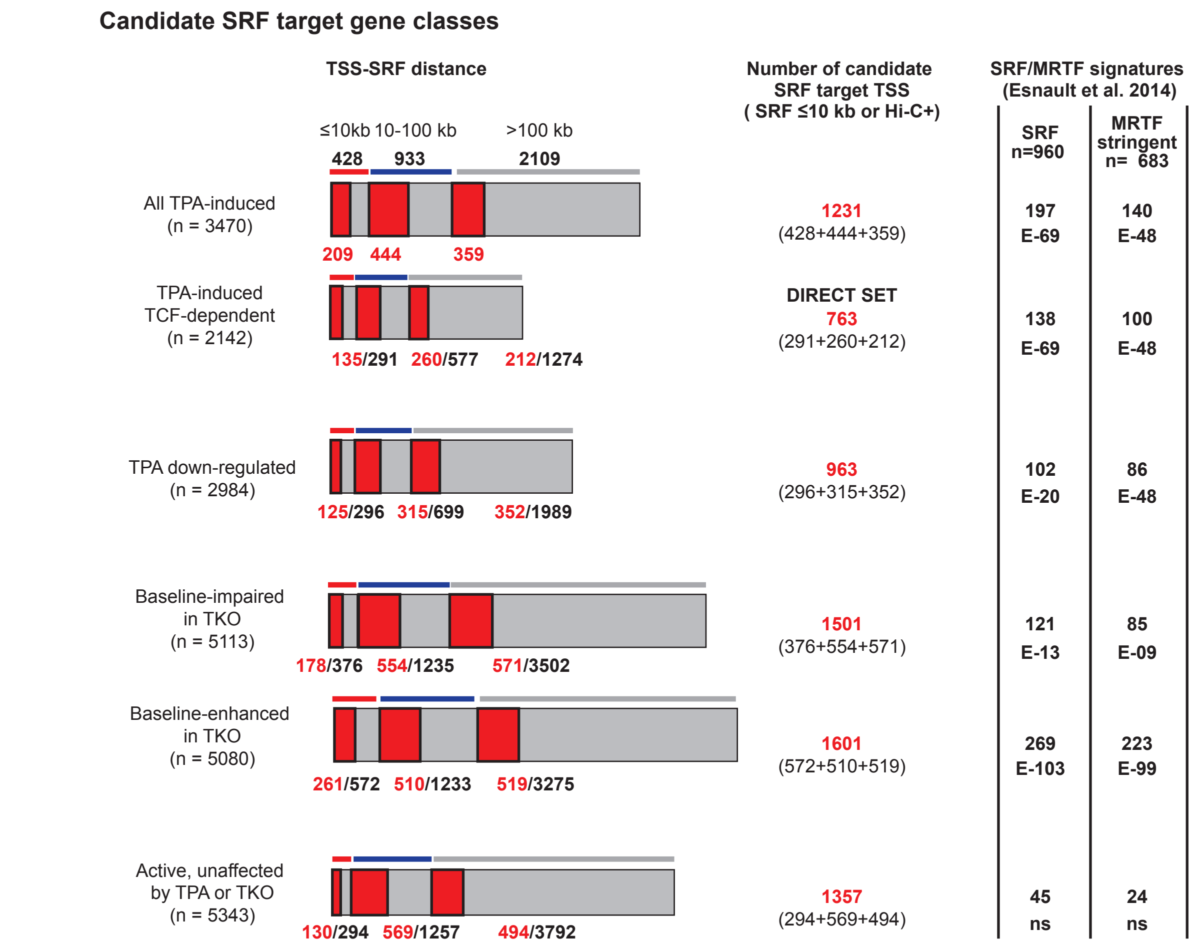

B

Relationship between TSS-associated gene function and SRF binding sites

	Relation of TSS-associated gene function to SRF ChIP-seq peaks in wildtype MEFs		Relation of TSS-associated gene function to SRF/Elk-1 ChIP-seq peaks in reconstituted TKO MEFs	
	Candidate SRF target TSS (≤10 kb from SRF or in Hi-C contact)	Number of SRF peaks associated with candidate target TSS (specific to gene class)	Candidate SRF/Elk-1 target TSS (≤10 kb from SRF/Elk-1 or in Hi-C contact)	Number of SRF/Elk-1 peaks associated with target TSS (specific to gene class)
All TPA-induced (n = 3470)	1231	964	239	119
TPA-induced TCF-dependent (n = 2142)	763	721	151 (94 rescued by Elk-1 o/ex)	89
TPA down-regulated (n = 2984)	963	1113 (28)	146	101 (0)
Baseline-impaired in TKO (n = 5113)	1501	1133 (113)	218	124 (13)
Baseline-enhanced in TKO (n = 5080)	1601	1275 (134)	252	136 (11)
Active, unaffected by TPA or TKO (n = 5343)	1357	953 (130)	222	102 (6)
		Unassigned: 585<br>Total: 2577		Unassigned: 47<br>Total: 251

Figure S3

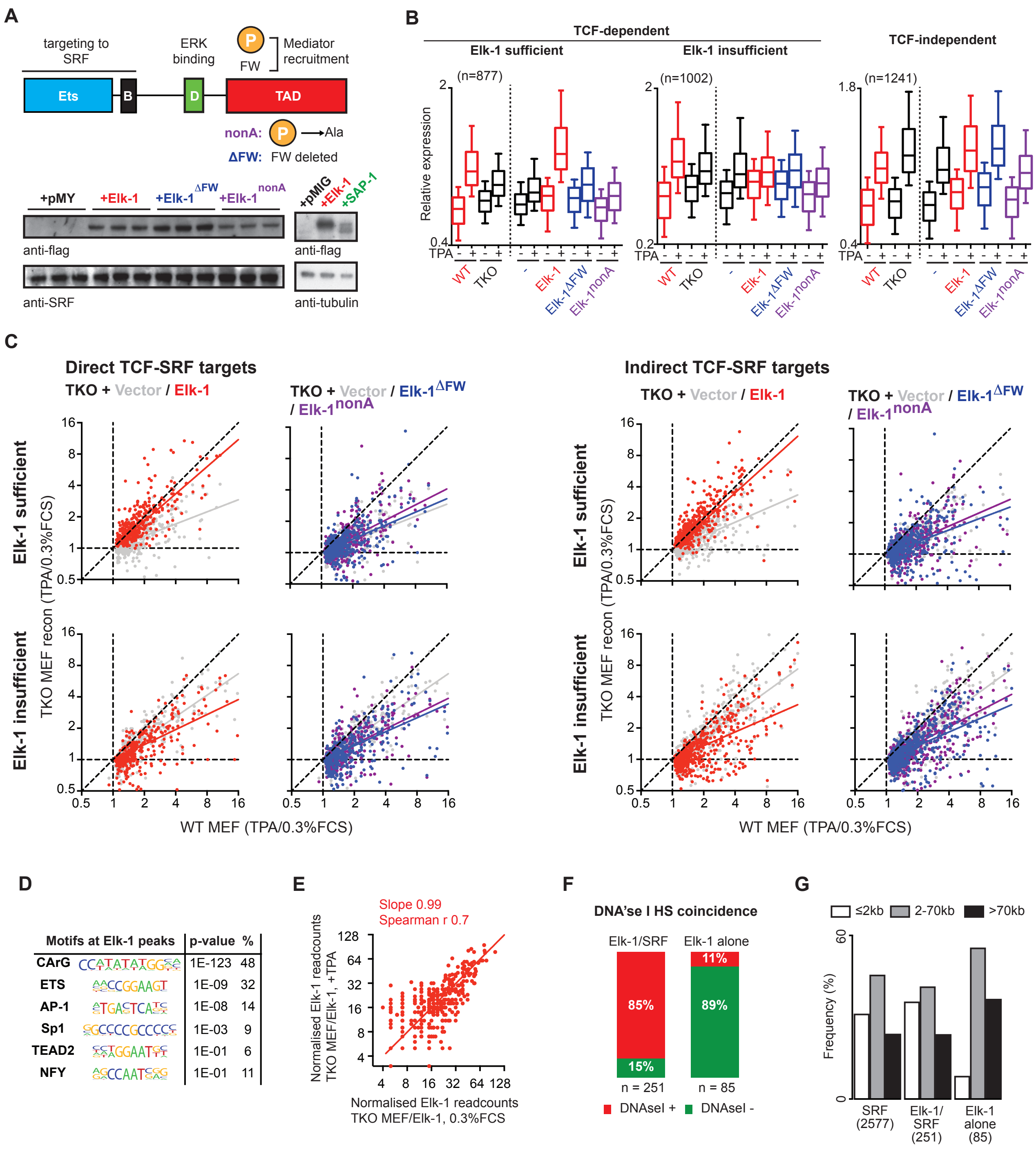
A
targeting to SRF
ERK binding
FW
Mediator recruitment
Ets
B
D
TAD
nonA:
Ala
ΔFW:
FW deleted
+pMY
+Elk-1
+Elk-1ΔFW
+Elk-1nonA
+pMIG
+Elk-1
+SAP-1
anti-flag
anti-SRF
anti-flag
anti-tubulin
B
TCF-dependent
Elk-1 sufficient
Elk-1 insufficient
TCF-independent
(n=877)
(n=1002)
(n=1241)
Relative expression
TPA
WT
TKO
-
Elk-1
Elk-1ΔFW
Elk-1nonA
C
Direct TCF-SRF targets
Indirect TCF-SRF targets
TKO + Vector / Elk-1
TKO + Vector / Elk-1ΔFW / Elk-1nonA
Elk-1 sufficient
Elk-1 insufficient
TKO MEF recon (TPA/0.3%FCS)
WT MEF (TPA/0.3%FCS)
D
Motifs at Elk-1 peaks | p-value | %
CArG | 1E-123 | 48
ETS | 1E-09 | 32
AP-1 | 1E-08 | 14
Sp1 | 1E-03 | 9
TEAD2 | 1E-01 | 6
NFY | 1E-01 | 11
E
Slope 0.99
Spearman r 0.7
Normalised Elk-1 readcounts TKO MEF/Elk-1, +TPA
Normalised Elk-1 readcounts TKO MEF/Elk-1, 0.3%FCS
F
DNA'se I HS coincidence
Elk-1/SRF
Elk-1 alone
85%
15%
11%
89%
n = 251
n = 85
DNAseI +
DNAseI -
G
≤2kb
2-70kb
>70kb
Frequency (%)
SRF (2577)
Elk-1/SRF (251)
Elk-1 alone (85)

Figure S4

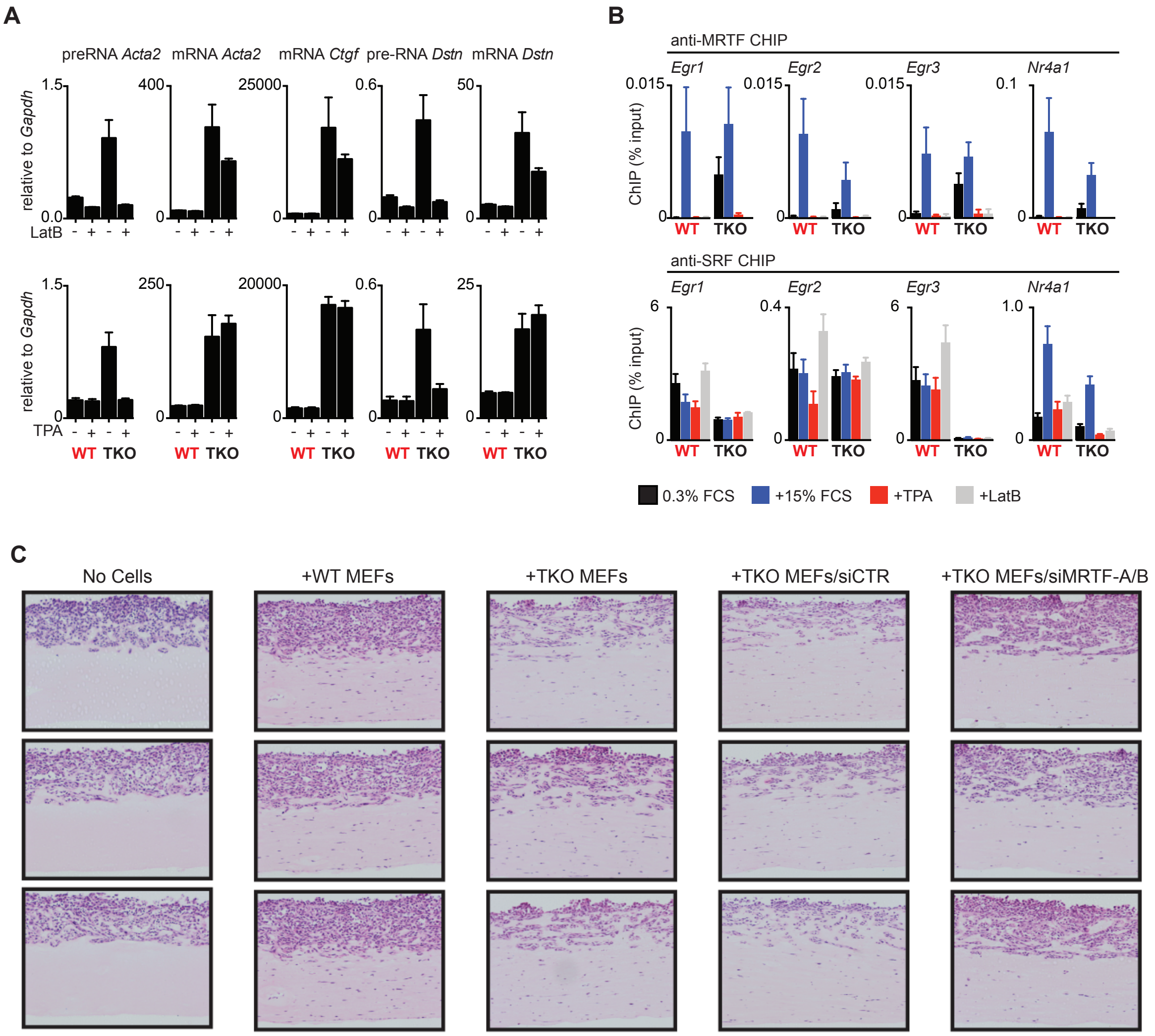
A
preRNA Acta2
mRNA Acta2
mRNA Ctgf
pre-RNA Dstn
mRNA Dstn
relative to Gapdh
LatB
TPA
WT TKO
B
anti-MRTF CHIP
anti-SRF CHIP
Egr1
Egr2
Egr3
Nr4a1
ChIP (% input)
0.3% FCS
+15% FCS
+TPA
+LatB
C
No Cells
+WT MEFs
+TKO MEFs
+TKO MEFs/siCTR
+TKO MEFs/siMRTF-A/B

Figure S5

SUPPLEMENTAL FIGURE LEGENDS

Figure S1| (related to Figure 1) TCF inactivation impairs IE gene induction and proliferation

(A) qRT-PCR analysis of IE gene transcription, normalised to GAPDH, in wildtype and TKO MEFs stimulated with TPA (red) or serum (blue). Data are means ± SEM, n=3.

(B) Proliferation is impaired in TKO MEFs. Cells were counted over a 4 day period. Data are means ± SEM, n=3 (****, p<0.0001, two-way ANOVA).

(C) TKO MEFs exhibit a prolonged G2/M phase. DNA content (propidium iodide staining) and BrdU incorporation (FITC staining) are plotted, with quantitation at right. ***, p<0.001; **, p<0.01; unpaired t test with Welch's correction.

(D) Elevated ERK activation persists following TPA stimulation of TKO MEFs, as assessed by immunoblotting, with quantation at right (mean ± SEM, n = 3).

(E) Normal kinetics but reduced magnitude of serum induction of IE genes in TKO MEFs. qRT-PCR analysis as in (A).

(F) Defective Egr1 gene induction is due to reduced response in all cells rather than lack of response in part of the population. FACS analysis of Egr1 protein accumulation following 1h TPA or serum stimulation of wildtype or TKO MEFs.

Figure S2 | (related to Figure 2) Integration of SRF ChIP-seq analysis of SRF binding in wildtype and TKO MEFs with MEF Hi-C data

(A) Metaprofiles of the 2577 SRF binding sites in wildtype (red) and TKO (black) MEFs.

(B) Metaprofiles of total H3 occupancy at either constitutive or TCF-enhanced SRF binding sites in wildtype and TKO MEFs.

(C) Distribution of SRF and Elk-1 peaks relative to Refseq annotated gene TSS. Dotted border denotes sites within ± 2Kb of the TSS.

(D) Distribution of the SRF sites around the TSS (inset: relative frequency).

(E) Most SRF sites coincide with DNA'se I HS sites. DNA'se I HS data is from GSM1003831, GSM1014199.

(F) SRF-associated transcription factor binding motifs within 100 bp of SRF peak summits

(G) Proportion of identified Hi-C interactions (~1.3 million total interactions identified at resolution of 10 kb resolution) contained within previously described TADs.

(H) Frequency distribution of the lengths of the regions defined by PCA analysis. Dotted grey limits display the theoretical ±1σ from the mean assuming a log-Gaussian distribution.

(I) Correlation of gene frequency and cumulative gene activity and PCA score, evaluated separately for A and B regions.

(J) Left, distribution of DNA’se I HS peak densities per 10kb in the A and B regions defined by PCA analysis. Right, correlation between DHS frequency per re region and PCA score, evaluated separately for A and B regions.

(K) Left, proportion of SRF ChIP-seq peaks contained in the A or B regions defined by PCA analysis; right, correlation of SRF site frequency per region, evaluated separately for A and B regions.

Figure S3 | (related to Figure 3, Tables S3, S4) Definition of potential SRF target genes

(A) Classes of potential SRF target gene are shown at left. Each grey block is scaled to the number of TSS in each class, and divided according to distance to the closest SRF site (<10kb, 10-100kb, >100kb). Red shading indicates that a TSS displays linkage to an SRF site in Hi-C. Not all interactions within 10kb of a TSS are detectable by our Hi-C analysis for technical reasons, and so potential SRF target genes are therefore defined as all those whose TSS are within 10kb of an SRF site, or that interact with one at any distance as judged by Hi-C. The relation between each target gene class and our previously defined SRF and MRTF signatures is shown at the right (hypergeometrical p-value and number of genes). The association of TPA-downregulated genes with SRF suggests a model in which these genes are controlled through MRTF and respond to downregulation of Rho-actin signalling by TPA (Panayiotou et al., 2016).

(B) Relationships between SRF binding sites and their neighbouring or interacting TSS. Note that 75-80% of SRF peaks can be associated with active genes. Left, all SRF sites; right, SRF/Elk-1 binding sites in TKO MEFs reconstituted with Elk-1. Gene classes are displayed as in (A), with the total number of linked SRF sites associated with those genes at the right. For classes of gene other than inducible ones, the number of sites associated only with genes of that class is indicated in brackets. Overall data can be retrieved by combining information contained in Tables S1, S4 and S7. The GRanges tools in R were used to assess both distances between TSSs and peaks and the match between peaks and hi-C anchor points. Of 1129 SRF sites with 10kb of a TSS, 625 were in Hi-C contact with other TSS, of which 299 were TPA-inducible and 174 TCF-dependent. In the Elk-1 reconstituted TKO MEFs, a total of 168 unique SRF/Elk-1 peaks out of the 251 total SRF/Elk-1 peaks were associated with genes whose basal level or induction was TCF-dependent. This number is likely an underestimate since some TCF-independent TSS may be false-negatives. It represents 67% of the total 251 SRF/Elk-1 peaks, and 82% of those peaks associated with active genes. As expected, no SRF/Elk-1 peaks were exclusively associated with TPA-downregulation.

Figure S4 | (related to Figure 4, Tables S3, S5) Restoration of TPA regulation in TKO MEFs by Elk-1 expression

(A) Expression of TCF derivatives in MEFs. Top, TCF functional domains. Mutant Elk-1^{nonA} contains alanine substitutions at nine conserved S/T-P

phosphoacceptor motifs, and mutant Elk-1$^{\Delta FW}$ deletes a conserved FW required for Mediator recruitment. Bottom, expression of TCFs in reconstituted TKO MEFs. Expression of Elk-1 mutants (lysates from three separate samples for each) and the Elk-1 and SAP-1 TCFs was analysed by immunoblot for the Flag epitope.

(B) Box plot summarising the effect of Elk-1 expression on signal-induced changes at TCF-dependent genes, partitioned according to whether Elk-1 expression restores regulation or not, and at TCF-independent genes.

(C) Scatter plots showing the relationship between gene induction in wildtype MEFs, and in TKO MEFs reconstitution with vector alone (grey), wildtype Elk-1 (red), Elk-1^{FW} (blue) or Elk-1^{nonA} (purple). Genes are partitioned according to whether wildtype Elk-1 expression is sufficient to rescue regulation.

(D) Elk-1 associated sequence motifs within 100 bp of Elk-1 peak summits.

(E) TPA stimulation does not affect Elk-1 DNA binding. Correlation of normalised Elk-1 ChIP-seq signals in resting and TPA stimulated cells.

(E) Relation between Elk-1 ChIP-seq signals before and after stimulation.

(F) Proportion of the Elk-1/SRF or Elk-1 coincident with 1 DNA'se I HS peak.

(G) Frequency distribution of the distance between SRF, Elk-1/SRF or Elk-1 solo ChIP-seq peaks and their closest TSS.

Figure S5 | (related to Figure 6) The TCFs antagonise MRTF-SRF dependent gene expression

(A) Elevated levels of MRTF-SRF target gene expression in TKO MEFs are sensitive to MRTF inhibition by Latrunculin B. RNA from cycling cells of cells were treated with LatB for 5 minutes was analysed by qRT-PCR. Error bars, SEM; n=4.

(B) Quantitative ChIP analysis of MRTF-A and SRF binding at regulatory elements the direct TCF target genes *Egr1*, *Egr2*, *Egr3* and *Nr4a1* in wildtype and TKO MEFs. Assays were from cells in resting conditions (0.3% FCS; black), after 30' serum or TPA stimulation (blue and black respectively), or after 5 min treatment with Latrunculin-B (grey). Error bars, SEM; n=4.

(C) TKO MEFs exhibit enhanced pro-invasive activity. Replicate assays showing the invasion of 4T1 breast carcinoma cells were allowed to invade a matrix composed of collagen I and Matrigel, that contained no fibroblasts, wildtype or TKO MEFs, or TKO MEFs treated with control or MRTF-A/B siRNAs as indicated. Images are from each of 3 independent invasion assays.

TABLE LEGENDS

Table S1 | (related to Figure 1) Genome wide description of TPA-induced gene changes and the role of the TCFs.

Columns 1-6. Gene description: Official gene symbol; transcript ID; Genomic coordinates (Chromosome, Strand, Start and Stop).

Column 7. Gene activity: active (1) or inactive (0).

Columns 8-10. Effects of 30 minute TPA stimulation, and dependence on TCF. TPA-induced (0 or 1) assessed by Deseq (see Materials and methods); TCF-dependence (0 or 1) assessed by regression method; TPA down-regulated genes (0 or 1) assessed by Deseq.

Column 11. Changes in basal expression for each gene comparing WT MEF with TKO MEF – up- regulated (UP), down- regulated (DOWN) or not changing (ns) assessed by Deseq.

Column 12-16. Relationship between each gene and the 2577 SRF ChIP-seq peaks, including losest peak coordinate; distance to the closest peak; coordinates of remote SRF peaks interacting with the gene TSS in HI-C; total number of remote SRF peaks interacting with the gene TSS in HI-C; genes considered as Linked (by Hi-C) or Near (within 10kb of an SRF peak).

Columns 17-23. Membership of the different gene sets described in Figure S3.

Column 23-26. Normalised total RNA readcounts in wildtype MEF and TKO MEF before and after TPA stimulation.

Column 27-30. Normalised intronic RNA read counts in wildtype MEF and TKO MEF before and after TPA stimulation.

Table S2 | (related to Figure 1) Overlap between global gene expression profiles in MEFs and (A) Hallmark gene signatures or (B) GO terms from MsigDB.

Column 1. (A) Gene signatures: the 53 gene Signature ID from MSigDB, together with SRF, MRTF and TCF signatures. (B) GO terms from MsigDB.

Columns 2-5. (A,B) Hypergeometrical test of each gene set (TPA-induced, TPA-downregulated, Baseline-enhanced in TKO, and Baseline-impaired in TKO) against each gene signature. The Bonferroni adjusted p-value is shown, and the number of genes in both sets.

Columns 6-9. (A,B) Gene name IDs for genes in both sets.

Table S3 | (related to Figure 3, Figure S3, S4) ChIP-seq analysis.

Columns 1-5. Peak coordinates for each of the 2577 SRF peaks and 336 Elk-1 peaks: Peak ID followed by the coordinate of the peak called by MACS and filtered using Deseq (Chromosome, Start, Stop, Peak summit coordinate).

Column 6-11. Peak information (1 or 0): called by SRF ChIP-seq (n=2577); enhanced by presence of the TCFs; called by Elk-1 ChIP-seq in reconstituted

TKO MEFs (n=336); w called in both SRF and Elk-1 ChIP-seq conditions; whether involved in HI-C interactions to TSSs; coincidence with DNA'se I HS.

Columns 12-20. Description of closest gene to the peak: Gene name; Activity; whether induced by TPA; whether TPA induction is TCF-dependent; whether Elk-1 expression in TKO MEFs is sufficient to restore regulation; TPA-downregulated; effect of TCF inactivation on basal expression; distance from peak to TSS; location of the peak (intergenic, intron, other gene feature...).

Column 21-26. Averaged normalised read counts across the various conditions: SRF ChIP-seq in wildtype and TKO MEFs; Elk-1 ChIP-seq in TKO MEFs reconstituted with Vector (negative control used in Deseq), wildtype Elk-1, Elk-1$^{\Delta FW}$, Elk-1^{nonA}.

Column 27-42. Normalised read counts in each condition.

Table S4 | (related to Figure 3, Figure S3) Overlap between SRF-linked gene sets and (A) Hallmark gene signatures or (B) GO terms from MsigDB.

Column 1. (A) Gene signatures: the 53 gene Signature ID from MSigDB, together with SRF, MRTF and TCF signatures. (B) GO terms from MSigDB.

Columns 2-10. (A,B) Hypergeometrical test against each gene signature for each set of genes whose TSS is in close proximity to or interacting with SRF in Hi-C unless otherwise stated. The Bonferroni adjusted p-value is shown, and the number of present in both gene sets. Gene sets analysed are: TPA-induced, linked to SRF; TPA-induced, Direct TCF-dependent targets; TPA-

induced, Indirect TCF-dependent targets (these are not SRF-linked); TPA-downregulated; Baseline-elevated in TKO MEFs; Baseline-elevated in TKO MEFs.

Columns 11-19. (A,B) Gene name IDs for genes in both sets.

Table S5 | (related to Figure 4, Figure S4) Reconstitution of TKO MEFs with Elk-1 derivatives.

This table is as Table S1 but focusses on TPA-regulated genes whose TSS close to and/or linked by Hi-C to SRF/Elk-1 or Elk-1 solo peaks.

Columns 1-6. Gene description: Official gene symbol; transcript ID; Genomic coordinates (Chromosome, Strand, Start and Stop).

Column 7. Gene activity: active (1) or inactive (0).

Columns 8-10. Effects of 30 minute TPA stimulation, and dependence on TCF. TPA-induced (0 or 1) assessed by Deseq (see Materials and methods);; TCF dependent (0 or 1) assessed by regression method; regulation restored by Elk-1 expression (0 or 1) assessed by regression method.

Columns 11-15. Relationship between each gene and the 2577 SRF peaks: closest peak coordinate; distance from TSS to the closest peak; remote SRF peak coordinates interacting with the gene TSS in Hi-C; number of remote SRF peaks interacting with the gene TSS in Hi-C; gene considered as SRF-linked (ie interacting by Hi-C or within 10kb of an SRF peak).

Column 16-20. Relationship between each gene and the 251 SRF/Elk-1 peaks: closest peak coordinate; distance from TSS to the closest peak; remote SRF/Elk-1 peaks coordinates interacting with the gene TSS in Hi-C; number of remote SRF/Elk-1 peaks interacting with the gene TSSin Hi-C; gene considered as SRF/Elk-1-linked (ie interacting by Hi-C or within 10kb of an SRF/Elk-1 peak).

Column 21-25. Relationship between each gene and the 85 Elk-1 solo peaks. Closest peak coordinate; distance from TSS to the closest peak; remote Elk-1 solo peak coordinates interacting with the gene TSS in Hi-C; number of remote Elk-1 solo peaks interacting with the gene TSS in Hi-C; gene considered as SRF-linked (ie interacting by Hi-C or within 10kb of an Elk-1 solo peak).

Column 26-41. Normalised total read counts and normalised read counts matching intronic features.

Table S6 | (related to Figure 4) ERK signalling to transcription factors encoded by Direct TCF-SRF target genes

The 54 TFs directly controlled by the TCFs are listed, with references and brief summary of the consequences of ERK signalling.

SUPPLEMENTAL METHODS

Cells

Mouse Embryonic fibroblasts (MEFs) were derived from wildtype mice (wildtype) or mice lacking the Elk-1, Net and SAP-1 TCFs (Elk1$^{-/-}$ Elk3$^{\delta/\delta}$ Elk4$^{-/-}$; TKO MEFs) and immortalized by infection with retrovirus expressing SV40 large T protein (Costello et al., 2010). MEFs were cultured in DMEM (Gibco, Invitrogen) supplemented with 10% FCS at 37°C and 10% CO_2. Cells were maintained in 0.3% FCS for 48 h, then stimulated with 12-O-Tetradecanoylphorbol-13-acetate (Sigma, TPA, 50ng/ml) for 30 minutes or as indicated in the Figure legends.

TKO MEF reconstitution using retroviral infection

TKO cells were reconstituted with human Elk-1 variants using retrovirus infection. Elk-1 wild type, Elk-1^{nonA} and Elk-1$^{\Delta FW}$ sequences (Cruzalegui et al., 1999; Marais et al., 1993; Price et al., 1995) were expressed using the retroviral vector pMYs (MSCV-(IRES)-EGFP, Cell Biolabs RTV-021); for comparative analysis, Elk-1 and SAP-1 were introduced using the pMIG (MSCV-IRES-GFP, Addgene 9044). GP2-293 cells were transfected with Elk-1 plasmids and pCMV-VSVG, using Fugene. Viral supernatants recovered 2-3 days later were used to infect Phoenix cells by spin infection. Viral supernatants were used to infect MEFs, which were sorted by FACS into

pools expressing GFP at low, medium and high level. Pools expressing similar amounts of Elk-1 derivatives were selected for further study.

qRT-PCR RNA analysis

For standard Q-PCR based gene expression analysis, cDNA synthesis used the SuperScript III first strand synthesis system with random hexamer primers (Invitrogen). SYBR Green-based real-time PCR (Invitrogen) was performed using dilutions of genomic DNA solution for calibration. The expression of each target was expressed relative to Gapdh.

The primer pairs used were : Gapdh: 5'- TCTTGTGCAGTGCCAGCCT-3', 5'-CAATATGGCCAAATCCGTTCA-3'; ACTA2: precursor 5'-CCAGAAGCAATGCGTCCACT-3', 5'-TGAGGTAGTTGCCTGCTCTC-3'; ACTA2: mRNA 5'-CTGTCAGGAACCCTGAGACGC-3', 5'-GGCTGTGCTGTCTTCCTCTT-3'; Ctgf: mRNA 5'-GGAGGAAAACATTAAGAAGGGCAA-3', 5'-AACTTGACAGGCTTGGCGAT-3'; Dstn: pre-RNA 5'-ATAGCAACTGGCTTGCAGGT-3', 5'-ACAGCTAAGCATGGTCCGTT-3'; Dstn: mRNA 5'-CGAACATGGCCTCAGGAGTT-'3, 5'-GGTGTGGAACATTTCCGAACTT-3'; Egr1: 5'-ATTGATGTCTCCGCTGCAGATC-3', 5'-TCAGCAGCATCATCTCCTCCA-3'; Egr2: 5'-GAGCAAATGATGACCGCCAA-3', 5'- TGTCAGGCAGCTGGTGCATAA-3'; Egr3: 5'-AGCCCAATCCGGAACTCTCTT-3', 5'-GGAAGGAGAGTCGAAAGCGAA-3';, Ier2: 5'-CCTGCGGTTCCTTTGTCCTTA-3', 5'-TCACTTTGGTTTCCGACATGC-3' , Per1: 5'-

AATAAGGCAGAGAGCGTGGTGTC-3’, 5’-TCTTGTCTCCCACATGGACGA-3’ , Junb: 5’-CCCTTCTCCCCTCCCTGTTA-3’, 5’-GTTTACATGGCCCCCTTCCA-3’ , Fos: 5’-TTCCTACTACCATTCCCCAGCC-3’, 5’-GATCTGCGCAAAAGTCCTGTGT-3’, Zfp36: 5’-CCAGGAGGCCAGATCTCTCT-3’, 5’-CACCTGTCTAGTGCCCTTCG-3’, Dnajb1: 5’-CCCGCTATAGTTCCTGTGGC-3’, 5’-TTCGGTTTCCCATGTGGAGG-3’, Arl5b: 5’-TGCACCTTCACGTTGTCTGA-3’, 5’-AGAAACCCGTGCACCTGAAT-3’, Pitpna: 5’-GGCAGCAGAGGAAAAGGGAT-3’, 5’-CCCTGTCATCCAGTCCCCTA-3’, Mob3a: 5’-TCTGCCCCATTGTAAAGCCA-3’, 5’-CTTGTCACTCCCTCGGTAGC-3’, Pramef8: 5’-TGTCTAGCTAGTGTTGCCCAC-3’, 5’-ACCAGCCCAAGCTTAACGAG-3’, Tada3: 5’-TGCCTCAGTCTATTTCCCCA-3’, 5’-GGTAATTCTCGATGCCCCCA-3’, Arhgap1 5’-CACTGTTGAGGGGGTTGACT-3’, 5’-CATAGAACCCAGACGCAGGA-3’, Arpc4: 5’-TACCTAGCTCTGGCACTGGT-3’, 5’-ATCCTGGCATGCACAACAGA-3’, Ier3: 5’-CCTCGAGTGGTGAGTATCGC-3’, 5’-CGTCAGATCGAAGGTCCCTG-3’, Klf10: 5’-AAATCCCTTGCAAGACCCCG-3’, 5’-CCCCTAAATAAGCCACGCCT-3’, Cxcl1: 5’-GCCACACTCAAGAATGGTCG-3’, 5’-GGGGTCATATGCCAGTACTCC-3’.

Total RNA preparation, library assembly and sequencing

Samples were prepared using GenElute Mammalian Total RNA Miniprep Kit (RTN350-1KT) and DNA contaminants were removed by DNA'se I treatment. The Ribo-zero rRNA removal kit (Epicentre) was used to remove ribosomal rRNA. RNA-seq libraries were prepared from at least 1μg RNA, using the directional mRNA-Seq Library Prep v1.0 Protocol (Illumina) with minor adjustments. To reduce volume of the PCR reactions, the Kapa HiFi HotStart ready mix, was substituted with the Illumina kit Phusion enzyme, the Agencourt AMPure XP beads being adjusted accordingly. The Illumina protocol PCR cycle was changed to match the quantity of the total RNA, measured using the Bioanalyser. Libraries were subject to cluster formation and then 72 base single end sequencing using a Hiseq analyser. Raw and processed data are online, associated with the GEO Series ID GSE75667.

RNA-Seq data analysis

The RNA-seq data were all aligned to the mm9 mouse genome using BWA (default settings). The reads were aligned to the gene features annotated in RefSeq. All reads within an annotated RefSeq gene ("All reads") and reads containing intronic sequences ("Intronic reads") were annotated using the bam file obtained by BWA as input. The data was then normalised against a set of invariant genes, identified using a method that assumed a quasi-normal distribution of gene readcounts. The RNAseq readcounts in each sample was expressed relative to one sample used as reference. Since the distribution of invariant gene readcounts should be approximatively Gaussian, we obtained

the μ_{diff} and σ_{diff} parameters of the best-fit Gaussian using the dnorm() function in R and optimised by maximum likelihood with the optim() function. Invariant genes were defined as those with differences in read counts within 1σ from the mean difference (μ_{diff}).

Differential gene expression (DGE) analysis was performed with Deseq (Anders and Huber, 2010) (comparing resting and TPA-induced, wildtype and TKO MEF samples adj-p ≤0.01, minimum change of 10%). We estimated the effect of knockout and reconstituted background by using a method that compares the degree of gene induction under two different conditions and identifies genes that are similarly affected by background. The basic assumption is that activity of genes dependent on a particular transcriptional regulator will be similarly affected by changes in abundance or activity of that regulator. The method does not require paired conditions; however, although it can give an overall estimate of the numbers of genes subject to shared control, it does not allow statistical conclusions to be drawn regarding particular genes.

Written in R, the pipeline compares the TPA-induced fold change in one background (x-axis: fold change in background 1), to the difference between the fold change in that background and a second background (y-axis: [fold-change in background 1]-[fold-change in background 2]). Systematic influence of the second background will generate a distribution asymmetrically disposed about y=0, and linear regression analysis of data points above and below y=0 should yield slopes that are significantly non-zero. For perfect dependence of the induction on the second background, linear regression of the data above y=0 should be 1. In contrast, differences between the signal in the two

backgrounds that arise from random technical variations will be distributed around y=0, and linear regression of points above or below y=0 should give a slope of zero. For each comparison, the linear fit for data-points with y>0 (impaired induction in the second background) and y<0 (enhanced induction in the second background) was compared to y=0. Data-points presenting a significant bias were then filtered through an iterative loop where each data-point was either included or excluded according to its Euclidean distance to the line y=0 or to the regression line going though the data-points under investigation.

To be classified as a TCF-SRF target, a gene has to satisfy several criteria: significant change in expression upon TPA stimulation; significant change in degree of induction upon TCF inactivation; and association with SRF binding sites identified by ChIP-seq, either within 10kb or exhibiting significant interaction with remote SRF sites according to Hi-C analysis. Since for each assay the FDR is below 5%, the overall FDR can be considered to be negligible.

Chromatin immunoprecipitation

ChIP was performed as described (Esnault et al., 2014; Miralles et al., 2003), with the following modifications: fixation was stopped by the addition of 250 mM glycine, sonication was performed with a Bioruptor® Plus and magnetic G-protein beads (Invitrogen) were used for recovery. SYBR Green-based real-time PCR (Invitrogen) was performed using dilutions of genomic DNA solution for calibration and to derive arbitrary abundance units. We assessed

SRF and MRTF DNA binding (see Figure 6 and S6) using primers : Acta2 5’-GAGGCCTGGGTCTCTTCCA-3’, 5’- GCTGAGCTGCCTCCTGTTTC-3’ ; Ankrd1 5’- ACCTACAGTCTCTTCCAAACCATGT-3’, 5’-CCAGTGAGCAGAGCAATTTCC-3’ ; Slc2a1 5’- CCTGCGCCCCTTACATCA -3’, 5’- GTGTTCCTGGTAGGGCTTGAAA -3’ ; Control (Zfp37) 5’-CCAGCAATGTGTGACTTGGATC-3’, 5’-TATTTCGAGCGCTGTGGCA-3’ ; Egr1_p: 5’-CGGAAACGCCATATAAGGAGC-3’, 5’-ATATAAGGCGCTGCCCAAA-3’; Egr2_p: 5’-AATCGTTCCT- GGCGAGCTC-3’, 5’-GCAGCTTTTGCCGTCACAT-3’; Egr3_p: 5’-AGTT CCCTGGCTGGGAGCT-3’, 5’-GCTGCTATCACCACCAACCA-3’, Nr4a1_p: 5’-CGGCTTGGTGAGCCTAGTG-3’, 5’-AAATAGTTTTCCTCTCGCCCG- 3’

Antibodies

Antibodies used were: SRF (sc-335, lot# D3013 S. Cruz); MRTF-A (sc-21558, lot# I1412, S. Cruz) Elk-1 anti-mouse Elk-1 aa309–429 was made in-house (Costello et al., 2010) and affinity-purified against recombinant human Elk-1^{nonA} (aa309-429) before use.

Chip-Seq sample preparation

DNA samples were end repaired, poly-A tailed and Illumina single-end adapters ligated following the standard Illumina protocol with minor adjustments. Agencourt AMPure XP beads at 0.8x ratio were used to remove adapter dimers after ligation. The Illumina kit Phusion enzyme was replaced

by Kapa HiFi HotStart ready mix. Post PCR, AMPure XP beads were used at a 1:1 ratio to maintain size integrity. DNA fragments size-selected and purified from a 2% agarose gel using the QIAquick gel extraction kit, quality controlled on the DNA 1000 BioAnalyser 2100 chip. Sequencing was on the Hi-seq2500 to generate 150 base read lengths.

ChIP-seq analysis

All ChIP-seq samples have been aligned using BWA to the mm9 (default settings). Raw and processed data are online on GEO, Series ID GSE75667. Candidate SRF and Elk-1 sites were first identified using MACS (Zhang et al., 2008) to identify regions enriched over background (beads alone) in each aligned sample. Default settings and MACS threshold values of p<1E-4 were used. Peaks were merged with basic functions in bedtools, obtaining ~1/2 million unique peaks identified in any of the 8 SRF samples (SRF ChIP in wildtype MEF and TKO MEF in 0.3%FCS and TPA, two replicates per condition per background) and in any of the 6 Elk-1 samples (Elk-1 ChIP-seq perfomend in TKO MEF reconstituted with Elk-1^{WT}, Elk-1^{FW} or Elk-1^{nonA} in 0.3% FCS and TPA, one replicate per condition per background).

To identify ChIP-seq peaks, we first filtered peak set according to peak concidence, retaining those peaks called by MACS at p<1E-4 in at least 3 pseudo-replicate samples (3 out of the 8 SRF samples and 3 out of the 6 Elk-1 samples), giving a total of ~20000 unique peaks. The rationale for using occurence of a peak-call in 3 pseudoreplicates to assign a candidate ChIP-

seq peak was based on an assessment of the probability that overlap of MACS-called peaks occurs by chance. To do this, we iteratively simulated in R randomly distributed ChIP-seq peaks across the mm9 genome, considering a series of parameters was considered including: frequency distribution of the peak width observed across the ~1/2 million peaks; the number of peaks called per chromosome, and therefore each chromosome length and the number of peaks called per sample. We generated ~1/2 million random peak coordinates fullfilling the above criteria and counted how often out of 10000 iterations any given coordinate was overlapping with 1, 2, 3 or more peaks. This yielded empirical estimated FDRs for the coincidence of MACS-called peaks as: 0.5E-2 for one, 1E-3 for 2, 2.5E-5 for 3 and 3.4E-6 for 4 coincidences. We therefore chose 3 peak coincidences as our filter criterion.

Tools which allow identification of ChIP-seq peaks are prone to false positives (Krebs et al., 2014) owing to signal-to-noise-ratio and variables such as antibody affinity and specificity. To assess the validity of the identified ~20000 peaks we therefore assessed whether the read distribution in SRF and Elk-1 ChIP-seq samples was substantially different from ChIP-seq of Elk-1 in TKO MEF transduced with empty vector. These ChIP-seq samples are the perfect control for Elk-1 ChIP-seq, and it is also valid to assume that SRF ChIP-seq peaks should present read-counts which distribute differently from those generated by an unrelated antibody in cells lacking the cognate epitope. Comparison of the read counts was performed using Deseq (minimal increase relative to control of 1.5-fold, $p \leq 0.05$) comparing the control Elk-1 ChIP-seq (two pseudo-replicates: performed in TKO MEF rescued with Vehicle in 0.3% FCS and TPA-stimulated conditions) to the 6 Elk-1 ChIP-seq samples or to

the SRF samples. The read counts showed a skewed distribution. Of the ~20000 peaks, 2662 unique peaks passed this test, with 2577 showed significant signal for SRF and 336 showed signal for Elk-1. We used the same approach to identify those SRF peaks which dropped in the TKO MEF if compared to wildtype MEFs, comparing the 4 SRF CHIP-seq pseudo-replicates in wildtype MEF and TKO MEFs. Together this approach will give an effectively negligible FDR.

Identification of TF Binding Motifs

Discovery of known motif was performed using HOMER (Heinz et al., 2010) on sequences ±100 bp from the peak summit of ChIP-seq identified regions or DNAseI hypersensitive sites.

Hi-C analysis

The HOMER Hi-C software analysis pipeline (http://biowhat.ucsd.edu/homer/interactions/) was used to determine significant interactions and perform PCA compartment analysis. Paired-end reads from 3 different datasets were used: SRX554530 (Battulin et al., 2015), GSM1648486 and GSM1696042 (Minajigi et al., 2015) Reads were aligned independently to the mouse reference genome assembly (NCBI37/mm9) using Bowtie2 (Li and Durbin, 2009) by the iterative method (Imakaev et al., 2012), starting with a minimum length of 26bp. Each read was then trimmed to have equal length relative to 5’, and unmapped and unpaired reads were removed. Reads were then filtered to remove duplicate read pairs (“-tbp 1”),

paired-end reads likely representing continuous genomic fragments or religation events (‘‘-removePEbg’’) and self-ligations (‘‘-removeSelfLigation’’). This approach generated a total of ~460 million uniquely mapped PE reads. Of these only read-pairs where both ends mapped near the HindIII (AAGCTT) restriction sites were retained (‘‘-both’’) using a distance threshold as 1.5x the fragment length estimated with Homer tools.

To identify interactions, we first determined a ‘‘universe’’ of interactions if compared to the background model, therefore taking into account linear genomic distance and sequencing depth (p ≤0.05 and z-score ≥2). The identification of interaction was performed looking at overlapping windows (“-res 5000” and “-superRes 10000”), allowing removal of redundant interactions and retention of those that are significant. Each anchor point was re-centred according to the average of the positions contributing to it (“-center”). This analysis identified ~1.3 million significant interactions at 10kb resolution. Interactions mapping at all Refseq annotated TSSs (~130000) were retrieved using the annotateInteractions.pl pipeline in Homer.

Chromosomal regions exhibiting preferential interactions were identified by using the automated PCA analysis on Hi-C data in HOMER (runHiCpca.pl) based on the Lieberman-Aiden strategy to identify minimal components that describe most of the high dimensional dataset (Lieberman-Aiden et al., 2009). These regions, denoted A and B according to PCA score, are analogous to the previously defined A and B compartments (Lieberman-Aiden et al., 2009). They are defined as those with high contacts *in cis* and low contacts i*n trans* as extensively described in the HOMER website (http://homer.salk.edu/homer/interactions/HiCpca.html). The identified

interactions obtained with the runHiCpca.pl script were then refined to reduce small PCA inversions. We assigned a score per region as function of its average absolute PCA score and its length (abs(Homer average PCA score) * region length/10kb). As the resolution used is 10kb, this represents the smallest region length. Regions with a length-normalised PCA score of >100 were retained, and those scoring below this were merged with their neighbours according to their PCA sign.

Cell morphology, ECM-remodelling and invasion assays.

Highthroughput imaging was used to quantify actin stress fibre length. F-actin were stained with phalloidin, and fibre length (contiguous pixels emitting light) analysed using the Cellomics ArrayScan VTI and Compartmental analysis (BioApplications). 3000 cells were counted per determination.

Force-mediated matrix remodelling (Calvo et al., 2013) was performed by embedding $50x10^3$ fibroblasts in 100μl of collagen I/Matrigel and seeded on a 35-mm glass-bottom MatTek dish (P35-1.5-14-C, MatTek). Once the gel was set, the fibroblasts embedded gel was manteined in DMEM+10% FCS with + 1% ITS (insulin–transferrin–selenium; #41400-045; Invitrogen. Gel sizes were obtained using ImageJ software, and gel contraction values represent % of original gel size 10-12h after seeding.

The fibroblast organotypic culture system was set up as described (Calvo et al., 2013) with minor modifications. $5x10^5$ fibroblasts were embedded in a mixture of collagen I/Matrigel matrix and 1ml of this mix was set into 24 well-plates. After setting the gel at 37 °C for 1 h, DMEM+10% FCS+1% ITS was

added on the top. The day after 5×10^5 4T1 breast cancer cells were seeded on top of each gel in DMEM +10% FCS+1% ITS. The day after the gel was mounted on a metal bridge and fed from underneath with DMEM +10% FCS + 1% ITS (changed daily). After 5 days, the cultures were fixed in 4% paraformaldehyde plus 0.25% glutaraldehyde in PBS and processed by standard methods for haematoxylin and eosin staining.

SUPPLEMENTAL REFERENCES

Anders, S., and Huber, W. (2010). Differential expression analysis for sequence count data. Genome Biol *11*, R106.

Battulin, N., Fishman, V.S., Mazur, A.M., Pomaznoy, M., Khabarova, A.A., Afonnikov, D.A., Prokhortchouk, E.B., and Serov, O.L. (2015). Comparison of the three-dimensional organization of sperm and fibroblast genomes using the Hi-C approach. Genome Biol *16*, 77.

Calvo, F., Ege, N., Grande-Garcia, A., Hooper, S., Jenkins, R.P., Chaudhry, S.I., Harrington, K., Williamson, P., Moeendarbary, E., Charras, G., *et al.* (2013). Mechanotransduction and YAP-dependent matrix remodelling is required for the generation and maintenance of cancer-associated fibroblasts. Nature cell biology *15*, 637-646.

Costello, P., Nicolas, R., Willoughby, J., Wasylyk, B., Nordheim, A., and Treisman, R. (2010). Ternary complex factors SAP-1 and Elk-1, but not net, are functionally equivalent in thymocyte development. J Immunol *185*, 1082-1092.

Cruzalegui, F.H., Cano, E., and Treisman, R. (1999). ERK activation induces phosphorylation of Elk-1 at multiple S/T-P motifs to high stoichiometry. Oncogene *18*, 7948-7957.

Esnault, C., Stewart, A., Gualdrini, F., East, P., Horswell, S., Matthews, N., and Treisman, R. (2014). Rho-actin signaling to the MRTF coactivators dominates the immediate transcriptional response to serum in fibroblasts. Genes Dev *28*, 943-958.

Heinz, S., Benner, C., Spann, N., Bertolino, E., Lin, Y.C., Laslo, P., Cheng, J.X., Murre, C., Singh, H., and Glass, C.K. (2010). Simple combinations of lineage-determining transcription factors prime cis-regulatory elements required for macrophage and B cell identities. Molecular cell *38*, 576-589.

Imakaev, M., Fudenberg, G., McCord, R.P., Naumova, N., Goloborodko, A., Lajoie, B.R., Dekker, J., and Mirny, L.A. (2012). Iterative correction of Hi-C data reveals hallmarks of chromosome organization. Nat Methods *9*, 999-1003.

Krebs, W., Schmidt, S.V., Goren, A., De Nardo, D., Labzin, L., Bovier, A., Ulas, T., Theis, H., Kraut, M., Latz, E.*, et al.* (2014). Optimization of transcription factor binding map accuracy utilizing knockout-mouse models. Nucleic Acids Res *42*, 13051-13060.

Li, H., and Durbin, R. (2009). Fast and accurate short read alignment with Burrows-Wheeler transform. Bioinformatics *25*, 1754-1760.

Lieberman-Aiden, E., van Berkum, N.L., Williams, L., Imakaev, M., Ragoczy, T., Telling, A., Amit, I., Lajoie, B.R., Sabo, P.J., Dorschner, M.O.*, et al.* (2009). Comprehensive mapping of long-range interactions reveals folding principles of the human genome. Science *326*, 289-293.

Marais, R., Wynne, J., and Treisman, R. (1993). The SRF accessory protein Elk-1 contains a growth factor-regulated transcriptional activation domain. Cell *73*, 381-393.

Minajigi, A., Froberg, J.E., Wei, C., Sunwoo, H., Kesner, B., Colognori, D., Lessing, D., Payer, B., Boukhali, M., Haas, W.*, et al.* (2015). Chromosomes. A comprehensive Xist interactome reveals cohesin repulsion and an RNA-directed chromosome conformation. Science *349*.

Miralles, F., Posern, G., Zaromytidou, A.I., and Treisman, R. (2003). Actin dynamics control SRF activity by regulation of its coactivator MAL. Cell *113*, 329-342.

Panayiotou, R., Miralles, F., Pawlowski, R., Diring, J., Flynn, H., Skehel, M., and Treisman, R. (2016). Phosphorylation acts positively and negatively to regulate MRTF-A subcellular localisation and activity. eLife *5*, e15460.

Price, M.A., Rogers, A.E., and Treisman, R. (1995). Comparative analysis of the ternary complex factors Elk-1, SAP-1a and SAP-2 (ERP/NET). EMBO J *14*, 2589-2601.

Zhang, Y., Liu, T., Meyer, C.A., Eeckhoute, J., Johnson, D.S., Bernstein, B.E., Nusbaum, C., Myers, R.M., Brown, M., Li, W.*, et al.* (2008). Model-based analysis of ChIP-Seq (MACS). Genome Biol *9*, R137.
